# Loss of fungal symbionts and changes in pollinator availability caused by climate change will affect the distribution and survival chances of myco-heterotrophic orchid species

**DOI:** 10.1038/s41598-023-33856-y

**Published:** 2023-04-26

**Authors:** Marta Kolanowska

**Affiliations:** Department of Geobotany and Plant Ecology, Faculty of Biology and Environmental Protection, University of Lodz, Banacha 12/16, 90-237 Lodz, Poland

**Keywords:** Biodiversity, Climate-change ecology, Conservation biology, Ecology, Plant sciences, Climate sciences, Ecology

## Abstract

The first comprehensive species distribution models for orchid, its fungal symbionts and pollinator are presented. To evaluate impact of global warming on these organisms three different projections and four various climate change scenarios were analysed. The niche modelling was based on presence-only records of *Limodorum abortivum*, two species of *Russula* and three insects pollinating orchid (*Anthophora affinis, Bombus terrestris, Rhodanthidium septemdentatum*). Two sets of orchid predictions were examined—the first one included only climatic data and the second one was based on climate data and data on future distribution of orchid fungal symbionts. Overall, a poleward range shift is predicted to occur as a result of climate change and apparently global warming will be favorable for *L. abortivum* and its potential geographical range will expand. However, due to the negative effect of global warming on fungal symbionts of *L. abortivum*, the actual extension of the suitable niches of the orchid will be much limited. Considering future possibility of cross-pollination, the availability of *A. affinis* for *L. abortivum* will decrease and this bee will be available in the worst case scenarios only for 21% of orchid populations. On the other hand, the overlap of orchid and the buff-tailed bumblebee will increase and as much as 86.5% of plant populations will be located within *B. terrestris* potential range. Also the availability of *R. septemdentatum* will be higher than currently observed in almost all analysed climate change projections. This study showed the importance of inclusion of ecological factors in species distribution models as the climate data itself are not enough to estimate the future distribution of plant species. Moreover, the availability of pollen vectors which is crucial for long-term survival of orchid populations should be analysed in context of climate changes.

## Introduction

Climate is the most important factor determining species distribution^1^. To understand how the conditions of our planet changed over time various research teams developed climate models (general circulation models, GCMs) describing past^2^, present^3^ and future^4^ climatic Earth conditions. These GCMs became important tool in biogeographic^5–9^, phylogenetic^10,11^ and ecological^12–15^ research. Species distribution models based on GCMs are also used in nature conservation planning and preventing invasive species expansion^16–19^. Considering possible negative impact of climate change modification of temperature and precipitation patterns together with extreme weather events can alter geographical ranges of species^20^, their ecological interactions^21^, and the timing of biological events (phenology), which could fundamentally transform ecosystems and food webs^22^.

Clearly, any environmental change is potentially more dangerous for specialized organisms characterized by narrow ecological tolerance and depending on numerous interspecific interactions. Due to the complex life cycle Orchidaceae are among the most threatened plants in the global scale^23^. According to the International Union for the Conservation of Nature (IUCN) almost half of the extinct orchid species are terrestrial herbaceous perennials^24^. All Orchidaceae develop mycorrhizal symbioses with fungi in natural habitats that affect not only their seed germination, but also protocorm growth, and adult nutrition^25^. Orchids interact with a more limited set of mycorrhizal fungi as compared to other mycorrhizal plants^25^. Although many initial mycoheterotrophs develop into autotrophic mature plants, some orchids continue to obtain carbon from mycorrhizal fungi throughout their lifecycle (full mycoheterotrophy) or utilize both photosynthesis and mycoheterotrophy at maturity (partial mycoheterotrophy, mixotrophy)^26,27^. Generally, orchid flowers are adapted to promote cross-pollination and most species depend on pollen vectors for reproduction^28–31^. Autogamy is not common in Orchidaceae and this mode of reproduction evolved only in about 5–20% of the family representatives^32–34^.

One of the very interesting orchid species which is strongly associated with fungi not only during seed germination, but also as mature plant, and which is adapted to both cross-pollination and autogamy is *Limodorum abortivum*, also known as the Violet Limodore. This terrestrial plant does not produce any basal leaves and the upper, cauline ones are modified into sheathing bracts. Whitish-violet flowers of *L. abortivum* are arranged in a long racemose inflorescence. The Violet Limodore is a myco-heterotrophic plant and so far three species of *Russula* (*R. brevipes, R. chloroides, R. delica*) were found to be *L. abortivum* fungal symbionts compensating insufficient plant CO_2_ fixation^35^. Flowers of *L. abortivum* are considered to be mostly cleistogamous^36^, but some pollination events were recorded. So far three pollen vectors of the Violet Limodore were identified—two bee species (*Anthophora biciliata* and *Rhodanthidium septemdentatum*) and the buff-tailed bumblebee (*Bombus terrestris*)^37^. *Limodorum abortivum* is native to mainland Europe, western Asia and the Mediterranean area. According to the IUCN it is a species of Least Concern^38^ but in numerous geographical areas it is considered to be endangered (Critically Endangered^39,40^, Endangered^41^, Vulnerable^42^ or Near Threatened^43,44^).

The aim of this study was to evaluate the importance of fungal symbionts presence in the future distribution of *L. abortivum* and to estimate fluctuations in the availability of orchid pollen vector under various climate change scenarios using ecological niche modelling.

## Methods

### List of localities

The database of records of *L. abortivum*, its fungal symbionts and pollinators was compiled based on data included in the public catalogue—Global Biodiversity Information Facility (GBIF). The initial datasets (Supplementary Annex 1) were verified and only records correctly georeferenced with a precision of at least 1 km were analysed further. Because previous studies^45,46^ indicated that usage of a restricted area in ENM analysis is more reliable than modelling on a global scale, the area studied was limited to 23.42S-65.64ºN, 13.96ºW-57.41ºE.

**Figure 1:**
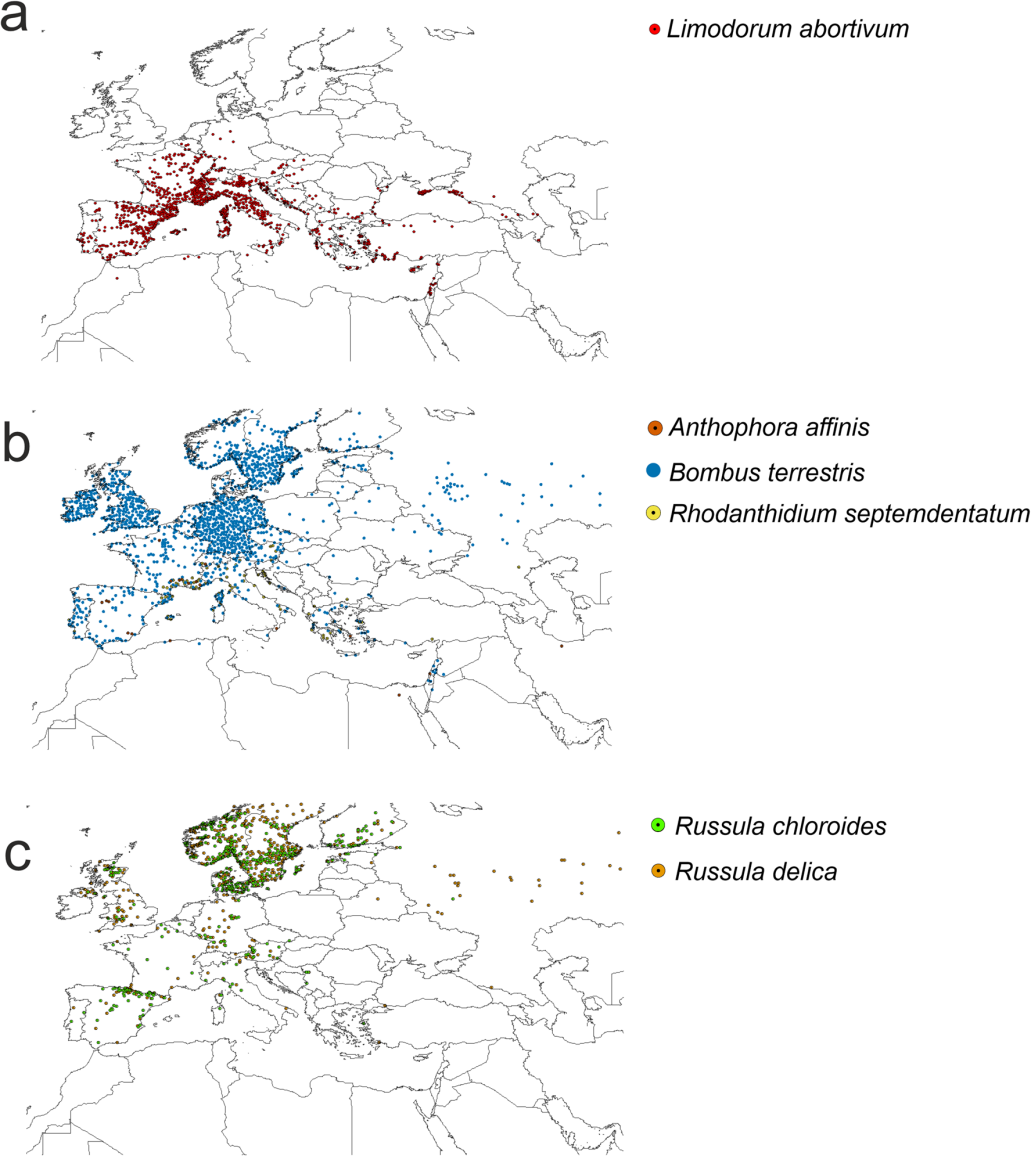
Localities of analysed species used in ENM analyses. (**a**) *Limorodum abortivum*, (**b**) pollinators, (**c**) fungal symbionts. Maps generated by the author in ArcGIS^47,48^.

Spatial thinning was conducted using SDMtoolbox 2.3 for ArcGIS^47,48^ to reduce the spatial bias of sampling^49–51^. While geographic and environmental thinning are conceptually equivalent, as they use a distance measure to determine a filter size^52^, they can lead to a disproportionate sampling in the environmental space. For that reason the additional factor, topography, was included in the spatial thinning process. The topographic heterogeneity of the study area was calculated^53^ and divided into five classes. Localities were spatially filtered at 5.0, 10.0, 15.0, 20.0 and 25.0 km in areas of high, medium high, medium, medium low and low heterogeneity, respectively. The final database of localities included 1074 records of *L. abortivum*, 19 of *A. affinis*, 1440 of *B. terrestris*, 103 of *Rhodanthidium septemdentatum*, 388 of *R. chloroides*, and 606 of *R. delica* (Fig. 1, Supplementary Annex 2)*.*

### Ecological niche modelling

Two models were used to estimate the effect of global warming on *L. abortivum*. The first was based exclusively on bioclimatic variables (bioclims) and the second included the modelled distributions of the fungal symbionts (bioclims + fungi).

MaxEnt v. 3.3.2^54,55^ which is considered to be one of the best-performing niche modeling algorithm based on presence-only data^56,57^ was used to produce the ecological niche model of the species studied. The algorithm selection was also motivated by the scarce information on distribution of studied organisms and lack of possibility of creating reliable pseudo-absence data. Due to this obstacle none of the existing presence-absence modelling methods could be used^58^. For the analyses bioclimatic variables (bioclims) in 30 arc-seconds of interpolated climate surface downloaded from WorldClim v. 2.1^3^ were used. Pearsons’ correlation coefficient was calculated using SDMtoolbox v. 2.3 for ArcGIS^47,48^ and highly correlated bioclimatic variables (above 0.9; Supplementary Annex 3) were removed from the analyses (Table 1).

**Table 1:** Bioclimatic variables. Layers used in ENM analyses marked with ‘ + ”.

Code	Description	ENM
bio1	Annual mean temperature	+
bio2	Mean diurnal range [mean of monthly (max temp—min temp)]	+
bio3	Isothermality (bio2/bio7) (× 100)	+
bio4	Temperature seasonality (standard deviation × 100)	+
bio5	Max temperature of warmest month	
bio6	Min temperature of coldest month	
bio7	Temperature annual range (bio5-bio6)	+
bio8	Mean temperature of wettest quarter	+
bio9	Mean temperature of driest quarter	+
bio10	Mean temperature of warmest quarter	
bio11	Mean temperature of coldest quarter	
bio12	Annual precipitation	+
bio13	Precipitation of wettest month	
bio14	Precipitation of driest month	+
bio15	Precipitation seasonality (coefficient of variation)	+
bio16	Precipitation of wettest quarter	
bio17	Precipitation of driest quarter	
bio18	Precipitation of warmest quarter	+
bio19	Precipitation of coldest quarter	+

To evaluate the impact of global warming on the distribution of species studied predictions of the extent of the climatic niches of *L. abortivum,* its symbionts and pollinator in 2080–2100 were made. The models were compiled using climate projections for four Shared Socio-economic Pathways (SSPs): SSP1-2.6, SSP2-4.5, SSP3-7.0 and SSP5-8.5. These trajectories were established to describe probable major global developments that would lead in the future to different challenges for mitigation and adaptation to climate change^59^. The SSPs are based on narratives describing alternative socio-economic developments (sustainable development, regional rivalry, inequality, fossil-fueled development, middle-of-the-road development) with global warming in 2100 ranging from of 3.1ºC to 5.1ºC above pre-industrial levels^60,61^. Three different simulations of future climate developed by Coupled Model Intercomparison Project Phase 6 (CNRM-CM6-1), Goddard Institute for Space Studies (GISS-E2-1), and Institute for Numerical Mathematics (INM-CM5) were used. While some Global Climate Models (GCMs) were proved to work better in specific geographical regions^62^, the area in this study included whole Europe and north Africa, hence it was not possible to select one, best-performing simulation. For that reason the three aforementioned GCMs which significantly differ in the simulated maximum temperature and precipitation within study area were used. This approach allows to present the broadest spectrum of possible changes in the distribution of studied organisms.

In all analyses the maximum number of iterations was set to 10,000 and convergence threshold to 0.00001. The neutral regularization multiplier value and auto features were utilized. The random test partition and background subset for each run was applied using 30% of the samples as test points. The run was performed as a bootstrap with 100 replicates, and the output was set to logistic. All analyses of GIS data were carried out using ArcGis v. 10.8 (Esri, Redlands, CA, USA). To prevent extrapolations outside the environmental range of the training data the *“fade by clamping”* function in MaxEnt was used^63–65^. The evaluation of the created models was made using the area under the curve (AUC)^66^ and True Skill Statistic (TSS)^67^. Both metrics are frequently used measure of model performance as they are independent of prevalence^68^.

SDMtoolbox v. 2.3 for ArcGIS^47,48^ was used to calculate changes in the distribution of suitable niches of *L. abortivum* caused by global warming. For this operation created models (for present-time and future) were converted into binary rasters in Goode homolosine projection. The max kappa value was used as presence threshold and used to compare the extent and location of suitable niches of studied species between present-time and future models. To calculate max kappa value a phyloclim package for R was used^69^.

## Results

### Models evaluation and limiting factors

The model performance indexes (AUC and TSS) are presented in Table 2 together with Max Kappa value which was used as a presence threshold. The AUC values of 0.875–0.971 indicate high reliability of the models, however, the TSS for *Anthophora affinis* and *Bombus terrestris* received lower scores and these models should be taken with caution.

**Table 2:** Modelling performance indexes.

Species	AUC (Standard Deviation)	TSS	MaxKappa
*Anthophora affinis*	0.933 (SD = 0.024)	0.493	0.325
*Bombus terrestris*	0.875 (SD = 0.003)	0.589	0.365
*Rhodanthidium septemdentatum*	0.971 (SD = 0.006)	0.833	0.437
*Limodorum abortivum* (bioclims only)	0.927 (SD = 0.001)	0.775	0.434
*Limodorum abortivum* (bioclims + fungi)	0.931 (SD = 0.002)	0.794	0.439
*Russula chloroides*	0.935 (SD = 0.004)	0.716	0.407
*Russula delica*	0.916 (SD = 0.004)	0.659	0.385

**Figure 2:**
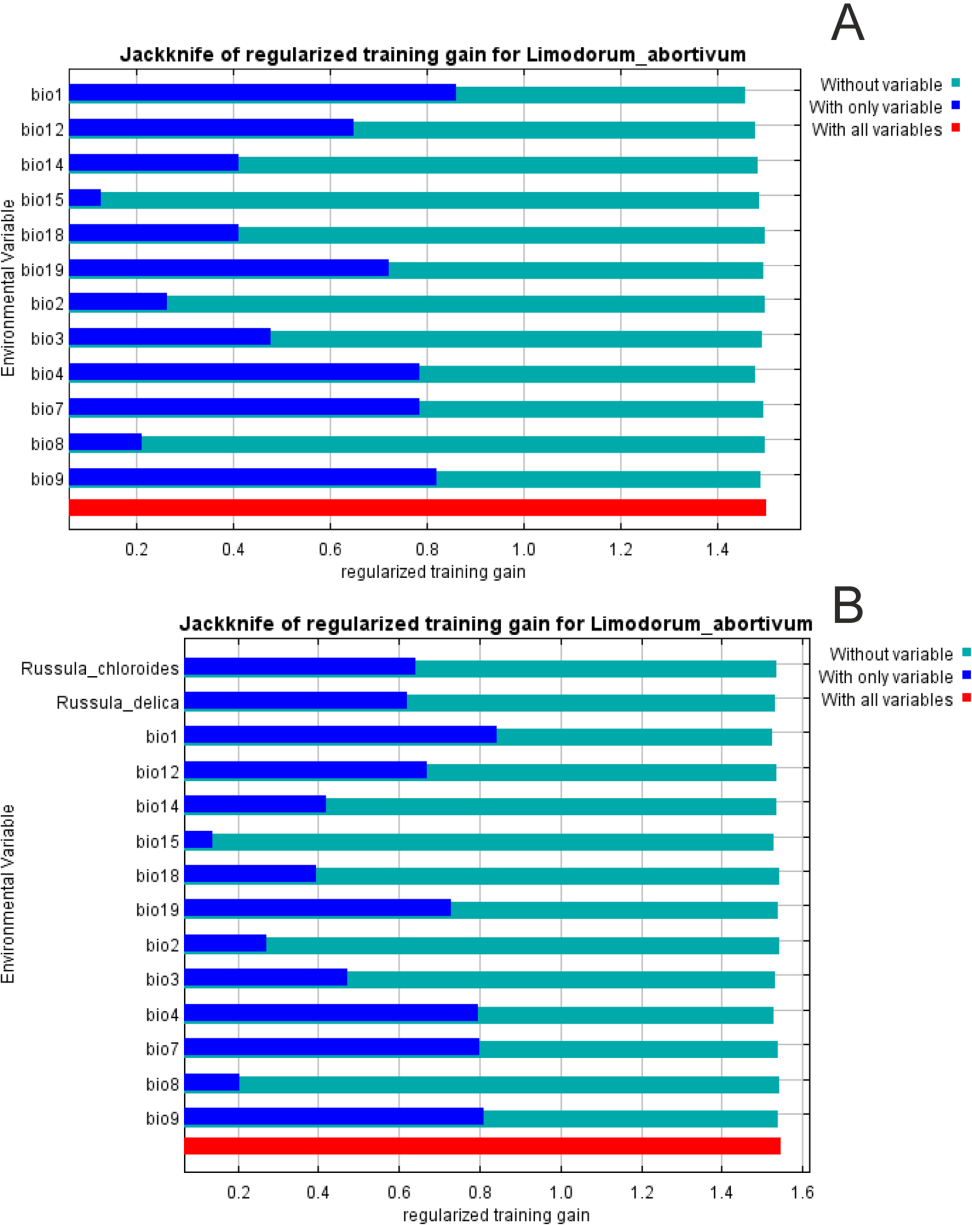
The results of the jackknife test of variable importance. (**A**) bioclims only based models, (**B**) models created based on combined bioclims and fungi data). Values shown are averages over replicate runs.

The results of the jackknife test of variable importance in both sets of models created for *L. abortivum* showed that variable with highest gain when used in isolation is bio1, which therefore appears to have the most useful information by itself. The same variable appears to have the most information that isn't present in the other variables as it reduces the gain the most when it is omitted is bio1 (Fig. 2). Other important bioclimatic factors influencing distribution of *L. abortivum* are bio9, bio4, and bio7. As indicated in the jackknife test, the presence of the two *Russula* species is more important than bio3, bio14, bio2, bio8 and bio15.

### Impact of climate change on orchid symbionts

The future of *Russula chloroides* is uncertain (Table 3, Supplementary Annex 4). While in almost all climate change scenarios projected by CNRM and GISS this fungi will face serious suitable niche loss (13–61%), the INM prediction is more favorable and predicts even 95% expansion of the current fungus range, especially in the northern part of its geographical range.

**Table 3:** Changes in the coverage of suitable niches of *L. abortivum*, its fungal symbionts and pollinator.

Species	Projection	Climate change scenario	Range expansion	Presence in both models	Range contraction	Range change
*Anthophora affinis*	CNRM	SSP1− 2.6	279,518	2,779,019	815,857	− 14.92%
		SSP2− 4.5	192,563.4	2,070,964	1,523,912	− 37.03%
		SSP3− 7.0	250,202.9	1,784,592	1,810,284	− 43.40%
		SSP5− 8.5	155,892.2	1,155,075	2,439,801	− 63.53%
	GISS	SSP1− 2.6	208,082.3	2,933,467	661,408.3	− 12.61%
		SSP2− 4.5	264,008.8	2,519,905	1,074,970	− 22.56%
		SSP3− 7.0	343,036.3	2,374,333	1,220,542	− 24.41%
		SSP5− 8.5	516,552.4	1,988,731	1,606,144	− 30.31%
	INM	SSP1− 2.6	156,570.2	2,877,723	717,152.9	− 15.59%
		SSP2− 4.5	353,081.8	2,522,645	1,072,231	− 20.00%
		SSP3− 7.0	507,263.4	2,024,208	1,570,667	− 29.58%
		SSP5− 8.5	476,558.6	1,748,092	1,846,783	− 38.12%
*Bombus terrestris*	CNRM	SSP1− 2.6	622,823.6	2,204,510.9	409,965.5	+ 8.14%
		SSP2− 4.5	560,580.5	2,117,955.3	496,521.1	+ 2.45%
		SSP3− 7.0	616,961.9	1,879,861.9	734,614.5	− 4.50%
		SSP5− 8.5	320,540.7	1,624,149.9	990,326.5	− 25.62%
	GISS	SSP1− 2.6	232,739.1	1,879,680.1	734,796.3	− 19.20%
		SSP2− 4.5	293,651.7	1,912,140.4	702,336.0	− 15.63%
		SSP3− 7.0	357,476.0	1,661,400.3	953,076.1	− 22.78%
		SSP5− 8.5	579,565.6	1,535,831.1	1,078,645.3	− 19.09%
	INM	SSP1− 2.6	477,841.3	2,211,971.1	402,505.3	+ 2.88%
		SSP2− 4.5	1,060,683.8	2,152,690.5	461,785.9	+ 22.91%
		SSP3− 7.0	1,500,250.3	2,013,134.4	601,342.0	+ 34.38%
		SSP5− 8.5	1,365,375.6	1,666,230.7	948,245.7	+ 15.95%
*Rhodanthidium septemdentatum*	CNRM	SSP1− 2.6	530,540.2	299,808.2	44,081.9	+ 141.46%
		SSP2− 4.5	760,736.5	243,027.1	100,863.0	+ 191.88%
		SSP3− 7.0	912,158.2	253,616.0	90,274.1	+ 239.00%
		SSP5− 8.5	1,310,151.8	225,719.0	118,171.1	+ 346.62%
	GISS	SSP1− 2.6	170,376.9	289,163.9	54,726.2	+ 33.63%
		SSP2− 4.5	278,313.1	310,156.1	33,734.0	+ 71.12%
		SSP3− 7.0	284,091.0	298,375.1	45,515.0	+ 69.38%
		SSP5− 8.5	238,784.8	243,875.0	100,015.1	+ 40.35%
	INM	SSP1− 2.6	201,087.7	289,077.9	54,812.2	+ 42.54%
		SSP2− 4.5	631,617.2	334,429.7	9460.4	+ 180.92%
		SSP3− 7.0	742,313.3	317,899.1	25,991.0	+ 208.30%
		SSP5− 8.5	651,638.1	288,194.8	55,695.3	173.29%
*Limodorum abortivum* (bioclims)	CNRM	SSP1− 2.6	1,246,955	693,793	550,088.1	+ 56.02%
		SSP2− 4.5	978,619.7	482,490.7	761,390.5	+ 17.46%
		SSP3− 7.0	1,318,454	306,845.5	937,035.7	+ 30.66%
		SSP5− 8.5	884,978.1	65,674.99	1,178,206	− 23.57%
	GISS	SSP1− 2.6	728,576.9	668,385	575,496.2	+ 12.31%
		SSP2− 4.5	680,843.9	555,791.1	688,090	− 0.58%
		SSP3− 7.0	787,641.9	335,094.2	908,786.9	− 9.74%
		SSP5− 8.5	772,494.2	111,590.3	1,132,291	− 28.93%
	INM	SSP1− 2.6	886,027.2	845,698.2	398,183	+ 39.22%
		SSP2− 4.5	1,572,576	678,898.3	564,982.9	+ 81.00%
		SSP3− 7.0	1,942,943	408,769.6	835,111.5	+ 89.06%
		SSP5− 8.5	1,851,315	266,499.2	977,381.9	+ 70.26%
*Limodorum abortivum* (bioclims + fungi)	CNRM	SSP1− 2.6	851,992.4	483,900.5	632,816.8	+ 19.63%
		SSP2− 4.5	1,021,670	407,822.2	708,895.1	+ 28.01%
		SSP3− 7.0	1,031,478	203,470.4	913,247	+ 10.59%
		SSP5− 8.5	1,141,051	110,654.2	1,006,063	+ 12.09%
	GISS	SSP1− 2.6	513,868.2	574,625.1	542,092.2	− 2.53%
		SSP2− 4.5	555,482.8	527,938.3	588,779.1	− 2.98%
		SSP3− 7.0	575,675.8	448,638.4	668,078.9	− 8.27%
		SSP5− 8.5	615,150.2	82,391.95	1,034,325	− 37.54%
	INM	SSP1− 2.6	653,807.5	550,928.5	565,788.8	+ 7.88%
		SSP2− 4.5	798,948.5	565,330.1	551,387.2	+ 22.17%
		SSP3− 7.0	1,389,914	372,812.3	743,905	+ 57.85%
		SSP5− 8.5	1,330,285	142,509.1	974,208.2	+ 31.89%
*Russula chloroides*	CNRM	SSP1− 2.6	28,833.18	9262.088	59,616.51	− 44.69%
		SSP2− 4.5	20,173.5	6115.358	62,763.24	− 61.83%
		SSP3− 7.0	52,421.31	7092.678	61,785.92	− 13.60%
		SSP5− 8.5	26,807.45	2685.01	66,193.59	− 57.18%
	GISS	SSP1− 2.6	26,861.33	15,798.01	53,080.59	− 38.07%
		SSP2− 4.5	24,881.25	10,306.76	58,571.84	− 48.91%
		SSP3− 7.0	31,675.34	10,913.65	57,964.94	− 38.17%
		SSP5− 8.5	62,357.64	13,451.24	55,427.35	+ 10.06%
	INM	SSP1− 2.6	57,607.24	19,241.83	49,636.77	+ 11.57%
		SSP2− 4.5	119,863.1	14,768.31	54,110.29	+ 95.46%
		SSP3− 7.0	98,714.54	4414.402	64,464.19	+ 49.73%
		SSP5− 8.5	129,533.8	2564.529	66,314.07	+ 91.78%
*Russula delica*	CNRM	SSP1− 2.6	109,330.4	728,655.5	667,200.4	− 39.97%
		SSP2− 4.5	102,616.3	582,407	813,448.9	− 50.92%
		SSP3− 7.0	78,888.27	602,142.7	793,713.1	− 51.21%
		SSP5− 8.5	88,032.12	354,875.6	1,040,980	− 68.27%
	GISS	SSP1− 2.6	109,883.4	739,569.2	656,286.7	− 39.14%
		SSP2− 4.5	95,549.85	649,878.8	745,977.1	− 46.60%
		SSP3− 7.0	98,376.29	536,237.3	859,618.6	− 54.54%
		SSP5− 8.5	112,648.5	627,051.7	768,804.2	− 47.01%
	INM	SSP1− 2.6	172,404.9	935,860.8	459,995.1	− 20.60%
		SSP2− 4.5	320,886.4	948,731.3	447,124.6	− 9.04%
		SSP3− 7.0	262,300.4	804,372.4	591,483.5	− 23.58%
		SSP5− 8.5	189,722.8	842,703.4	553,152.5	− 26.04%

*Russula delica* will lose suitable niches in all predicted climate change scenarios (Table 3, Supplementary Annex 4). According to CNRM projections the potential range of this fungi will be reduced for 39–68%. Similar result was obtained in GISS projection (39–54%) but in the INM forecast the loss will be lower (9–26%). The most significant niche loss will be observed in southern ad central part of the fungus range.

### Impact of climate change on orchid

**Figure 3:**
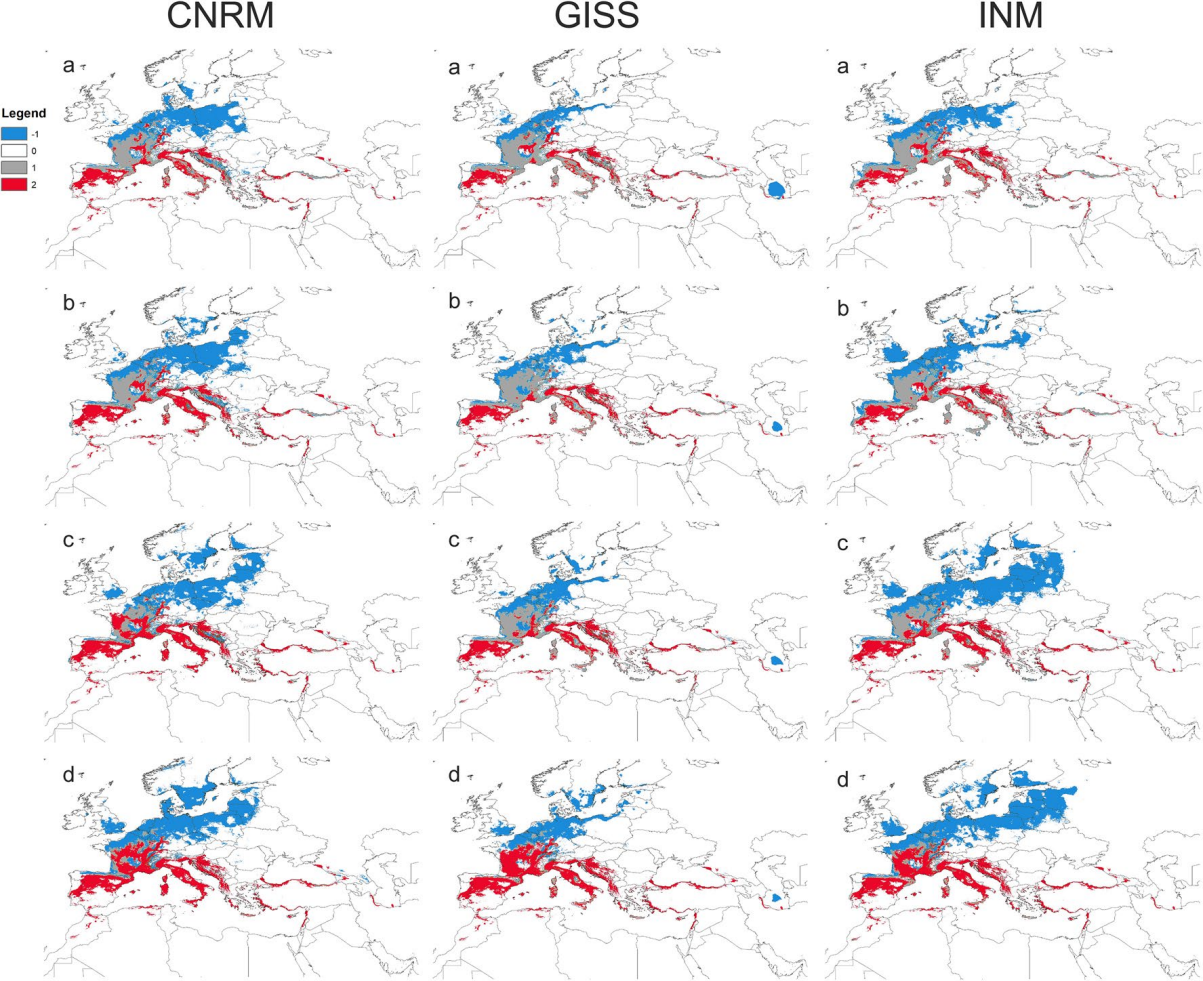
Changes in the orchid distribution according to the combined (bioclims + fungi) models. (**a**) SSP1-2.6 scenario, (**b**) SSP2-4.5 scenario, (**c**) SSP3-7.0 scenario, (**d**) SSP5-8.5 scenario. Legend: -1—range expansion, 0—no occurrence, 1—present, 2—range contraction. Maps generated by the author in ArcGIS^47,48^.

According to models created based on bioclimatic data only, the potential range of *L. abortivum* will expand in most predicted climate change scenarios except of SSP5-8.5 (CNRM and GISS projections) and SSP3-7.0 (GISS projection) (Table 3, Supplementary Annex 5). However, due to the predicted loss of suitable niches by its fungal symbionts, the models based on both bioclimatic data and distribution models of fungi, the extent of range expansion will be lower in all climate change scenarios. Moreover, according to the GISS projections the orchid will lose 3–38% of currently suitable niches (Table 3). The most significant niche loss will be observed in the southern part of the orchid range and expansion to the north and north-east direction is predicted (Fig. 3).

### Impact of climate change on orchid pollinators

**Figure 4:**
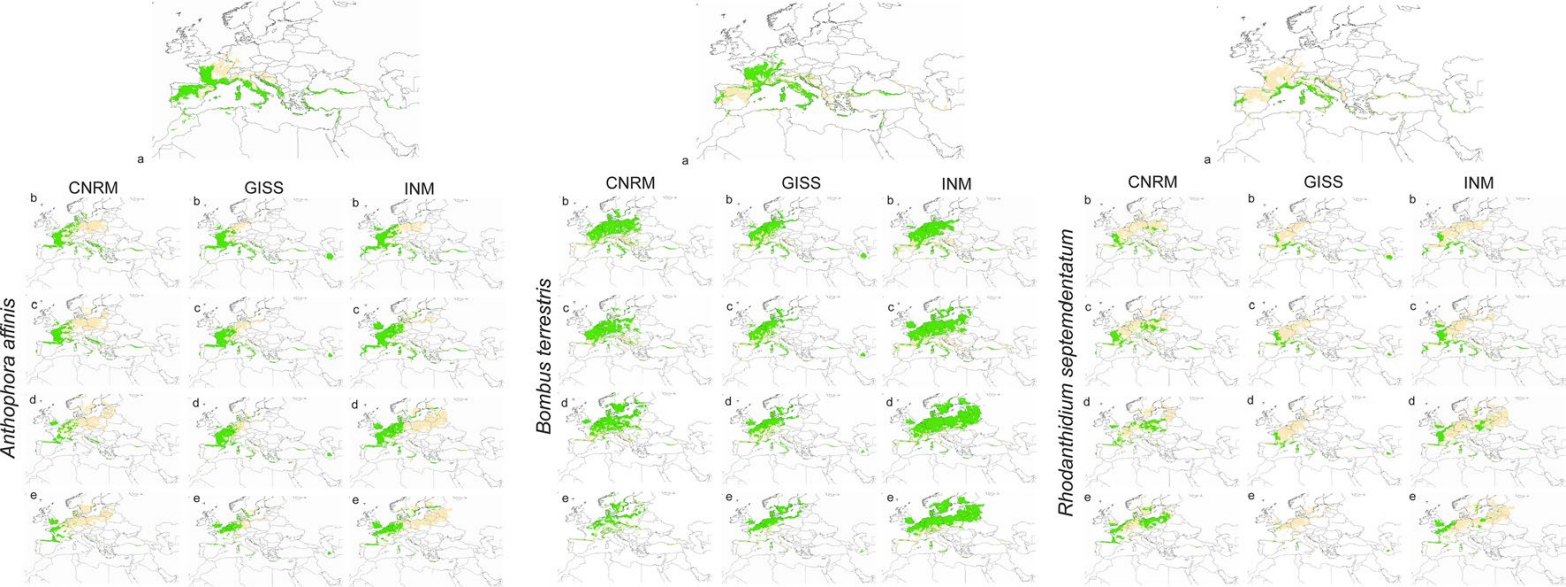
Presence of pollinator within *L. abortivum* range*.* Areas of overlap between pollinator and orchid range marked with green, areas suitable only for an orchid occurrence marked in sand yellow. (**a**) present time, (**b**) SSP1-2.6 scenario, (**c**) SSP2-4.5 scenario, (**d**) SSP3-7.0 scenario, (**e**) SSP5-8.5 scenario. Maps generated by the author in ArcGIS^47,48^.

As mentioned before, models of *Anthophora affinis* and *Bombus terrestris* received lower scores of TSS than expected (but relatively high AUC scores), however, the models of these insects created for the present time are consistent with the known geographical ranges of species studies and should be discussed as reliable ones. *Anthophora affinis* will lose suitable niches in the southern part of its geographical range according to all conducted analyses (Table 3, Supplementary Annex 6). The expansion of current range is expected to occur mostly in Central Europe. In CNRM forecast the range will contract by 14–63%, in GISS 12–30% and in INM by 15–38%. The currently observed overlap of the potential range of *Anthophora affinis* and *L. abortivum* is about 67% but in almost all predictions it will decrease as a result of climate change (Table 4, Fig. 4).

**Table 4:** Availability of pollinator in *L. abortivum* geographical range.

Species	Projection	Climate change scenario	Overlap of orchid and pollinator range
*Anthophora affinis*	Present time		67.29%
	CNRM	SSP1-2.6	48.22%
		SSP2-4.5	37.31%
		SSP3-7.0	25.92%
		SSP5-8.5	21.99%
	GISS	SSP1-2.6	67.14%
		SSP2-4.5	60.12%
		SSP3-7.0	65.27%
		SSP5-8.5	56.41%
	INM	SSP1-2.6	59.29%
		SSP2-4.5	71.14%
		SSP3-7.0	43.43%
		SSP5-8.5	36.35%
*Bombus terrestris*	Present time		54.96%
	CNRM	SSP1-2.6	73.87%
		SSP2-4.5	76.29%
		SSP3-7.0	78.02%
		SSP5-8.5	70.70%
	GISS	SSP1-2.6	69.90%
		SSP2-4.5	74.16%
		SSP3-7.0	72.93%
		SSP5-8.5	84.43%
	INM	SSP1-2.6	70.57%
		SSP2-4.5	76.64%
		SSP3-7.0	86.55%
		SSP5-8.5	84.35%
*Rhodanthidium septemdentatum*	Present time		22.98%
	CNRM	SSP1-2.6	22.79%
		SSP2-4.5	33.28%
		SSP3-7.0	33.49%
		SSP5-8.5	49.29%
	GISS	SSP1-2.6	22.38%
		SSP2-4.5	23.28%
		SSP3-7.0	18.24%
		SSP5-8.5	6.51%
	INM	SSP1-2.6	20.17%
		SSP2-4.5	32.29%
		SSP3-7.0	23.59%
		SSP5-8.5	20.88%

The future of *Bombus terrestris* is uncertain. According to CNRM and INM projections the climate changes will be generally favorable for this pollinator which will expand its geographical range in almost all analysed climate change scenarios (Table 3, Supplementary Annex 6), except of CNRM SSP5-8.5 and SSP3-7.0 predictions On the other hand, the GISS projection predicts the significant loss (19–22%) of suitable niches in all scenarios of global warming (Table 3). Overall, the contraction is expected to occur in south and south-eastern part of the insect geographical range. The new niches should be available for the bumblebee in northern and north-western edges of the current range. The overlap of orchid and *B. terrestris* ranges will be much higher than currently observed (Table 4, Fig. 4) and this pollinator will be available for almost all populations of *L. abortivum.*

Climate change will be very favorable for *Rhodanthidium septemdentatum* according to CNRM and INM projections (except of INM SSP1-2.6 scenario). In these predictions the insect will much expand its potential range (Table 3, Supplementary Annex 6). However, as the analyses conducted using GISS projections indicated future loss of suitable niches of orchid pollinator (33–71%). This bee is currently available for approximately 23% of orchid populations. The overlap of orchid and *R. septemdentatum* potential ranges will be similar to or higher than currently observed in almost all CNRM and INM projections. On the contrary, the availability of pollen vector will be reduced in GISS simulations (Table 4, Fig. 4).

## Discussion

### Importance of ecological factors in species distribution models

This study showed the importance of ecological factor in species distribution models. Even though according to the jackknife test, the geographical range of *L. abortivum* is shaped mainly by climatic factors, the incorporation of symbiotic fungi models in the analyses significantly modified the predicted distribution of orchid under future climate change.

While all Orchidaceae require mycorrhizal symbioses during the seed germination as their seeds lack endosperms and contain limited storage reserves^25^, some species were found to keep receiving carbon from mycorrhizal fungi even after initiation of photosynthesis^70,71^. As indicated in previous research^72^ the photosynthetic rates of the Violet Limodore are very low and the carbon assimilation activity is insufficient to support the energy requirements of adult plants.

So far little is known on the impact of global warming on plant endophytes, primarily because most symbionts remain unidentified^73^. While molecular studies allowed to isolate and genetically characterize fungi from orchid roots, leaves and stems^74–77^, the actual composition of orchid fungal symbionts is still poorly explored and most of taxa found in plant tissues have been assigned only to family or genus rank^78–81^. The lack of species-level identification of orchid symbionts excludes possibility of any reliable broad-scale analyses of the importance of fungi on the Orchidaceae distribution.

As any research aimed at estimating possible impact of climate change on complex relationships between plant, fungi and insects, this study has some limitations. So far the analyses of *L. abortivum* symbionts were geographically limited and further investigations are required to evaluate the actual variation of fungal symbionts of this orchid. It is possible that not only Russulaceae can compensate insufficient plant CO_2_ fixation. Moreover, Girlanda et al.^35^ identified three species of Russulaceae as symbionts of *L. abortivum.* Two of them were included in ENM analyses for this study. The third one, *Russula brevipes* is widespread in North America and only recently was reported outside its native range. It is not sure if this fungus will actually spread in Eurasia to serve as a long-term symbiont for *L. abortivum.* Also, due to the small number of records of this fungus in the non-native areas, at this point it is not possible to evaluate its current or future potential range in Eurasia.

Noteworthy, *Russula* representatives are ectomycorrhizal fungi and their occurrence is strictly related with the presence of host plants. *R. delica* seems to have broad spectrum of potential hosts and it was reported growing with both conifers (*Pseudotsuga menziesii*) and broadleaved trees (*Corylus, Fagus, Quercus, Tilia*)^82^. On the other hand, *R. chloroides* favours habitats with oak trees, although it was sometimes reported growing under other broadleaf trees (*Carpinus, Crataegus*) and conifer trees (*Abies alba*)^83,84^.

### Uncertainty of climate models

As global warming became one of the most important threats to biodiversity and ecosystems functioning, numerous mathematical models of future climate changes scenarios has been produced to describe possible impact of human on Earth temperature and precipitations^85^. Clearly, the usefulness of any climate model is tested by the conformity of its output to a given set of known conditions. For that reason, the actual trustworthiness of any models presenting future climate is impossible to assess. In this study three different simulations of climatic conditions were used to produce the most reliable prediction of changes in the distribution of suitable niches of species studied.

Noteworthy, earlier models created for *L. abortivum*^86^ based on formerly recognized climate change scenarios (A1b, A2a and B2a) indicated loss of orchid suitable niches. In the present study habitat loss was predicted only in GISS simulation of future climate change while both CNRM and INM projections forecasted expansion of orchid geographical range. While most areas currently occupied by the orchid will not be suitable for this species in the future, all created models predicted migration of *L. abortivum* to the higher latitudes and loss of suitable niches in the southern part of species geographical range. The same scheme of poleward range shift as a response to the global warming was already predicted to occur in other plant^87^ and animal^88,89^ species. Evans and Jacquemyn^90^ suggested that terrestrial Orchidaceae with a wide distribution will be more capable of shifting their distributions under global warming than species with a restricted geographical range. While obviously species with broader environmental tolerance have higher survival chances than more specialized taxa, the fragility of ecological interactions can further affect persistence of widely distributed organisms. Studies on future distribution of European orchids are rather scarce and while some taxa (*Nigritella nigra*^91^, *Pseudorchis albida*^92^) are predicted to lose their suitable niches across their ranges, others are expected to experience a poleward range shift (some *Orchis*^93^, *Ophrys insectifera*^94^, some *Epipactis*^90^). Noteworthy, none of the previous research considered the importance of mycorrhizal fungi on Orchidaceae distribution and most of the published analyses ignored also the future availability of orchid pollen vectors.

While it is generally accepted that climatic factors play the primary role in shaping biodiversity at broad scales, there are numerous other abiotic variables which can affect species occurrence. Scientists still explore the importance of topography^95^, physico-chemical properties of soil^96–98^, and the substrate thickness^99^ on the plant distribution^100^. Unfortunately, at this moment there are no tools which could be used to predict changes of these variables in response to climate change and hence these factors cannot be included in the niche modelling.

Another obstacle in broad scale modelling of species distribution is lack of data and unequal sampling throughout the geographical range of the species. The availability of precise location data remains limited, especially when dealing with rare or poorly recognized species^49,101,102^. Moreover, species records are often constrained by the variety of their sources and spatial biases caused by unequal sampling efforts^103^ and by uneven field accessibility^104^. The adequacy of sample bias correction methods remains uncertain^105^ and field validation is still considered to be the best standard practice to assess models’ reliability^106^. Unfortunately, field validation is sometimes impossible to use, especially in geographically extensive scale studies^105^. In this study some geographical regions also seemed to be inadequately sampled and for that reason the spatial filtering on various scales of topographic heterogeneity was conducted to reduce sampling bias. The general consistence of the models created for the present time with the known geographical range of studied species suggest reliability of conducted analyses, also for species which received lower scores of TSS tests (but relatively high AUC test scores).

### Future availability of pollinators

Flowers of the Violet Limodore are most often cleistogamous (pollination takes place inside the flower bud), but insect pollination is more beneficial for the long-term survival of *L. abortivum* populations by increasing their genetic diversity. So far very few studies on the impact of global warming on orchid distribution incorporated also the analyses of future availability of pollen vectors for plant populations^107–109^. This study indicated that the climate change can be favorable for orchid not only by direct expansion of the niches suitable for the plant occurrence, but also in increasing pollinators availability as a result of insects range shifts.

In this study two species of solitary bees were included in the analyses. According to created models *A. affinis* will lose numerous suitable niches while *R. septemdentatum* will expand its geographical range. However, as indicated in previous studies^110^, for solitary bees, responses to climate change will be related to biological processes occurring prior to emergence. Among possible consequences of global warming accelerated development^111^, reduced energy reserves^112,113^, increased mortality^114^, advanced emergence^115^, and reduced post-emergence lifespan^116^ are expected to occur.

While according to available data the main flight period of both studied bee species, *A. biciliata* and *R. septemdentatum*, is rather short and is susceptible for climate change, the buff-tailed bumblebee is relatively resistant to climate warming. According to the published study results^117^ advances in mid-date of the main flight period over the 35-year period was 13 days for *B. terrestris* and the duration of the main flight period was reduced by about 7 days. The models presented in this study do not consider possibility of incompatibility of orchid flowering time and its pollinator phenology. This dangerous phenomena which can disturb pollen transfer and plant reproductive success was already detected in European and Australian orchids^118,119^. The possible mis-match of orchid flowering time and insect activity period can further limit the possibility of cross-pollination of *L. abortivum.*

## Conclusions

To conclude, the incorporation of ecological relationships, e.g. fungal symbionts, pollen vectors, is crucial to produce more accurate distribution models of plant species. Moreover, due to the discrepancies between projections of future climatic conditions, various predictions and climate change scenarios should be analysed to uncover all possible changes in the studied species potential geographical range. The maps presented in this study can be useful for establishing conservation actions on *L. abortivum*. The priority in creating new conservation areas should be given to the regions which will be suitable for the occurrence of the orchid, its symbionts and pollinators in the future. Without doubt, more effort should be made to identify orchid symbionts and incorporate geographical distribution records of fungi into public databases to allow more efficient geographical analyses of plant-fungi relationships.

## Supplementary Information

Supplementary Information 1.

Supplementary Information 2.

Supplementary Information 3.

Supplementary Information 4.

Supplementary Information 5.

Supplementary Information 6.

## Data Availability

All relevant data are presented in the manuscript and supplementary files.
