# Supplementary material for: Loss of fungal symbionts and changes in pollinator availability caused by climate change will affect the distribution and survival chances of myco-heterotrophic orchid species
Source: Sci Rep. 2023 Apr 26;13:6848. doi: 10.1038/s41598-023-33856-y (PMC10133392; doi:10.1038/s41598-023-33856-y)
Supplement: Supplementary file 1 — Supplementary Information 1. [file 41598_2023_33856_MOESM1_ESM.pdf]

Loss of fungal symbionts and changes in pollinator availability caused by climate change will affect the distribution and survival chances of myco-heterotrophic orchid species

Marta Kolanowska[1]

[1] University of Lodz, Faculty of Biology and Environmental Protection, Department of Geobotany and Plant Ecology, Banacha 12/16, 90-237 Lodz, Poland (martakolanowska@wp.pl, ORCID: 0000-0001-5347-5403)

Supplementary Annex 1. Raw GBIF datasets of studied species.

**Species**	**GBIF dataset**
Limodorum abortivum	https://doi.org/10.15468/dl.j4y8f4
Anthophora biciliata	https://doi.org/10.15468/dl.jrjany
Bombus terrestris	https://doi.org/10.15468/dl.r9upj5
Rhodanthidium septemdentatum	https://doi.org/10.15468/dl.apxupg
Russula delica	https://doi.org/10.15468/dl.yf76jk
Russula chloroides	https://doi.org/10.15468/dl.qhb46y
