# Supplementary material for: Loss of fungal symbionts and changes in pollinator availability caused by climate change will affect the distribution and survival chances of myco-heterotrophic orchid species
Source: Sci Rep. 2023 Apr 26;13:6848. doi: 10.1038/s41598-023-33856-y (PMC10133392; doi:10.1038/s41598-023-33856-y)
Supplement: Supplementary file 2 — Supplementary Information 2. [file 41598_2023_33856_MOESM2_ESM.pdf]

Loss of fungal symbionts and changes in pollinator availability caused by climate change will affect the distribution and survival chances of myco-heterotrophic orchid species

Marta Kolanowska[1]

[1] University of Lodz, Faculty of Biology and Environmental Protection, Department of Geobotany and Plant Ecology, Banacha 12/16, 90-237 Lodz, Poland (martakolanowska@wp.pl, ORCID: 0000-0001-5347-5403)

Supplementary Annex 2. Localities of studies species used in ENM analyses.

**Species**	**Decimal Latitude**	**Decimal Longitude**
Anthophora affinis	46,17201	7,42188
Anthophora affinis	37,7111	-2,16593
Anthophora affinis	38,27303	16,14861
Anthophora affinis	36,16781	51,3113
Anthophora affinis	44,18895	3,13172
Anthophora affinis	44,05379	6,23946
Anthophora affinis	43,82695	5,31474
Anthophora affinis	32,730556	35,005556
Anthophora affinis	44,192533	4,14741
Anthophora affinis	36,76306	3,05056
Anthophora affinis	37,66866	-1,69411
Anthophora affinis	41,63121	-4,52142
Anthophora affinis	43,6231	6,7519
Anthophora affinis	44,35479	4,45564
Anthophora affinis	30,07708	31,28591
Anthophora affinis	41,5262	-5,39526
Anthophora affinis	41,6486	-4,7514
Anthophora affinis	37,393056	25,268056
Anthophora affinis	49,15	1,666667
Bombus terrestris	46,050411	7,480038
Bombus terrestris	46,659275	11,184875
Bombus terrestris	37,107796	-3,385179
Bombus terrestris	45,210753	6,553425
Bombus terrestris	46,177828	8,810753
Bombus terrestris	47,55673	13,166979
Bombus terrestris	40,246123	22,088871
Bombus terrestris	40,150275	22,437687
Bombus terrestris	46,233791	9,85195
Bombus terrestris	36,980048	-3,118275
Bombus terrestris	42,547311	2,390783
Bombus terrestris	41,76162	9,22858
Bombus terrestris	47,624413	13,474831
Bombus terrestris	42,068322	9,134928
Bombus terrestris	47,42819	12,84305
Bombus terrestris	36,797363	-3,911321
Bombus terrestris	40,899475	-8,235963
Bombus terrestris	42,4943	8,99149
Bombus terrestris	42,96878	0,00126
Bombus terrestris	43,0281	12,7001
Bombus terrestris	34,309345	35,986144
Bombus terrestris	42,758271	9,402843
Bombus terrestris	45,073343	7,348734
Bombus terrestris	45,81552	10,975498

Bombus terrestris	36,707785	27,520673
Bombus terrestris	37,988398	22,462394
Bombus terrestris	37,769154	26,664192
Bombus terrestris	39,94339	20,664755
Bombus terrestris	44,092625	6,861743
Bombus terrestris	45,980251	11,302949
Bombus terrestris	46,094402	6,723174
Bombus terrestris	46,21936	7,945729
Bombus terrestris	46,483891	9,191854
Bombus terrestris	46,968395	8,892267
Bombus terrestris	46,994932	11,264084
Bombus terrestris	47,308528	12,37568
Bombus terrestris	49,18362	19,072199
Bombus terrestris	46,3006	13,8862
Bombus terrestris	40,582177	21,414416
Bombus terrestris	41,819464	2,355795
Bombus terrestris	46,818085	9,294777
Bombus terrestris	56,666775	-5,076081
Bombus terrestris	42,86964	1,37632
Bombus terrestris	37,099524	-3,55471
Bombus terrestris	47,16	12,96
Bombus terrestris	47,18	13,63
Bombus terrestris	59,559151	6,466055
Bombus terrestris	61,074193	6,581103
Bombus terrestris	46,874204	10,890793
Bombus terrestris	42,479918	2,947167
Bombus terrestris	38,737795	22,339217
Bombus terrestris	43,201672	17,126312
Bombus terrestris	44,148758	7,730237
Bombus terrestris	46,673088	13,936028
Bombus terrestris	46,999748	8,528824
Bombus terrestris	49,120441	20,059088
Bombus terrestris	59,990652	6,012667
Bombus terrestris	47,4	13,25
Bombus terrestris	42,433542	-7,03013
Bombus terrestris	46,78	12,66
Bombus terrestris	47,01	12,62
Bombus terrestris	59,574372	8,559418
Bombus terrestris	62,593265	9,66404
Bombus terrestris	46,75	12,47
Bombus terrestris	47,2667	11,3833
Bombus terrestris	51,994233	-9,522113
Bombus terrestris	59,67706	11,282352
Bombus terrestris	55,58185	10,601719
Bombus terrestris	55,928755	12,522908
Bombus terrestris	50,932205	0,758162

Bombus terrestris	51,220135	0,29001
Bombus terrestris	51,279228	-1,965919
Bombus terrestris	51,372387	-2,441766
Bombus terrestris	51,388341	-2,817584
Bombus terrestris	51,472538	-0,320357
Bombus terrestris	51,613642	-4,912387
Bombus terrestris	51,87764	1,004257
Bombus terrestris	52,310448	0,29106
Bombus terrestris	52,493797	1,756712
Bombus terrestris	52,694229	-2,752951
Bombus terrestris	52,986314	-3,823095
Bombus terrestris	53,312298	-1,577087
Bombus terrestris	54,296158	-2,793715
Bombus terrestris	54,421753	-2,419179
Bombus terrestris	55,657303	-3,479321
Bombus terrestris	55,88498	-3,266791
Bombus terrestris	57,183193	-6,057586
Bombus terrestris	40,532635	-3,671329
Bombus terrestris	40,618641	8,196608
Bombus terrestris	47,1613	-1,14386
Bombus terrestris	47,69294	-2,57718
Bombus terrestris	49,20705	2,48297
Bombus terrestris	51,55409	0,801404
Bombus terrestris	51,787645	5,921023
Bombus terrestris	51,911151	-2,083004
Bombus terrestris	52,490582	-2,097724
Bombus terrestris	53,254617	-1,905683
Bombus terrestris	53,333361	-2,828676
Bombus terrestris	53,553521	9,97311
Bombus terrestris	55,56595	13,71328
Bombus terrestris	56,77436	16,57265
Bombus terrestris	57,23108	16,50259
Bombus terrestris	57,522379	-1,994088
Bombus terrestris	57,989518	7,061252
Bombus terrestris	58,010926	7,556844
Bombus terrestris	58,10161	6,58528
Bombus terrestris	58,33382	8,461977
Bombus terrestris	58,57672	13,41955
Bombus terrestris	58,664406	5,648202
Bombus terrestris	58,834514	23,044167
Bombus terrestris	59,098463	10,841156
Bombus terrestris	59,345778	10,745594
Bombus terrestris	59,396673	24,625035
Bombus terrestris	59,5208	18,06321
Bombus terrestris	59,75119	17,75949
Bombus terrestris	59,866259	10,8201

Bombus terrestris	60,00262	15,81931
Bombus terrestris	60,097888	10,877556
Bombus terrestris	60,111608	23,532027
Bombus terrestris	60,187939	24,812679
Bombus terrestris	60,384779	25,659037
Bombus terrestris	60,411235	22,268794
Bombus terrestris	60,500488	26,448362
Bombus terrestris	60,709	28,738
Bombus terrestris	60,834436	11,064379
Bombus terrestris	61,22543	26,050269
Bombus terrestris	61,411074	12,110781
Bombus terrestris	61,868053	28,90423
Bombus terrestris	63,345963	10,327697
Bombus terrestris	63,74521	11,31394
Bombus terrestris	63,874606	9,853351
Bombus terrestris	59,27984	15,16201
Bombus terrestris	35,892522	-5,365625
Bombus terrestris	36,398133	6,665488
Bombus terrestris	37,860599	24,031325
Bombus terrestris	38,802909	20,7269
Bombus terrestris	39,191126	26,44678
Bombus terrestris	39,193098	23,32943
Bombus terrestris	40,564274	-8,762104
Bombus terrestris	41,098099	-8,384972
Bombus terrestris	41,131667	24,169269
Bombus terrestris	41,39	2,11
Bombus terrestris	41,590281	-8,803906
Bombus terrestris	41,622981	2,438116
Bombus terrestris	43,039781	1,134772
Bombus terrestris	43,640193	3,403044
Bombus terrestris	47,17122	-0,892784
Bombus terrestris	49,538926	8,546337
Bombus terrestris	50,063544	14,599726
Bombus terrestris	50,417573	30,55763
Bombus terrestris	50,781322	0,095922
Bombus terrestris	50,783084	-3,330266
Bombus terrestris	51,018863	3,758886
Bombus terrestris	51,288679	-1,078281
Bombus terrestris	51,70327	19,440038
Bombus terrestris	52,252768	35,298651
Bombus terrestris	52,265544	10,535661
Bombus terrestris	52,45133	0,914322
Bombus terrestris	53,18798	-4,463481
Bombus terrestris	53,553006	-2,299104
Bombus terrestris	53,957456	27,60394
Bombus terrestris	54,560619	39,690065

Bombus terrestris	54,60776	-3,367258
Bombus terrestris	54,744169	-3,056647
Bombus terrestris	55,819106	39,009455
Bombus terrestris	56,193854	-3,605826
Bombus terrestris	56,333312	43,938232
Bombus terrestris	56,870774	35,890563
Bombus terrestris	57,812114	24,345309
Bombus terrestris	58,657756	37,276863
Bombus terrestris	55,535772	11,514447
Bombus terrestris	38,747693	-9,193268
Bombus terrestris	39,495978	-8,806885
Bombus terrestris	39,702454	-8,288713
Bombus terrestris	39,910963	-1,95123
Bombus terrestris	41,57705	9,33687
Bombus terrestris	41,963856	2,82876
Bombus terrestris	43,642819	11,072901
Bombus terrestris	44,271405	9,393295
Bombus terrestris	49,494934	5,512697
Bombus terrestris	49,859128	6,093714
Bombus terrestris	51,619164	6,191453
Bombus terrestris	51,74385	-0,062422
Bombus terrestris	51,989125	7,633803
Bombus terrestris	52,066565	1,475658
Bombus terrestris	52,652597	-1,53179
Bombus terrestris	53,187035	6,51275
Bombus terrestris	53,837605	-2,479982
Bombus terrestris	53,893158	-1,531855
Bombus terrestris	55,06892	-1,484533
Bombus terrestris	55,306403	10,755366
Bombus terrestris	55,695464	12,205358
Bombus terrestris	56,056508	-2,651933
Bombus terrestris	56,4007	15,727237
Bombus terrestris	33,180333	35,771138
Bombus terrestris	36,537537	32,066242
Bombus terrestris	36,86002	28,228297
Bombus terrestris	37,563575	-1,281255
Bombus terrestris	38,570142	-8,907019
Bombus terrestris	39,008308	-8,963977
Bombus terrestris	40,418883	17,209659
Bombus terrestris	41,084306	16,295837
Bombus terrestris	41,879397	8,93283
Bombus terrestris	42,846278	11,543985
Bombus terrestris	43,23788	-8,383095
Bombus terrestris	43,936167	4,9522
Bombus terrestris	44,898979	9,261808
Bombus terrestris	45,481736	10,983681

Bombus terrestris	47,96236	2,498815
Bombus terrestris	48,010539	16,301969
Bombus terrestris	48,585748	40,352601
Bombus terrestris	48,611755	8,843567
Bombus terrestris	49,199657	43,984221
Bombus terrestris	49,488122	7,248738
Bombus terrestris	50,08707	14,297978
Bombus terrestris	50,17258	8,626955
Bombus terrestris	50,3749	-4,139372
Bombus terrestris	50,811797	-1,66232
Bombus terrestris	50,864621	1,708328
Bombus terrestris	50,894138	34,8302
Bombus terrestris	50,990938	-4,530825
Bombus terrestris	51,307178	1,1247
Bombus terrestris	51,37742	10,092292
Bombus terrestris	51,484512	7,072328
Bombus terrestris	51,501025	0,033506
Bombus terrestris	51,62627	-3,920183
Bombus terrestris	51,686235	8,701322
Bombus terrestris	51,739083	-1,24105
Bombus terrestris	51,818642	-0,646178
Bombus terrestris	51,946472	8,785106
Bombus terrestris	51,998448	6,935311
Bombus terrestris	52,08827	4,41408
Bombus terrestris	52,161817	-0,471167
Bombus terrestris	52,207887	8,45635
Bombus terrestris	52,415403	7,846667
Bombus terrestris	52,624529	1,501549
Bombus terrestris	52,847247	7,332638
Bombus terrestris	52,981315	-6,031989
Bombus terrestris	53,230399	10,392973
Bombus terrestris	53,422762	50,009725
Bombus terrestris	53,705543	10,362846
Bombus terrestris	53,741763	-2,961197
Bombus terrestris	53,961187	-1,088161
Bombus terrestris	54,409414	9,238302
Bombus terrestris	54,447112	-5,829022
Bombus terrestris	54,492706	-0,644885
Bombus terrestris	54,664982	-2,744353
Bombus terrestris	54,84987	-1,902555
Bombus terrestris	55,515539	28,700499
Bombus terrestris	55,754802	37,614485
Bombus terrestris	56,121351	9,890363
Bombus terrestris	56,338283	-2,783372
Bombus terrestris	57,305695	-2,801487
Bombus terrestris	57,89085	26,646236

Bombus terrestris	58,343461	26,828622
Bombus terrestris	58,615799	23,313104
Bombus terrestris	59,50215	13,32037
Bombus terrestris	63,813103	20,345162
Bombus terrestris	36,107589	-5,82233
Bombus terrestris	37,156723	-6,875113
Bombus terrestris	39,635629	2,616675
Bombus terrestris	40,65447	-8,141811
Bombus terrestris	41,129353	1,308155
Bombus terrestris	42,235439	8,582851
Bombus terrestris	42,408672	-1,593624
Bombus terrestris	42,759997	10,285187
Bombus terrestris	42,91903	-2,03945
Bombus terrestris	43,290081	-2,122316
Bombus terrestris	43,551739	6,935868
Bombus terrestris	43,70198	7,21085
Bombus terrestris	43,786103	1,485772
Bombus terrestris	43,914526	2,934876
Bombus terrestris	44,03652	1,220812
Bombus terrestris	44,342797	4,150228
Bombus terrestris	45,183106	-1,08503
Bombus terrestris	46,246492	-1,498702
Bombus terrestris	47,437054	9,693718
Bombus terrestris	47,541363	7,6661
Bombus terrestris	47,724727	13,089458
Bombus terrestris	48,046214	12,830985
Bombus terrestris	48,104758	17,035558
Bombus terrestris	48,144362	14,005938
Bombus terrestris	48,161167	11,635255
Bombus terrestris	48,275232	16,345638
Bombus terrestris	48,461338	-0,062864
Bombus terrestris	48,469658	7,93567
Bombus terrestris	48,867973	2,780408
Bombus terrestris	49,5074	0,16352
Bombus terrestris	49,5708	8,313416
Bombus terrestris	49,794567	9,26753
Bombus terrestris	49,875926	8,679042
Bombus terrestris	49,930502	7,974476
Bombus terrestris	49,979731	17,78806
Bombus terrestris	50,400162	7,266755
Bombus terrestris	50,660893	6,336933
Bombus terrestris	50,76841	3,65505
Bombus terrestris	50,779178	-1,08325
Bombus terrestris	50,82049	2,91428
Bombus terrestris	50,838177	15,631773
Bombus terrestris	50,916142	-1,402867

Bombus terrestris	50,930112	4,323118
Bombus terrestris	50,94016	4,02085
Bombus terrestris	51,114262	8,329148
Bombus terrestris	51,134342	1,353928
Bombus terrestris	51,175125	6,431726
Bombus terrestris	51,377883	6,743545
Bombus terrestris	51,382867	-1,30865
Bombus terrestris	51,47183	4,80224
Bombus terrestris	51,527797	45,948217
Bombus terrestris	51,764592	7,398522
Bombus terrestris	51,783087	0,257453
Bombus terrestris	51,982593	8,03589
Bombus terrestris	51,990117	-1,743455
Bombus terrestris	52,13406	-4,439988
Bombus terrestris	52,249475	10,971495
Bombus terrestris	52,504483	9,918306
Bombus terrestris	53,016186	7,791198
Bombus terrestris	53,657965	-1,795524
Bombus terrestris	54,296735	10,105121
Bombus terrestris	54,314658	10,896289
Bombus terrestris	54,380806	9,007865
Bombus terrestris	55,066917	26,02385
Bombus terrestris	55,4072	12,9979
Bombus terrestris	55,440833	-2,751944
Bombus terrestris	55,70771	13,23237
Bombus terrestris	55,738919	24,322761
Bombus terrestris	55,77209	14,08298
Bombus terrestris	55,799355	-3,93872
Bombus terrestris	56,050758	-4,513055
Bombus terrestris	56,27435	13,26073
Bombus terrestris	56,42413	16,4123
Bombus terrestris	56,529667	9,55465
Bombus terrestris	56,88061	14,80135
Bombus terrestris	56,96609	18,26247
Bombus terrestris	57,0345	16,82891
Bombus terrestris	57,12646	12,25253
Bombus terrestris	57,176955	-2,089603
Bombus terrestris	57,387192	10,120086
Bombus terrestris	57,61513	18,3355
Bombus terrestris	57,64866	11,77427
Bombus terrestris	57,69994	12,05278
Bombus terrestris	57,74987	14,14918
Bombus terrestris	57,81998	13,14914
Bombus terrestris	58,14547	24,932082
Bombus terrestris	58,236566	26,534519
Bombus terrestris	58,2913	15,24161

Bombus terrestris	58,32987	15,73021
Bombus terrestris	58,33137	14,82315
Bombus terrestris	58,35273	12,35974
Bombus terrestris	58,41663	13,86643
Bombus terrestris	58,616278	7,420731
Bombus terrestris	58,868204	9,39936
Bombus terrestris	58,87735	14,90275
Bombus terrestris	58,923812	5,877893
Bombus terrestris	58,997955	9,739957
Bombus terrestris	59,056561	10,1774
Bombus terrestris	59,062187	10,402536
Bombus terrestris	59,137143	11,355334
Bombus terrestris	59,176369	11,660043
Bombus terrestris	59,233586	9,626589
Bombus terrestris	59,38759	17,00486
Bombus terrestris	59,394363	15,84843
Bombus terrestris	59,415566	5,266176
Bombus terrestris	59,46867	17,732
Bombus terrestris	59,51137	17,39907
Bombus terrestris	59,59717	12,95919
Bombus terrestris	59,59896	16,4522
Bombus terrestris	59,62569	16,89324
Bombus terrestris	59,736438	10,410962
Bombus terrestris	59,766549	9,995662
Bombus terrestris	59,824828	30,329113
Bombus terrestris	59,85443	18,19662
Bombus terrestris	59,87067	16,92326
Bombus terrestris	59,92636	11,490045
Bombus terrestris	60,155147	5,584745
Bombus terrestris	60,326669	5,348578
Bombus terrestris	60,44116	17,41572
Bombus terrestris	60,47713	18,06927
Bombus terrestris	60,62851	17,17626
Bombus terrestris	63,227467	11,018852
Bombus terrestris	63,316006	9,432562
Bombus terrestris	63,46252	11,089693
Bombus terrestris	63,710501	9,542363
Bombus terrestris	63,843692	8,464591
Bombus terrestris	64,75624	21,03039
Bombus terrestris	64,924921	13,17798
Bombus terrestris	31,900398	34,814579
Bombus terrestris	36,773647	-4,962267
Bombus terrestris	37,235225	-8,181203
Bombus terrestris	38,762212	-8,367725
Bombus terrestris	42,03243	9,45882
Bombus terrestris	42,268367	3,051095

Bombus terrestris	43,348746	5,920158
Bombus terrestris	44,851427	0,946469
Bombus terrestris	50,725658	7,109997
Bombus terrestris	50,836384	-0,371883
Bombus terrestris	52,629761	-2,450912
Bombus terrestris	53,491561	-2,550858
Bombus terrestris	53,526757	-6,138813
Bombus terrestris	55,099536	-1,864097
Bombus terrestris	56,446515	12,75469
Bombus terrestris	57,9606	19,28952
Bombus terrestris	37,350185	-6,034832
Bombus terrestris	39,766118	-8,72244
Bombus terrestris	41,979605	12,05187
Bombus terrestris	50,386953	-3,8618
Bombus terrestris	51,486179	-3,204738
Bombus terrestris	51,785594	7,164383
Bombus terrestris	53,074658	7,377507
Bombus terrestris	54,066169	-4,619959
Bombus terrestris	55,826006	-4,265464
Bombus terrestris	57,006866	24,159972
Bombus terrestris	57,045329	-2,4913
Bombus terrestris	50,147388	-5,136642
Bombus terrestris	50,82682	-2,05137
Bombus terrestris	51,007366	-2,863162
Bombus terrestris	51,167145	-1,627957
Bombus terrestris	51,718933	-4,370274
Bombus terrestris	51,738674	-4,67042
Bombus terrestris	52,126621	-3,17813
Bombus terrestris	52,274769	-1,567904
Bombus terrestris	52,366555	-2,723924
Bombus terrestris	52,461453	-3,176306
Bombus terrestris	52,610344	-1,147394
Bombus terrestris	52,795131	-4,501224
Bombus terrestris	52,815653	-3,073525
Bombus terrestris	52,934731	-2,659577
Bombus terrestris	54,18857	-6,384366
Bombus terrestris	54,211347	-7,532435
Bombus terrestris	54,442755	-6,593262
Bombus terrestris	54,685852	-6,655574
Bombus terrestris	54,876124	-6,284678
Bombus terrestris	54,973899	-6,691567
Bombus terrestris	55,224522	-6,525569
Bombus terrestris	55,28401	-6,180715
Bombus terrestris	32,99889	35,392806
Bombus terrestris	36,736347	-2,862196
Bombus terrestris	36,82103	-2,430318

Bombus terrestris	37,992899	23,806628
Bombus terrestris	38,617962	-4,911867
Bombus terrestris	39,243709	-9,306088
Bombus terrestris	39,394341	-7,335304
Bombus terrestris	39,708587	-0,344868
Bombus terrestris	40,774807	-7,356008
Bombus terrestris	41,475904	-7,707188
Bombus terrestris	41,655215	-8,212603
Bombus terrestris	42,253349	11,099973
Bombus terrestris	42,827424	-2,655675
Bombus terrestris	45,750453	0,260672
Bombus terrestris	46,193922	12,896966
Bombus terrestris	46,51643	1,05023
Bombus terrestris	47,314146	-1,85616
Bombus terrestris	47,418495	-2,30496
Bombus terrestris	48,632227	2,331465
Bombus terrestris	49,504379	11,370715
Bombus terrestris	49,53887	5,821226
Bombus terrestris	49,750106	6,946937
Bombus terrestris	49,878184	36,441149
Bombus terrestris	50,256426	12,39567
Bombus terrestris	50,449631	-4,857922
Bombus terrestris	50,939172	-4,138555
Bombus terrestris	51,10452	-2,322512
Bombus terrestris	51,519455	9,841183
Bombus terrestris	51,567965	36,086581
Bombus terrestris	51,999044	-5,044739
Bombus terrestris	52,129154	-2,308546
Bombus terrestris	52,188771	5,3962
Bombus terrestris	52,606675	1,256767
Bombus terrestris	52,995882	-1,160976
Bombus terrestris	53,164231	8,206897
Bombus terrestris	53,559117	-1,474635
Bombus terrestris	54,22633	38,969079
Bombus terrestris	54,348634	10,578968
Bombus terrestris	55,479197	9,296887
Bombus terrestris	55,700968	11,031722
Bombus terrestris	55,767252	38,418715
Bombus terrestris	55,821963	11,415255
Bombus terrestris	55,98004	44,853139
Bombus terrestris	56,033562	48,727856
Bombus terrestris	57,804715	27,118474
Bombus terrestris	59,196005	17,793739
Bombus terrestris	60,03414	22,697267
Bombus terrestris	61,516183	24,015844
Bombus terrestris	37,72337	-8,780727

Bombus terrestris	41,635631	2,699449
Bombus terrestris	42,667882	-1,955593
Bombus terrestris	43,320295	11,342942
Bombus terrestris	44,314091	0,477281
Bombus terrestris	45,107807	7,682212
Bombus terrestris	46,619197	-0,875742
Bombus terrestris	48,859947	2,377765
Bombus terrestris	50,222549	5,349111
Bombus terrestris	50,737071	-0,771327
Bombus terrestris	50,996255	-0,817515
Bombus terrestris	51,894046	12,021427
Bombus terrestris	52,543848	-4,044576
Bombus terrestris	53,274453	-6,292179
Bombus terrestris	35,030377	24,758464
Bombus terrestris	41,864761	-6,990567
Bombus terrestris	42,489004	-8,87334
Bombus terrestris	42,600733	-5,585712
Bombus terrestris	42,767092	-1,588293
Bombus terrestris	42,95323	9,4216
Bombus terrestris	43,203705	-1,480229
Bombus terrestris	43,313	-4,1785
Bombus terrestris	43,40826	-5,82111
Bombus terrestris	46,477502	1,447341
Bombus terrestris	47,1309	2,54422
Bombus terrestris	47,437285	8,481288
Bombus terrestris	48,003581	-2,232692
Bombus terrestris	48,362651	-4,264794
Bombus terrestris	48,430001	-1,262138
Bombus terrestris	48,621592	-1,509752
Bombus terrestris	48,686925	-4,078208
Bombus terrestris	49,1436	2,18498
Bombus terrestris	49,396921	5,203973
Bombus terrestris	49,677319	6,339966
Bombus terrestris	50,06364	3,53528
Bombus terrestris	51,007075	7,096385
Bombus terrestris	51,205588	-3,493162
Bombus terrestris	51,301429	7,316237
Bombus terrestris	51,314842	6,18706
Bombus terrestris	52,026616	0,236842
Bombus terrestris	52,403583	13,103624
Bombus terrestris	53,327418	-0,676858
Bombus terrestris	53,569143	6,740792
Bombus terrestris	53,843938	8,736827
Bombus terrestris	53,935278	-0,229167
Bombus terrestris	54,089686	10,575008
Bombus terrestris	55,38042	14,13344

Bombus terrestris	55,739047	-2,164772
Bombus terrestris	55,80836	12,94064
Bombus terrestris	55,93137	13,54093
Bombus terrestris	55,94796	14,32288
Bombus terrestris	55,98359	38,102589
Bombus terrestris	56,01176	14,73216
Bombus terrestris	56,10986	15,46879
Bombus terrestris	56,16957	13,78684
Bombus terrestris	56,188988	10,203325
Bombus terrestris	56,67115	12,85146
Bombus terrestris	57,30322	11,90135
Bombus terrestris	57,31031	18,0719
Bombus terrestris	57,4197	16,23586
Bombus terrestris	57,65263	12,48987
Bombus terrestris	57,76246	16,63105
Bombus terrestris	57,83334	14,81805
Bombus terrestris	57,87252	18,98019
Bombus terrestris	58,0137	14,13338
Bombus terrestris	58,05915	11,81801
Bombus terrestris	58,12179	11,4513
Bombus terrestris	58,22845	12,76829
Bombus terrestris	58,27732	16,29628
Bombus terrestris	58,370755	26,276423
Bombus terrestris	58,379445	6,896798
Bombus terrestris	58,41096	15,49273
Bombus terrestris	58,51795	15,21736
Bombus terrestris	58,5573	11,26125
Bombus terrestris	58,61461	16,17092
Bombus terrestris	58,64336	16,56051
Bombus terrestris	58,646677	6,946232
Bombus terrestris	58,7093	13,84837
Bombus terrestris	58,76042	17,86093
Bombus terrestris	58,77361	16,86837
Bombus terrestris	58,89451	17,56787
Bombus terrestris	58,98239	18,13218
Bombus terrestris	59,08949	18,44708
Bombus terrestris	59,09335	16,48898
Bombus terrestris	59,176037	6,068767
Bombus terrestris	59,28757	12,77429
Bombus terrestris	59,36423	16,48581
Bombus terrestris	59,40105	13,57299
Bombus terrestris	59,61793	18,76228
Bombus terrestris	59,66023	12,71349
Bombus terrestris	59,69716	16,2049
Bombus terrestris	59,949859	11,216445
Bombus terrestris	60,11839	18,70294

Bombus terrestris	60,12242	10,300353
Bombus terrestris	60,14623	15,19627
Bombus terrestris	60,358748	10,469892
Bombus terrestris	60,458436	21,880675
Bombus terrestris	60,67535	15,48918
Bombus terrestris	60,698778	25,20961
Bombus terrestris	60,93346	16,70009
Bombus terrestris	61,145244	8,859149
Bombus terrestris	62,29503	17,30304
Bombus terrestris	62,40468	16,69388
Bombus terrestris	62,967054	9,771182
Bombus terrestris	62,98984	18,53014
Bombus terrestris	63,588974	10,709912
Bombus terrestris	41,895861	12,414068
Bombus terrestris	43,609865	3,809668
Bombus terrestris	52,205567	7,306963
Bombus terrestris	45,015485	0,536787
Bombus terrestris	47,720606	12,568021
Bombus terrestris	52,016358	6,024945
Bombus terrestris	54,776106	-1,59139
Bombus terrestris	60,207558	25,138072
Bombus terrestris	52,593622	6,840305
Bombus terrestris	54,828011	55,998154
Bombus terrestris	40,786992	-3,696447
Bombus terrestris	50,984012	6,677032
Bombus terrestris	51,685487	-3,443593
Bombus terrestris	52,749318	8,537954
Bombus terrestris	53,619086	8,045758
Bombus terrestris	32,636933	36,744347
Bombus terrestris	37,144596	-8,607598
Bombus terrestris	41,819721	-6,758722
Bombus terrestris	42,385414	-2,630555
Bombus terrestris	44,654177	8,142338
Bombus terrestris	49,216392	16,677943
Bombus terrestris	50,041851	36,209273
Bombus terrestris	50,703963	14,545343
Bombus terrestris	52,259133	9,044269
Bombus terrestris	53,319527	-0,949088
Bombus terrestris	56,094787	37,626686
Bombus terrestris	50,106832	19,945322
Bombus terrestris	52,755743	0,397594
Bombus terrestris	52,834156	0,869805
Bombus terrestris	54,749949	-1,2844
Bombus terrestris	57,028228	53,957169
Bombus terrestris	61,539017	25,149572
Bombus terrestris	50,099175	8,255297

Bombus terrestris	40,18281	-8,487128
Bombus terrestris	44,528362	1,221913
Bombus terrestris	50,219029	3,845697
Bombus terrestris	50,486102	3,654023
Bombus terrestris	52,319857	14,550538
Bombus terrestris	56,56928	16,14426
Bombus terrestris	58,508331	50,058054
Bombus terrestris	42,283675	27,746497
Bombus terrestris	48,3808	2,81452
Bombus terrestris	48,65047	2,06352
Bombus terrestris	49,263578	10,788356
Bombus terrestris	49,987635	16,597814
Bombus terrestris	51,67311	-1,854298
Bombus terrestris	52,640114	-0,686606
Bombus terrestris	54,342822	-4,467597
Bombus terrestris	56,19638	16,39812
Bombus terrestris	48,83416	15,895
Bombus terrestris	49,073451	19,273168
Bombus terrestris	53,079258	-0,777113
Bombus terrestris	55,063888	-3,572347
Bombus terrestris	46,990947	15,760903
Bombus terrestris	53,013237	-3,308246
Bombus terrestris	53,223584	-4,122778
Bombus terrestris	45,166348	8,322578
Bombus terrestris	51,189663	-4,006889
Bombus terrestris	55,944291	8,48326
Bombus terrestris	40,813909	-4,054203
Bombus terrestris	44,152481	11,927506
Bombus terrestris	48,832036	12,733107
Bombus terrestris	50,531193	15,371722
Bombus terrestris	36,161312	-5,590804
Bombus terrestris	36,551918	-5,275046
Bombus terrestris	36,77054	-4,40462
Bombus terrestris	38,36191	20,705817
Bombus terrestris	39,118748	-5,522991
Bombus terrestris	39,926531	-7,243324
Bombus terrestris	40,966835	24,746582
Bombus terrestris	41,442719	0,908894
Bombus terrestris	41,844257	-4,052921
Bombus terrestris	42,337666	-3,705759
Bombus terrestris	43,276273	-2,986088
Bombus terrestris	43,452242	-4,950357
Bombus terrestris	43,931973	5,638252
Bombus terrestris	44,199338	3,216113
Bombus terrestris	44,616149	4,558674
Bombus terrestris	44,680115	7,514582

Bombus terrestris	44,88664	1,399688
Bombus terrestris	46,122757	19,994173
Bombus terrestris	46,455349	11,543137
Bombus terrestris	46,461998	15,663086
Bombus terrestris	46,462585	17,42425
Bombus terrestris	46,529303	-0,324053
Bombus terrestris	46,547344	3,414522
Bombus terrestris	46,9391	16,0898
Bombus terrestris	47,170855	16,115592
Bombus terrestris	47,194618	23,119923
Bombus terrestris	47,337686	5,832893
Bombus terrestris	47,557308	8,02819
Bombus terrestris	47,585636	10,654904
Bombus terrestris	47,602142	11,506007
Bombus terrestris	47,607849	9,100939
Bombus terrestris	47,645206	10,176189
Bombus terrestris	47,677574	8,835127
Bombus terrestris	47,692924	11,008124
Bombus terrestris	47,807629	7,18847
Bombus terrestris	47,809116	7,62537
Bombus terrestris	47,817379	8,17884
Bombus terrestris	47,838936	12,213571
Bombus terrestris	47,871971	6,184187
Bombus terrestris	47,872917	10,671549
Bombus terrestris	47,908161	11,86362
Bombus terrestris	47,92	16,67
Bombus terrestris	47,922195	9,748889
Bombus terrestris	47,923939	8,533099
Bombus terrestris	47,926971	10,944845
Bombus terrestris	47,947817	6,778138
Bombus terrestris	47,951977	9,028214
Bombus terrestris	47,985889	7,882502
Bombus terrestris	48,023247	9,371338
Bombus terrestris	48,08189	11,201207
Bombus terrestris	48,101753	8,198341
Bombus terrestris	48,141289	5,764981
Bombus terrestris	48,176392	9,810277
Bombus terrestris	48,177303	12,44458
Bombus terrestris	48,182789	10,420294
Bombus terrestris	48,186703	14,274837
Bombus terrestris	48,214272	9,021013
Bombus terrestris	48,214661	7,830075
Bombus terrestris	48,245106	4,804889
Bombus terrestris	48,304207	10,15892
Bombus terrestris	48,308552	11,923265
Bombus terrestris	48,339031	10,693064

Bombus terrestris	48,354179	13,187921
Bombus terrestris	48,414989	9,74025
Bombus terrestris	48,422821	12,732607
Bombus terrestris	48,423996	11,078846
Bombus terrestris	48,462601	11,564359
Bombus terrestris	48,473549	8,511858
Bombus terrestris	48,50293	9,380457
Bombus terrestris	48,525272	12,374815
Bombus terrestris	48,536949	10,12905
Bombus terrestris	48,569431	10,637418
Bombus terrestris	48,569984	13,60309
Bombus terrestris	48,615299	12,081916
Bombus terrestris	48,637573	11,332852
Bombus terrestris	48,6478	6,288344
Bombus terrestris	48,649368	16,677179
Bombus terrestris	48,658649	9,684892
Bombus terrestris	48,715981	8,36911
Bombus terrestris	48,748718	10,994358
Bombus terrestris	48,767738	11,678025
Bombus terrestris	48,773914	9,281101
Bombus terrestris	48,794991	10,116776
Bombus terrestris	48,853508	10,349081
Bombus terrestris	48,871792	10,735687
Bombus terrestris	48,893639	8,604239
Bombus terrestris	48,946072	12,261662
Bombus terrestris	49,004555	8,215661
Bombus terrestris	49,046516	0,731632
Bombus terrestris	49,06263	13,10781
Bombus terrestris	49,070606	9,42495
Bombus terrestris	49,083237	11,897749
Bombus terrestris	49,115192	7,862794
Bombus terrestris	49,116356	9,71433
Bombus terrestris	49,133717	11,13351
Bombus terrestris	49,137238	9,166603
Bombus terrestris	49,170174	10,081612
Bombus terrestris	49,175751	6,809278
Bombus terrestris	49,198559	7,074143
Bombus terrestris	49,206139	8,87732
Bombus terrestris	49,210361	12,706944
Bombus terrestris	49,226154	7,498694
Bombus terrestris	49,275711	10,520683
Bombus terrestris	49,27792	11,480637
Bombus terrestris	49,374485	9,751786
Bombus terrestris	49,37899	11,0188
Bombus terrestris	49,381184	7,823829
Bombus terrestris	49,395023	3,682669

Bombus terrestris	49,412579	9,45946
Bombus terrestris	49,440338	11,851599
Bombus terrestris	49,44561	12,19842
Bombus terrestris	49,466385	8,994238
Bombus terrestris	49,532871	10,119782
Bombus terrestris	49,552704	10,715473
Bombus terrestris	49,626846	3,493586
Bombus terrestris	49,62883	7,826706
Bombus terrestris	49,641079	12,744075
Bombus terrestris	49,648193	4,943781
Bombus terrestris	49,683548	6,689311
Bombus terrestris	49,723755	12,160918
Bombus terrestris	49,742867	10,349475
Bombus terrestris	49,77351	9,955749
Bombus terrestris	49,806232	11,032141
Bombus terrestris	49,811523	15,468683
Bombus terrestris	49,852718	2,292694
Bombus terrestris	49,859566	9,687041
Bombus terrestris	49,868805	8,922813
Bombus terrestris	49,891193	11,321983
Bombus terrestris	49,898834	7,496304
Bombus terrestris	49,916645	11,59028
Bombus terrestris	49,996426	4,444117
Bombus terrestris	50,003975	7,075129
Bombus terrestris	50,021851	9,45444
Bombus terrestris	50,037857	12,897882
Bombus terrestris	50,047821	10,937808
Bombus terrestris	50,129501	14,919367
Bombus terrestris	50,133926	10,03569
Bombus terrestris	50,136547	17,204523
Bombus terrestris	50,150627	17,446222
Bombus terrestris	50,161263	10,539705
Bombus terrestris	50,174988	9,755787
Bombus terrestris	50,183266	6,830325
Bombus terrestris	50,195457	11,779058
Bombus terrestris	50,211376	7,563106
Bombus terrestris	50,240143	12,034668
Bombus terrestris	50,245995	6,327193
Bombus terrestris	50,246124	8,006924
Bombus terrestris	50,284718	6,590499
Bombus terrestris	50,319283	1,62591
Bombus terrestris	50,345814	9,03417
Bombus terrestris	50,36911	8,267365
Bombus terrestris	50,403378	10,219502
Bombus terrestris	50,428612	8,496519
Bombus terrestris	50,45118	7,539693

Bombus terrestris	50,474968	11,65606
Bombus terrestris	50,493065	8,793354
Bombus terrestris	50,494331	16,270685
Bombus terrestris	50,495674	12,602997
Bombus terrestris	50,497383	10,899158
Bombus terrestris	50,517899	9,688787
Bombus terrestris	50,53545	11,423257
Bombus terrestris	50,54192	7,95626
Bombus terrestris	50,54446	12,961441
Bombus terrestris	50,589573	12,127715
Bombus terrestris	50,601402	13,222449
Bombus terrestris	50,613575	9,38093
Bombus terrestris	50,647839	13,513117
Bombus terrestris	50,718578	10,11593
Bombus terrestris	50,721012	9,017497
Bombus terrestris	50,728142	6,665977
Bombus terrestris	50,758026	11,696573
Bombus terrestris	50,764839	9,695606
Bombus terrestris	50,775467	12,885201
Bombus terrestris	50,781094	7,747238
Bombus terrestris	50,802052	8,364681
Bombus terrestris	50,841736	10,682971
Bombus terrestris	50,856617	11,203444
Bombus terrestris	50,857895	14,177861
Bombus terrestris	50,863251	12,068326
Bombus terrestris	50,888618	14,794452
Bombus terrestris	50,903381	10,911051
Bombus terrestris	50,904186	7,398023
Bombus terrestris	50,91259	6,357742
Bombus terrestris	50,930241	12,55291
Bombus terrestris	50,9496	9,066032
Bombus terrestris	50,965405	13,646123
Bombus terrestris	50,979145	10,416971
Bombus terrestris	50,983841	8,624364
Bombus terrestris	50,984142	5,978182
Bombus terrestris	51,023842	9,41722
Bombus terrestris	51,033386	11,4469
Bombus terrestris	51,061275	14,502603
Bombus terrestris	51,10957	7,859632
Bombus terrestris	51,123707	9,664818
Bombus terrestris	51,127167	13,899746
Bombus terrestris	51,151867	12,097353
Bombus terrestris	51,157089	11,12044
Bombus terrestris	51,176567	12,935577
Bombus terrestris	51,193672	12,429173
Bombus terrestris	51,199265	7,628346

Bombus terrestris	51,203079	10,749594
Bombus terrestris	51,271511	8,846566
Bombus terrestris	51,284275	13,615705
Bombus terrestris	51,287285	14,830814
Bombus terrestris	51,299671	8,61145
Bombus terrestris	51,326557	14,341564
Bombus terrestris	51,328663	9,332645
Bombus terrestris	51,389084	11,784644
Bombus terrestris	51,414742	11,55755
Bombus terrestris	51,437183	10,570906
Bombus terrestris	51,461323	12,621883
Bombus terrestris	51,462025	10,939503
Bombus terrestris	51,482182	12,02705
Bombus terrestris	51,514732	11,233779
Bombus terrestris	51,521999	14,71481
Bombus terrestris	51,550522	15,044692
Bombus terrestris	51,555382	7,660787
Bombus terrestris	51,555481	12,973543
Bombus terrestris	51,565289	3,769283
Bombus terrestris	51,629475	6,832173
Bombus terrestris	51,642338	7,96416
Bombus terrestris	51,716057	11,785784
Bombus terrestris	51,729862	14,301203
Bombus terrestris	51,735229	9,38141
Bombus terrestris	51,750175	14,038451
Bombus terrestris	51,764206	9,68662
Bombus terrestris	51,78231	14,642581
Bombus terrestris	51,790337	13,399846
Bombus terrestris	51,791519	12,944701
Bombus terrestris	51,793327	10,324663
Bombus terrestris	51,808411	8,413729
Bombus terrestris	51,825661	10,787613
Bombus terrestris	51,853802	6,596886
Bombus terrestris	51,875595	9,104794
Bombus terrestris	51,883621	12,679816
Bombus terrestris	52,019512	11,735504
Bombus terrestris	52,025112	9,638715
Bombus terrestris	52,029671	13,226231
Bombus terrestris	52,039356	11,244711
Bombus terrestris	52,05048	9,371914
Bombus terrestris	52,055931	14,218391
Bombus terrestris	52,079475	12,248265
Bombus terrestris	52,118519	9,988528
Bombus terrestris	52,158451	13,873343
Bombus terrestris	52,220234	13,477102
Bombus terrestris	52,350555	17,731867

Bombus terrestris	52,367317	8,143744
Bombus terrestris	52,383968	12,529774
Bombus terrestris	52,427673	9,472419
Bombus terrestris	52,457588	8,373047
Bombus terrestris	52,458405	10,178729
Bombus terrestris	52,50029	14,153741
Bombus terrestris	52,502148	11,401205
Bombus terrestris	52,53228	13,748584
Bombus terrestris	52,536549	7,392727
Bombus terrestris	52,635162	13,040776
Bombus terrestris	52,641243	9,235888
Bombus terrestris	52,64563	8,02562
Bombus terrestris	52,678692	9,632313
Bombus terrestris	52,71669	23,794342
Bombus terrestris	52,733253	8,270396
Bombus terrestris	52,733757	10,607808
Bombus terrestris	52,738068	4,811945
Bombus terrestris	52,759834	10,954571
Bombus terrestris	52,769176	11,878186
Bombus terrestris	52,787918	12,789147
Bombus terrestris	52,801407	8,95208
Bombus terrestris	52,82592	13,792381
Bombus terrestris	52,826031	12,39464
Bombus terrestris	52,857635	14,69964
Bombus terrestris	52,886589	9,312796
Bombus terrestris	52,896812	13,200075
Bombus terrestris	52,897709	11,305903
Bombus terrestris	52,904099	6,750911
Bombus terrestris	52,99398	10,988885
Bombus terrestris	53,011211	11,765489
Bombus terrestris	53,070721	12,473578
Bombus terrestris	53,072498	14,265765
Bombus terrestris	53,077702	8,634095
Bombus terrestris	53,102283	13,496683
Bombus terrestris	53,103672	10,069622
Bombus terrestris	53,10458	5,951079
Bombus terrestris	53,118656	8,919039
Bombus terrestris	53,122749	11,23426
Bombus terrestris	53,148529	14,040461
Bombus terrestris	53,155777	12,183261
Bombus terrestris	53,181061	4,856581
Bombus terrestris	53,23204	10,888083
Bombus terrestris	53,303875	9,261658
Bombus terrestris	53,307686	7,484095
Bombus terrestris	53,321209	13,007988
Bombus terrestris	53,339184	6,877375

Bombus terrestris	53,35659	7,190046
Bombus terrestris	53,358982	8,63646
Bombus terrestris	53,389046	9,005484
Bombus terrestris	53,389763	12,647696
Bombus terrestris	53,404751	9,707766
Bombus terrestris	53,44384	11,185065
Bombus terrestris	53,513016	8,353732
Bombus terrestris	53,532703	10,674818
Bombus terrestris	53,533211	11,477525
Bombus terrestris	53,548088	14,289987
Bombus terrestris	53,552067	13,702589
Bombus terrestris	53,553219	12,962824
Bombus terrestris	53,564556	13,417495
Bombus terrestris	53,582035	7,181473
Bombus terrestris	53,615814	9,313073
Bombus terrestris	53,751266	9,671016
Bombus terrestris	53,751797	7,565335
Bombus terrestris	53,759335	10,8726
Bombus terrestris	53,788219	12,58704
Bombus terrestris	53,824074	11,937289
Bombus terrestris	53,825672	9,946454
Bombus terrestris	53,848335	11,409084
Bombus terrestris	53,933708	14,01739
Bombus terrestris	53,966827	13,741073
Bombus terrestris	53,996502	12,194328
Bombus terrestris	54,003452	11,668254
Bombus terrestris	54,01868	-0,574195
Bombus terrestris	54,050945	12,780422
Bombus terrestris	54,068497	9,202782
Bombus terrestris	54,070267	10,231567
Bombus terrestris	54,082436	13,441973
Bombus terrestris	54,085743	9,606519
Bombus terrestris	54,103748	12,542224
Bombus terrestris	54,330833	8,78116
Bombus terrestris	54,362236	13,707039
Bombus terrestris	54,413811	12,933923
Bombus terrestris	54,487438	9,635831
Bombus terrestris	54,630512	13,22485
Bombus terrestris	54,682419	9,319768
Bombus terrestris	54,770138	9,761754
Bombus terrestris	54,860828	8,939525
Bombus terrestris	54,94249	23,666901
Bombus terrestris	56,40415	14,00211
Bombus terrestris	56,54799	13,08272
Bombus terrestris	56,74656	15,88463
Bombus terrestris	56,81525	16,33245

Bombus terrestris	57,02492	16,09758
Bombus terrestris	57,25791	15,91854
Bombus terrestris	57,30027	16,99899
Bombus terrestris	57,34362	15,5637
Bombus terrestris	57,41903	15,0603
Bombus terrestris	57,66784	18,68114
Bombus terrestris	57,81823	16,04525
Bombus terrestris	57,9183	12,54089
Bombus terrestris	57,99807	14,43473
Bombus terrestris	58,1526	16,7994
Bombus terrestris	58,154351	8,031235
Bombus terrestris	58,30363	13,6079
Bombus terrestris	58,34823	11,93835
Bombus terrestris	58,437342	6,011095
Bombus terrestris	58,49117	13,16176
Bombus terrestris	58,832197	23,581478
Bombus terrestris	59,14996	12,51766
Bombus terrestris	59,17897	17,41955
Bombus terrestris	59,26606	15,55209
Bombus terrestris	59,297515	11,100311
Bombus terrestris	59,35063	14,06026
Bombus terrestris	59,405148	9,083405
Bombus terrestris	59,413226	10,210871
Bombus terrestris	59,547206	11,575933
Bombus terrestris	59,70265	13,60968
Bombus terrestris	59,741481	11,804923
Bombus terrestris	60,06378	13,55988
Bombus terrestris	60,14746	16,1675
Bombus terrestris	60,2001	16,48749
Bombus terrestris	60,45885	16,04792
Bombus terrestris	60,572612	11,271737
Bombus terrestris	60,681167	8,576351
Bombus terrestris	60,7156	15,05785
Bombus terrestris	60,80024	11,343952
Bombus terrestris	61,01932	14,50897
Bombus terrestris	62,64312	14,37159
Bombus terrestris	63,31847	18,71183
Bombus terrestris	63,494401	10,066719
Bombus terrestris	63,809552	11,576517
Bombus terrestris	46,68505	-1,51952
Bombus terrestris	45,518635	9,225023
Bombus terrestris	47,073925	15,437339
Bombus terrestris	41,468023	-2,863427
Bombus terrestris	44,981388	1,839362
Bombus terrestris	54,230719	37,639725
Bombus terrestris	58,0928	25,6967

Bombus terrestris	32,855046	35,753877
Bombus terrestris	30,749069	35,275249
Bombus terrestris	35,021544	25,862728
Bombus terrestris	39,211429	26,048098
Bombus terrestris	42,468045	9,475322
Bombus terrestris	42,646336	23,36507
Bombus terrestris	43,787807	7,631482
Bombus terrestris	44,837569	7,240146
Bombus terrestris	44,896847	11,662441
Bombus terrestris	45,967302	-1,04728
Bombus terrestris	47,79611	35,144995
Bombus terrestris	50,758399	2,338478
Bombus terrestris	53,185725	-2,3071
Bombus terrestris	54,486843	48,823504
Bombus terrestris	55,02974	36,914095
Bombus terrestris	56,073168	44,558411
Bombus terrestris	56,160115	44,172174
Bombus terrestris	57,213703	41,924475
Bombus terrestris	54,893713	34,99982
Bombus terrestris	45,717667	9,814421
Bombus terrestris	47,274829	14,774452
Bombus terrestris	60,625161	6,432117
Bombus terrestris	54,206081	-0,808012
Bombus terrestris	50,207121	33,203012
Bombus terrestris	57,329222	-3,613351
Bombus terrestris	31,791856	35,155002
Bombus terrestris	61,97407	17,25131
Bombus terrestris	53,98509	38,192564
Bombus terrestris	56,458706	10,808745
Bombus terrestris	52,538502	25,875086
Bombus terrestris	56,547911	-5,442868
Bombus terrestris	57,27876	18,37799
Bombus terrestris	47,30546	0,38208
Bombus terrestris	47,312	0,95759
Bombus terrestris	48,55345	-2,00278
Bombus terrestris	48,78371	-3,52429
Bombus terrestris	56,2555	15,10512
Bombus terrestris	56,29068	12,48676
Bombus terrestris	57,12729	14,70397
Bombus terrestris	57,27663	18,63327
Bombus terrestris	57,34778	12,57479
Bombus terrestris	57,41088	14,10388
Bombus terrestris	57,50964	16,66192
Bombus terrestris	57,5739	15,3375
Bombus terrestris	57,88619	11,58368
Bombus terrestris	57,92463	12,89762

Bombus terrestris	58,01702	15,42468
Bombus terrestris	58,07477	13,49651
Bombus terrestris	58,335989	6,646455
Bombus terrestris	58,39044	8,720467
Bombus terrestris	58,626208	9,038624
Bombus terrestris	58,62787	12,3816
Bombus terrestris	58,84785	11,16587
Bombus terrestris	58,885332	8,95332
Bombus terrestris	58,88629	13,23492
Bombus terrestris	59,04695	17,16417
Bombus terrestris	59,06354	14,04382
Bombus terrestris	59,19946	12,21931
Bombus terrestris	59,26005	18,75151
Bombus terrestris	59,38469	13,80031
Bombus terrestris	59,533822	9,838493
Bombus terrestris	59,57948	14,70653
Bombus terrestris	59,78884	12,34211
Bombus terrestris	60,19264	14,20476
Bombus terrestris	60,37996	14,62464
Bombus terrestris	60,74652	12,81777
Bombus terrestris	60,754007	10,615203
Bombus terrestris	61,173107	10,406093
Bombus terrestris	62,76613	15,49477
Bombus terrestris	63,16044	14,6664
Bombus terrestris	63,70596	13,86807
Bombus terrestris	63,79768	20,869
Bombus terrestris	40,743468	17,466654
Bombus terrestris	55,002987	-7,323955
Bombus terrestris	48,788853	-3,049769
Bombus terrestris	37,941611	27,432477
Bombus terrestris	40,820443	23,359198
Bombus terrestris	43,138953	3,079218
Bombus terrestris	45,705431	16,387305
Bombus terrestris	47,270355	39,691881
Bombus terrestris	51,441522	21,962337
Bombus terrestris	55,321749	37,126214
Bombus terrestris	33,6385	35,548095
Bombus terrestris	47,392662	0,696247
Bombus terrestris	48,878222	2,087767
Bombus terrestris	51,1567	4,4845
Bombus terrestris	59,01343	15,59024
Bombus terrestris	49,70858	-2,20044
Bombus terrestris	49,907425	-6,305357
Bombus terrestris	50,463429	-3,565311
Bombus terrestris	50,595348	-1,957579
Bombus terrestris	50,622311	-2,463657

Bombus terrestris	50,676701	-3,862558
Bombus terrestris	50,688519	-4,685651
Bombus terrestris	50,733417	-2,921696
Bombus terrestris	51,077877	-2,548127
Bombus terrestris	51,361721	1,427496
Bombus terrestris	51,427731	-1,723803
Bombus terrestris	51,604958	-2,233884
Bombus terrestris	51,765388	-3,201273
Bombus terrestris	51,780281	-2,692865
Bombus terrestris	51,841122	1,238752
Bombus terrestris	51,860661	0,513873
Bombus terrestris	51,863143	-5,336796
Bombus terrestris	52,036304	-4,084768
Bombus terrestris	52,101512	-3,813972
Bombus terrestris	52,202541	-0,728369
Bombus terrestris	52,262344	-0,959642
Bombus terrestris	52,315929	0,525621
Bombus terrestris	52,560635	-0,316772
Bombus terrestris	52,582376	0,676729
Bombus terrestris	52,681984	-0,023626
Bombus terrestris	52,708766	-3,389066
Bombus terrestris	52,794788	-4,72609
Bombus terrestris	52,8367	-4,05232
Bombus terrestris	52,87847	-2,241412
Bombus terrestris	53,018726	-1,391757
Bombus terrestris	53,100834	-0,185223
Bombus terrestris	53,208733	-0,43973
Bombus terrestris	53,348088	-3,376717
Bombus terrestris	53,718643	-0,455763
Bombus terrestris	53,929539	-0,821153
Bombus terrestris	54,064747	-1,978583
Bombus terrestris	54,093208	-2,26753
Bombus terrestris	54,102808	-6,622608
Bombus terrestris	54,24448	-1,066949
Bombus terrestris	54,333103	-1,446339
Bombus terrestris	54,371402	-7,221424
Bombus terrestris	54,408703	-1,745751
Bombus terrestris	54,424148	-7,865944
Bombus terrestris	54,753029	-2,186414
Bombus terrestris	54,998081	-6,007629
Bombus terrestris	55,120082	-6,91853
Bombus terrestris	55,201969	-4,224913
Bombus terrestris	55,310959	-3,503022
Bombus terrestris	55,427151	-4,65169
Bombus terrestris	55,443413	-5,141445
Bombus terrestris	55,712315	-2,760754

Bombus terrestris	55,746605	-4,913972
Bombus terrestris	55,825314	-6,355904
Bombus terrestris	55,99604	-4,895825
Bombus terrestris	56,007607	-5,458307
Bombus terrestris	56,164261	-3,921082
Bombus terrestris	56,173908	-4,153552
Bombus terrestris	56,29134	-3,072714
Bombus terrestris	56,345745	-5,116543
Bombus terrestris	56,565296	-3,181346
Bombus terrestris	56,64135	-3,806653
Bombus terrestris	56,689079	-4,140432
Bombus terrestris	56,989693	-3,487721
Bombus terrestris	57,056782	-6,504313
Bombus terrestris	57,074183	-4,024772
Bombus terrestris	57,272736	-2,414537
Bombus terrestris	57,282749	-4,451891
Bombus terrestris	57,597458	-3,599468
Bombus terrestris	57,61533	-3,104731
Bombus terrestris	57,741604	-4,067857
Bombus terrestris	58,477596	-4,417962
Bombus terrestris	58,596245	-3,381547
Bombus terrestris	58,42803	14,22785
Bombus terrestris	58,51697	14,53007
Bombus terrestris	58,90405	16,05221
Bombus terrestris	59,17121	14,37152
Bombus terrestris	59,79507	15,06381
Bombus terrestris	60,52921	16,29396
Bombus terrestris	60,96207	12,47137
Bombus terrestris	62,4918	17,53122
Bombus terrestris	63,624151	8,738703
Bombus terrestris	55,81926	37,230922
Bombus terrestris	40,161812	9,506725
Bombus terrestris	49,431437	26,973507
Bombus terrestris	40,789233	8,478277
Bombus terrestris	52,262968	43,852804
Bombus terrestris	58,20187	16,00547
Bombus terrestris	47,98348	3,40902
Bombus terrestris	42,928651	-5,274897
Bombus terrestris	46,87817	2,3344
Bombus terrestris	36,581177	2,365637
Bombus terrestris	42,44379	27,466622
Bombus terrestris	43,13038	-0,43525
Bombus terrestris	48,35773	2,38445
Bombus terrestris	48,36613	2,12732
Bombus terrestris	48,4184	3,1725
Bombus terrestris	51,47601	-9,75094

Bombus terrestris	51,47917	-9,37967
Bombus terrestris	51,548616	-8,948562
Bombus terrestris	51,64147	-8,55253
Bombus terrestris	51,71856	-9,35366
Bombus terrestris	51,80521	-8,1375
Bombus terrestris	51,863831	-8,865172
Bombus terrestris	51,9252	-8,39756
Bombus terrestris	52,0957	-7,92631
Bombus terrestris	52,1047	-9,78422
Bombus terrestris	52,11901	-6,61234
Bombus terrestris	52,12767	-10,13271
Bombus terrestris	52,12919	-7,10706
Bombus terrestris	52,15727	-7,49465
Bombus terrestris	52,19701	-9,54804
Bombus terrestris	52,21016	-8,9955
Bombus terrestris	52,302843	-8,469095
Bombus terrestris	52,362169	-7,938344
Bombus terrestris	52,37477	-9,31277
Bombus terrestris	52,41902	-8,20695
Bombus terrestris	52,505703	-9,681846
Bombus terrestris	52,510818	-7,125117
Bombus terrestris	52,517064	-6,345481
Bombus terrestris	52,51939	-6,73234
Bombus terrestris	52,582293	-9,038574
Bombus terrestris	52,626231	-8,499124
Bombus terrestris	52,736245	-7,236085
Bombus terrestris	52,79283	-8,23791
Bombus terrestris	52,8145	-9,31923
Bombus terrestris	52,89143	-6,864888
Bombus terrestris	52,985007	-8,941965
Bombus terrestris	53,008411	-7,2838
Bombus terrestris	53,067712	-7,572671
Bombus terrestris	53,07767	-8,23947
Bombus terrestris	53,095853	-7,911171
Bombus terrestris	53,11815	-9,67363
Bombus terrestris	53,183453	-6,608873
Bombus terrestris	53,21927	-6,99921
Bombus terrestris	53,26185	-9,0647
Bombus terrestris	53,327253	-7,978975
Bombus terrestris	53,3908	-7,38449
Bombus terrestris	53,42981	-6,57597
Bombus terrestris	53,44761	-10,10041
Bombus terrestris	53,49362	-6,92948
Bombus terrestris	53,50335	-7,66919
Bombus terrestris	53,6799	-8,00074
Bombus terrestris	53,74193	-8,4554

Bombus terrestris	53,79916	-8,69884
Bombus terrestris	53,82273	-7,5285
Bombus terrestris	53,83263	-7,07122
Bombus terrestris	53,85056	-9,31815
Bombus terrestris	53,919871	-9,080605
Bombus terrestris	53,97545	-8,47319
Bombus terrestris	54,030125	-6,159525
Bombus terrestris	54,234942	-10,065808
Bombus terrestris	54,25867	-6,88059
Bombus terrestris	54,30865	-8,18049
Bombus terrestris	54,45163	-8,46324
Bombus terrestris	54,56566	-8,11206
Bombus terrestris	54,6542	-7,35307
Bombus terrestris	54,80734	-8,05829
Bombus terrestris	54,83538	-7,6325
Bombus terrestris	54,93056	-8,42816
Bombus terrestris	55,03854	-7,96392
Bombus terrestris	55,595111	11,98025
Bombus terrestris	56,13344	15,881
Bombus terrestris	56,4846	13,52954
Bombus terrestris	56,53645	15,40402
Bombus terrestris	56,77788	15,44504
Bombus terrestris	56,79536	13,30711
Bombus terrestris	56,98398	14,31488
Bombus terrestris	57,07208	13,7104
Bombus terrestris	57,12397	13,25392
Bombus terrestris	57,14695	14,02684
Bombus terrestris	57,37102	14,46769
Bombus terrestris	57,46605	13,25815
Bombus terrestris	57,72778	13,40647
Bombus terrestris	57,73951	15,79411
Bombus terrestris	57,78751	13,90061
Bombus terrestris	58,13841	13,83751
Bombus terrestris	58,59393	15,73435
Bombus terrestris	58,76256	11,89693
Bombus terrestris	58,85257	12,16784
Bombus terrestris	59,37742	16,77113
Bombus terrestris	59,44292	19,20997
Bombus terrestris	59,60412	11,048272
Bombus terrestris	59,87273	19,01621
Bombus terrestris	59,95069	12,91797
Bombus terrestris	59,95814	17,35078
Bombus terrestris	59,96297	13,1883
Bombus terrestris	60,03608	15,59169
Bombus terrestris	60,08743	5,248865
Bombus terrestris	60,19546	17,9052

Bombus terrestris	60,32685	16,7985
Bombus terrestris	60,40589	15,72929
Bombus terrestris	60,45669	14,38441
Bombus terrestris	60,50779	17,71044
Bombus terrestris	60,551499	9,15448
Bombus terrestris	60,57588	16,78325
Bombus terrestris	60,578726	27,199247
Bombus terrestris	60,623631	26,206883
Bombus terrestris	60,753335	4,913821
Bombus terrestris	61,20568	16,75249
Bombus terrestris	61,34994	16,39244
Bombus terrestris	62,15995	15,67619
Bombus terrestris	62,46192	16,37155
Bombus terrestris	62,48141	17,00435
Bombus terrestris	62,51189	16,01622
Bombus terrestris	62,88529	18,11118
Bombus terrestris	62,92545	17,79651
Bombus terrestris	63,30327	13,83008
Bombus terrestris	63,59069	19,33124
Bombus terrestris	55,669108	36,714265
Bombus terrestris	56,155194	10,720715
Bombus terrestris	40,24338	-7,719127
Bombus terrestris	51,19152	36,313571
Bombus terrestris	53,424769	56,039819
Bombus terrestris	53,682774	46,450124
Bombus terrestris	56,219667	35,951232
Bombus terrestris	62,27136	12,892
Bombus terrestris	36,99045	-6,486261
Bombus terrestris	56,721075	37,137165
Bombus terrestris	50,592162	36,553323
Bombus terrestris	50,101955	13,874501
Bombus terrestris	61,84416	16,56876
Bombus terrestris	64,075392	11,236397
Bombus terrestris	50,15675	6,04952
Bombus terrestris	41,780999	-8,521059
Bombus terrestris	55,511111	9,638281
Bombus terrestris	37,49649	15,050258
Bombus terrestris	38,331356	26,314395
Bombus terrestris	39,229749	8,406179
Bombus terrestris	40,357829	-5,126972
Bombus terrestris	42,636152	2,754003
Bombus terrestris	41,127855	9,058127
Bombus terrestris	47,086228	7,52141
Bombus terrestris	45,04112	9,677755
Bombus terrestris	38,751615	-9,448757
Bombus terrestris	37,033533	-7,820609

Bombus terrestris	48,730287	44,545615
Bombus terrestris	54,700541	46,519754
Bombus terrestris	47,499468	19,033218
Bombus terrestris	56,4093	14,26403
Bombus terrestris	57,09349	12,65447
Bombus terrestris	59,308548	4,881176
Bombus terrestris	60,00321	13,96278
Bombus terrestris	62,64029	17,94979
Bombus terrestris	38,909163	-0,229156
Bombus terrestris	46,54516	2,26519
Bombus terrestris	39,336887	-0,34152
Bombus terrestris	40,651887	22,968535
Bombus terrestris	45,638549	8,663505
Bombus terrestris	37,768829	27,027031
Bombus terrestris	38,056019	-3,99736
Bombus terrestris	42,170784	0,028496
Bombus terrestris	43,919411	8,045254
Bombus terrestris	44,616501	22,669601
Bombus terrestris	46,210011	6,084023
Bombus terrestris	47,595108	12,149183
Bombus terrestris	47,618431	16,793289
Bombus terrestris	47,674122	9,707213
Bombus terrestris	48,603573	6,932116
Bombus terrestris	49,166721	14,70932
Bombus terrestris	50,840675	5,680047
Bombus terrestris	52,991127	10,297623
Bombus terrestris	53,333332	6,287613
Bombus terrestris	53,621967	12,303334
Bombus terrestris	53,922234	8,499985
Bombus terrestris	54,41024	12,592049
Bombus terrestris	54,906422	8,6483
Bombus terrestris	60,14885	12,54102
Bombus terrestris	60,44875	13,22286
Bombus terrestris	63,121321	7,84196
Bombus terrestris	64,231953	12,311959
Bombus terrestris	54,736798	20,515427
Bombus terrestris	47,5811	2,44519
Bombus terrestris	47,34606	2,19603
Bombus terrestris	41,91497	3,20151
Bombus terrestris	43,51389	16,45583
Bombus terrestris	44,43333	26,1
Bombus terrestris	37,615163	-6,826522
Bombus terrestris	47,22729	1,95561
Bombus terrestris	42,606631	-8,637265
Bombus terrestris	59,228269	30,363278
Bombus terrestris	53,313476	34,249813

Bombus terrestris	39,623174	-7,911975
Bombus terrestris	57,273932	37,754761
Bombus terrestris	39,051066	-7,822598
Bombus terrestris	43,449865	1,429865
Bombus terrestris	36,687569	-6,058742
Bombus terrestris	40,368008	-8,023072
Bombus terrestris	55,822749	52,439588
Bombus terrestris	54,889275	37,444931
Bombus terrestris	64,736763	26,401077
Bombus terrestris	46,668056	14,346111
Bombus terrestris	48,416668	14,283334
Bombus terrestris	51,244	5,28
Bombus terrestris	61,019234	9,97828
Bombus terrestris	63,976279	9,07594
Bombus terrestris	64,22816	21,0678
Bombus terrestris	46,90579	2,79275
Bombus terrestris	54,925898	-3,946863
Bombus terrestris	54,406877	-2,056318
Bombus terrestris	40,390332	-6,912564
Bombus terrestris	52,277297	-3,562422
Bombus terrestris	54,650775	-7,032507
Bombus terrestris	49,30308	1,14395
Bombus terrestris	41,529629	1,65514
Bombus terrestris	45,77893	4,85325
Bombus terrestris	41,690736	2,165395
Bombus terrestris	39,742645	3,430195
Bombus terrestris	45,271553	0,169751
Bombus terrestris	58,382562	24,491375
Bombus terrestris	39,774475	3,129261
Bombus terrestris	46,021112	4,085623
Bombus terrestris	53,47	22,69
Bombus terrestris	53,63	22,95
Bombus terrestris	53,793822	-9,981081
Bombus terrestris	53,99867	-8,206598
Bombus terrestris	58,91738	5,509323
Bombus terrestris	62,22214	25,69899
Limodorum abortivum	42,581352	2,418193
Limodorum abortivum	40,249148	-5,891925
Limodorum abortivum	42,541239	2,325089
Limodorum abortivum	42,593136	2,463928
Limodorum abortivum	41,818669	-1,823135
Limodorum abortivum	42,624378	0,332738
Limodorum abortivum	36,87	-2,72
Limodorum abortivum	36,94875726	-3,281141855
Limodorum abortivum	41,754	2,489
Limodorum abortivum	42,50891752	1,568859567

Limodorum abortivum	42,78890052	-0,701193092
Limodorum abortivum	42,1748	9,09235
Limodorum abortivum	42,29278	8,93616
Limodorum abortivum	42,50597	8,98477
Limodorum abortivum	45,54222	6,60697
Limodorum abortivum	45,60432	5,95478
Limodorum abortivum	36,594665	4,575549
Limodorum abortivum	42,372055	9,362118
Limodorum abortivum	45,108573	5,655072
Limodorum abortivum	46,005116	12,326586
Limodorum abortivum	46,00861	11,178753
Limodorum abortivum	37,704389	15,048444
Limodorum abortivum	44,569864	6,54422
Limodorum abortivum	44,909294	6,653709
Limodorum abortivum	45,941724	6,661586
Limodorum abortivum	45,971799	10,943455
Limodorum abortivum	46,166808	5,894678
Limodorum abortivum	46,235897	6,93434
Limodorum abortivum	46,313818	7,832083
Limodorum abortivum	46,315787	7,635948
Limodorum abortivum	46,345111	6,959925
Limodorum abortivum	46,404014	6,930673
Limodorum abortivum	46,481288	11,227363
Limodorum abortivum	42,116842	9,113114
Limodorum abortivum	44,555697	6,36558
Limodorum abortivum	45,062561	5,754388
Limodorum abortivum	45,147712	7,126192
Limodorum abortivum	45,232764	5,787389
Limodorum abortivum	45,258458	5,838337
Limodorum abortivum	45,647287	10,596545
Limodorum abortivum	45,667303	6,316154
Limodorum abortivum	45,716376	7,691311
Limodorum abortivum	45,786659	10,735803
Limodorum abortivum	45,899922	5,72845
Limodorum abortivum	46,203104	7,413767
Limodorum abortivum	46,237651	5,965506
Limodorum abortivum	46,391663	11,237882
Limodorum abortivum	45,243955	6,41516
Limodorum abortivum	45,978726	10,314784
Limodorum abortivum	46,181505	7,183144
Limodorum abortivum	46,256412	7,349964
Limodorum abortivum	46,286147	7,498655
Limodorum abortivum	46,080815	12,554011
Limodorum abortivum	46,586493	11,207104
Limodorum abortivum	45,321632	5,653531
Limodorum abortivum	45,649763	5,847303

Limodorum abortivum	45,754587	7,576257
Limodorum abortivum	46,127686	7,158447
Limodorum abortivum	46,687708	11,114271
Limodorum abortivum	44,209144	5,277717
Limodorum abortivum	45,708025	7,269707
Limodorum abortivum	46,263974	7,42947
Limodorum abortivum	46,507501	11,329691
Limodorum abortivum	41,355903	14,316476
Limodorum abortivum	42,246625	9,066453
Limodorum abortivum	45,372183	5,943821
Limodorum abortivum	45,517545	6,496632
Limodorum abortivum	45,931062	11,111533
Limodorum abortivum	46,267646	7,877753
Limodorum abortivum	42,044543	14,180942
Limodorum abortivum	45,186716	5,479169
Limodorum abortivum	45,739204	10,706046
Limodorum abortivum	45,92637	10,821592
Limodorum abortivum	45,934928	10,889758
Limodorum abortivum	46,038466	7,141408
Limodorum abortivum	46,320295	7,779658
Limodorum abortivum	45,69201	10,758658
Limodorum abortivum	46,194992	7,026086
Limodorum abortivum	46,257419	11,248793
Limodorum abortivum	44,751284	5,494375
Limodorum abortivum	45,310792	6,36096
Limodorum abortivum	45,366413	6,310709
Limodorum abortivum	45,899654	11,346735
Limodorum abortivum	46,323009	7,58974
Limodorum abortivum	45,427962	6,645886
Limodorum abortivum	46,034299	11,104464
Limodorum abortivum	37,844927	14,016976
Limodorum abortivum	44,6811	6,016995
Limodorum abortivum	44,746137	5,439795
Limodorum abortivum	44,417267	6,519231
Limodorum abortivum	45,778082	11,473662
Limodorum abortivum	44,780073	6,018882
Limodorum abortivum	46,040048	11,454022
Limodorum abortivum	46,342803	11,301471
Limodorum abortivum	46,535895	11,245319
Limodorum abortivum	39,403677	21,092037
Limodorum abortivum	45,767594	6,397008
Limodorum abortivum	43,260375	17,089823
Limodorum abortivum	46,220967	7,255722
Limodorum abortivum	46,098652	7,054648
Limodorum abortivum	46,286385	13,147037
Limodorum abortivum	45,188872	5,671438

Limodorum abortivum	46,283446	7,027805
Limodorum abortivum	45,044198	6,812391
Limodorum abortivum	42,844776	12,765749
Limodorum abortivum	46,218585	11,114672
Limodorum abortivum	46,255463	11,164188
Limodorum abortivum	46,381286	10,959534
Limodorum abortivum	46,594544	11,157436
Limodorum abortivum	45,590279	6,208223
Limodorum abortivum	46,649481	10,992382
Limodorum abortivum	45,771788	10,993825
Limodorum abortivum	42,202633	13,290824
Limodorum abortivum	46,270297	7,553906
Limodorum abortivum	45,114207	7,267985
Limodorum abortivum	45,749327	7,528073
Limodorum abortivum	45,963017	10,651857
Limodorum abortivum	41,488972	13,932555
Limodorum abortivum	41,990715	14,134512
Limodorum abortivum	44,510559	7,149968
Limodorum abortivum	45,757305	10,817928
Limodorum abortivum	42,26667	9,11667
Limodorum abortivum	41,72	13,78
Limodorum abortivum	42,32	13,78
Limodorum abortivum	45,2	6,47
Limodorum abortivum	46,35	7,99
Limodorum abortivum	44,519423	34,228287
Limodorum abortivum	44,422675	34,012758
Limodorum abortivum	44,531044	34,158088
Limodorum abortivum	44,75547	34,45022
Limodorum abortivum	44,434297	34,058316
Limodorum abortivum	37,699828	26,623612
Limodorum abortivum	36,601887	30,484501
Limodorum abortivum	40,091698	22,489271
Limodorum abortivum	44,730866	34,316689
Limodorum abortivum	35,16	24,91
Limodorum abortivum	40,09	22,42
Limodorum abortivum	42,478016	-6,704696
Limodorum abortivum	42,992177	0,239391
Limodorum abortivum	43,033604	-0,102176
Limodorum abortivum	42,765082	1,842044
Limodorum abortivum	42,262407	1,532742
Limodorum abortivum	42,520989	2,986507
Limodorum abortivum	42,55807	2,2622
Limodorum abortivum	42,964432	0,584643
Limodorum abortivum	42,272773	-0,077182
Limodorum abortivum	36,537578	-5,210073
Limodorum abortivum	42,848656	2,17863

Limodorum abortivum	41,807501	2,461926
Limodorum abortivum	42,161414	1,094989
Limodorum abortivum	37,705372	-2,230063
Limodorum abortivum	42,625541	-0,313309
Limodorum abortivum	42,521024	1,218438
Limodorum abortivum	40,736312	-3,944498
Limodorum abortivum	42,2408	-0,2377
Limodorum abortivum	37,3	-3,94
Limodorum abortivum	37,85879081	-1,521122821
Limodorum abortivum	40,8388254	-3,7612171
Limodorum abortivum	40,88886962	0,388864334
Limodorum abortivum	42,015	2,524
Limodorum abortivum	42,034	0,965
Limodorum abortivum	42,165	1,288
Limodorum abortivum	42,24889154	-0,381176201
Limodorum abortivum	42,4488247	-6,961309022
Limodorum abortivum	43,14888785	-2,671240269
Limodorum abortivum	43,2788694	-4,63128175
Limodorum abortivum	43,29884154	-7,151330358
Limodorum abortivum	36,49	-5,63
Limodorum abortivum	36,81	-3,32
Limodorum abortivum	37,09	-3,56
Limodorum abortivum	39,79	2,88
Limodorum abortivum	42,62	-1,21
Limodorum abortivum	42,14514	8,99208
Limodorum abortivum	42,16624	9,22457
Limodorum abortivum	44,31828	6,17323
Limodorum abortivum	44,83151	5,90477
Limodorum abortivum	45,79607	6,14768
Limodorum abortivum	45,828674	13,971249
Limodorum abortivum	46,30447	6,02731
Limodorum abortivum	46,37338	6,12308
Limodorum abortivum	38,306438	16,071084
Limodorum abortivum	44,007389	10,29559
Limodorum abortivum	45,757913	9,770422
Limodorum abortivum	45,785183	10,046907
Limodorum abortivum	45,89171	11,06337
Limodorum abortivum	42,746779	12,816684
Limodorum abortivum	45,468994	6,545797
Limodorum abortivum	43,907689	7,488071
Limodorum abortivum	43,969178	7,35872
Limodorum abortivum	44,668342	6,601683
Limodorum abortivum	45,048767	5,376714
Limodorum abortivum	45,264725	5,739009
Limodorum abortivum	45,822032	9,278627
Limodorum abortivum	45,877679	10,863062

Limodorum abortivum	46,09399	6,39455
Limodorum abortivum	46,163648	6,205282
Limodorum abortivum	37,784562	20,77697
Limodorum abortivum	41,620905	9,182153
Limodorum abortivum	41,793628	13,426722
Limodorum abortivum	41,933726	13,20431
Limodorum abortivum	42,159492	14,051483
Limodorum abortivum	42,650355	12,7657
Limodorum abortivum	43,709933	6,967817
Limodorum abortivum	43,761285	7,126059
Limodorum abortivum	43,795288	6,22518
Limodorum abortivum	43,979668	6,709286
Limodorum abortivum	44,216604	6,268358
Limodorum abortivum	44,537792	6,443825
Limodorum abortivum	44,558718	5,878073
Limodorum abortivum	44,796552	5,319184
Limodorum abortivum	44,825795	5,58103
Limodorum abortivum	45,957189	16,005184
Limodorum abortivum	46,093786	6,560371
Limodorum abortivum	43,733319	10,50363
Limodorum abortivum	41,705625	9,04171
Limodorum abortivum	42,25216	8,767349
Limodorum abortivum	43,919362	10,599766
Limodorum abortivum	44,750848	9,353452
Limodorum abortivum	44,970913	5,616993
Limodorum abortivum	45,853198	5,643153
Limodorum abortivum	46,112627	11,111072
Limodorum abortivum	42,087762	12,895598
Limodorum abortivum	43,779256	11,818589
Limodorum abortivum	44,095497	6,843661
Limodorum abortivum	45,851396	5,798837
Limodorum abortivum	47,058936	7,038225
Limodorum abortivum	38,554121	14,847197
Limodorum abortivum	42,572212	9,315374
Limodorum abortivum	43,420144	13,011705
Limodorum abortivum	44,175268	5,711594
Limodorum abortivum	45,424851	6,028822
Limodorum abortivum	46,151292	5,8039
Limodorum abortivum	44,586978	4,168247
Limodorum abortivum	39,891262	20,20722
Limodorum abortivum	44,087778	7,592314
Limodorum abortivum	43,133275	12,999799
Limodorum abortivum	43,942077	7,088471
Limodorum abortivum	45,647361	11,183679
Limodorum abortivum	42,87139	9,354419
Limodorum abortivum	43,7276	6,865498

Limodorum abortivum	40,037336	9,650406
Limodorum abortivum	44,500562	7,203888
Limodorum abortivum	45,977258	12,445203
Limodorum abortivum	46,265067	11,351678
Limodorum abortivum	44,502616	6,736901
Limodorum abortivum	44,957557	5,172524
Limodorum abortivum	38,183504	16,041087
Limodorum abortivum	42,007539	14,031978
Limodorum abortivum	45,742304	11,375352
Limodorum abortivum	46,055267	10,962665
Limodorum abortivum	43,956873	6,856792
Limodorum abortivum	45,429722	5,863056
Limodorum abortivum	45,987247	9,266069
Limodorum abortivum	46,215658	6,319045
Limodorum abortivum	47,000421	6,932493
Limodorum abortivum	40,83489	15,01843
Limodorum abortivum	43,357315	12,717282
Limodorum abortivum	46,070292	11,611059
Limodorum abortivum	41,73615	9,288708
Limodorum abortivum	45,82562	9,623859
Limodorum abortivum	43,859965	7,715292
Limodorum abortivum	43,952815	7,17998
Limodorum abortivum	40,857462	9,155553
Limodorum abortivum	43,260343	16,637126
Limodorum abortivum	42,409027	13,332391
Limodorum abortivum	37,711092	15,112917
Limodorum abortivum	46,218281	7,304138
Limodorum abortivum	46,468929	11,530337
Limodorum abortivum	41,773583	13,849203
Limodorum abortivum	44,28824	10,699611
Limodorum abortivum	44,295934	7,956257
Limodorum abortivum	44,316658	15,478144
Limodorum abortivum	42,717238	9,441085
Limodorum abortivum	42,85954	11,578789
Limodorum abortivum	43,977047	7,5983
Limodorum abortivum	38,169855	14,944382
Limodorum abortivum	40,624912	14,529609
Limodorum abortivum	46,806549	6,549178
Limodorum abortivum	45,53323	5,943975
Limodorum abortivum	42,209584	14,172201
Limodorum abortivum	46,352423	11,094875
Limodorum abortivum	37,819389	15,102013
Limodorum abortivum	38,080242	20,719454
Limodorum abortivum	38,25948	20,662708
Limodorum abortivum	39,568645	20,733948
Limodorum abortivum	40,023407	20,459032

Limodorum abortivum	40,118122	20,80759
Limodorum abortivum	41,478745	13,887583
Limodorum abortivum	41,607082	13,976048
Limodorum abortivum	42,159679	19,192678
Limodorum abortivum	44,093189	7,830774
Limodorum abortivum	44,723427	5,541691
Limodorum abortivum	45,88242	10,754242
Limodorum abortivum	45,85568	9,045498
Limodorum abortivum	36,556349	5,865506
Limodorum abortivum	41,88333	9,08333
Limodorum abortivum	41,93333	8,9
Limodorum abortivum	42,46667	8,91667
Limodorum abortivum	37,92	13,96
Limodorum abortivum	40,98	21,07
Limodorum abortivum	41,96	13,82
Limodorum abortivum	43,67	18
Limodorum abortivum	41,923788	24,671755
Limodorum abortivum	36,893414	22,241857
Limodorum abortivum	35,026455	32,657906
Limodorum abortivum	34,93114	32,852788
Limodorum abortivum	36,370663	30,481458
Limodorum abortivum	37,85405	24,905293
Limodorum abortivum	35,151536	25,901478
Limodorum abortivum	44,566843	34,329498
Limodorum abortivum	36,90544	29,042883
Limodorum abortivum	36,263808	30,41478
Limodorum abortivum	36,549446	29,422877
Limodorum abortivum	38,737253	22,345232
Limodorum abortivum	44,82836	34,48691
Limodorum abortivum	38,168811	23,73606
Limodorum abortivum	36,71672	29,236108
Limodorum abortivum	37,155374	25,537192
Limodorum abortivum	35,05677	25,831837
Limodorum abortivum	35,136276	24,625711
Limodorum abortivum	36,796879	28,975317
Limodorum abortivum	38,573719	26,00333
Limodorum abortivum	37,95838	22,446456
Limodorum abortivum	44,478986	34,127411
Limodorum abortivum	41,891147	44,080983
Limodorum abortivum	38,68551	48,666344
Limodorum abortivum	39,58072	2,378743
Limodorum abortivum	40,271951	-0,000637
Limodorum abortivum	40,736371	0,368337
Limodorum abortivum	41,071332	-3,526015
Limodorum abortivum	42,20768	2,27548
Limodorum abortivum	42,277873	1,03252

Limodorum abortivum	42,475324	-6,831927
Limodorum abortivum	37,247684	-3,52401
Limodorum abortivum	40,842667	-4,028956
Limodorum abortivum	42,182062	0,053664
Limodorum abortivum	42,21982	0,759903
Limodorum abortivum	42,2742	3,216593
Limodorum abortivum	42,591115	2,364132
Limodorum abortivum	45,307927	3,467498
Limodorum abortivum	36,5075	-5,315556
Limodorum abortivum	38,349205	-2,735327
Limodorum abortivum	39,872719	2,986487
Limodorum abortivum	42,040883	1,752802
Limodorum abortivum	41,495641	-1,637338
Limodorum abortivum	43,446085	2,031177
Limodorum abortivum	36,098225	-5,657083
Limodorum abortivum	42,939788	1,801606
Limodorum abortivum	42,503168	2,650717
Limodorum abortivum	44,419972	3,211042
Limodorum abortivum	37,934076	-4,820125
Limodorum abortivum	41,20919	0,738
Limodorum abortivum	38,621094	-0,41175
Limodorum abortivum	42,246527	-7,182742
Limodorum abortivum	42,830515	2,585403
Limodorum abortivum	43,454451	2,870729
Limodorum abortivum	38,016471	-3,372705
Limodorum abortivum	44,198817	3,143784
Limodorum abortivum	44,301105	3,612928
Limodorum abortivum	38,498096	-2,421268
Limodorum abortivum	38,400697	-3,528487
Limodorum abortivum	42,820355	1,980982
Limodorum abortivum	38,755207	-9,425333
Limodorum abortivum	36,944431	-4,469023
Limodorum abortivum	44,457251	4,106416
Limodorum abortivum	36,761185	-5,332596
Limodorum abortivum	37,306335	-8,55787
Limodorum abortivum	42,989864	-4,449635
Limodorum abortivum	38,04	-6,26
Limodorum abortivum	36,742	-5,19
Limodorum abortivum	36,96876032	-3,011137119
Limodorum abortivum	37,3	-3,84
Limodorum abortivum	38,02878845	-2,061135932
Limodorum abortivum	38,16878552	-2,601148526
Limodorum abortivum	38,2	-2,8
Limodorum abortivum	38,27880208	-1,161122575
Limodorum abortivum	38,37877322	-4,171181605
Limodorum abortivum	38,49880767	-1,01112321

Limodorum abortivum	38,52	-0,67
Limodorum abortivum	39,16	-0,98
Limodorum abortivum	39,62	-0,97
Limodorum abortivum	39,717752	2,620815
Limodorum abortivum	40,04	-0,43
Limodorum abortivum	40,06884988	0,128873276
Limodorum abortivum	40,17883284	-1,761166286
Limodorum abortivum	40,18	-0,57
Limodorum abortivum	40,19879818	-5,091230599
Limodorum abortivum	40,29885633	0,298872771
Limodorum abortivum	40,37883454	-1,99117434
Limodorum abortivum	41,024	0,461
Limodorum abortivum	41,16879058	-7,581294807
Limodorum abortivum	41,1687968	-7,021284597
Limodorum abortivum	41,56890011	1,948884622
Limodorum abortivum	42,095	1,386
Limodorum abortivum	42,116	1,616
Limodorum abortivum	42,16886624	-2,611219732
Limodorum abortivum	42,25886985	-2,461218486
Limodorum abortivum	42,30887416	-2,161213456
Limodorum abortivum	42,311	1,405
Limodorum abortivum	42,33886574	-3,011230967
Limodorum abortivum	42,38889513	-0,341178023
Limodorum abortivum	42,67887156	-3,181241036
Limodorum abortivum	42,82889469	-1,341206984
Limodorum abortivum	42,71	-1,17
Limodorum abortivum	42,93	-1,88
Limodorum abortivum	44,65008	5,41622
Limodorum abortivum	44,80286	5,16093
Limodorum abortivum	40,519437	15,805268
Limodorum abortivum	43,336218	5,696418
Limodorum abortivum	44,51575	8,551912
Limodorum abortivum	44,630205	9,719722
Limodorum abortivum	45,479172	17,653769
Limodorum abortivum	45,941203	5,894658
Limodorum abortivum	37,373203	14,413314
Limodorum abortivum	40,53133	16,125607
Limodorum abortivum	41,754855	9,172047
Limodorum abortivum	42,302765	8,656426
Limodorum abortivum	42,533056	9,446667
Limodorum abortivum	42,698117	11,538936
Limodorum abortivum	44,413527	7,345373
Limodorum abortivum	44,418836	9,152525
Limodorum abortivum	44,579732	8,761153
Limodorum abortivum	44,975538	14,101689
Limodorum abortivum	45,003372	7,450266

Limodorum abortivum	45,884405	8,945845
Limodorum abortivum	45,905967	14,486297
Limodorum abortivum	47,134195	7,226883
Limodorum abortivum	39,341967	16,452504
Limodorum abortivum	40,745147	14,765311
Limodorum abortivum	41,927783	19,685405
Limodorum abortivum	43,77043	7,253478
Limodorum abortivum	44,206525	9,555258
Limodorum abortivum	44,38812	5,208358
Limodorum abortivum	44,425413	5,352367
Limodorum abortivum	45,455185	11,582327
Limodorum abortivum	45,492767	10,983922
Limodorum abortivum	38,387925	20,72443
Limodorum abortivum	41,916376	8,677616
Limodorum abortivum	42,257852	13,786481
Limodorum abortivum	45,53839	11,150456
Limodorum abortivum	38,012458	14,092537
Limodorum abortivum	42,358682	10,905134
Limodorum abortivum	42,986596	13,462496
Limodorum abortivum	43,311328	12,854337
Limodorum abortivum	43,607853	6,561064
Limodorum abortivum	43,846981	11,631069
Limodorum abortivum	43,863276	6,791103
Limodorum abortivum	43,955938	11,921875
Limodorum abortivum	44,124109	9,722959
Limodorum abortivum	45,84024	9,388168
Limodorum abortivum	39,6944	15,823773
Limodorum abortivum	41,306672	14,012472
Limodorum abortivum	42,073813	13,414465
Limodorum abortivum	42,9617	9,39915
Limodorum abortivum	46,521168	6,64723
Limodorum abortivum	43,808815	11,947368
Limodorum abortivum	43,898786	11,467281
Limodorum abortivum	45,74871	5,799492
Limodorum abortivum	38,824205	16,332158
Limodorum abortivum	40,119193	16,110823
Limodorum abortivum	40,329955	9,317661
Limodorum abortivum	42,451846	12,88912
Limodorum abortivum	42,790888	11,371014
Limodorum abortivum	45,900505	6,173647
Limodorum abortivum	39,404554	9,556722
Limodorum abortivum	41,779876	14,923414
Limodorum abortivum	41,993942	14,591217
Limodorum abortivum	44,531583	6,281186
Limodorum abortivum	44,374482	8,18077
Limodorum abortivum	44,532571	9,925165

Limodorum abortivum	44,839461	9,481665
Limodorum abortivum	38,51205	16,261712
Limodorum abortivum	42,726937	12,60518
Limodorum abortivum	44,843582	9,233789
Limodorum abortivum	40,177702	15,686838
Limodorum abortivum	44,525209	5,765274
Limodorum abortivum	40,091949	9,142737
Limodorum abortivum	43,087089	10,599514
Limodorum abortivum	45,750401	11,63266
Limodorum abortivum	39,913679	9,187637
Limodorum abortivum	40,702145	13,885044
Limodorum abortivum	45,655747	5,647419
Limodorum abortivum	46,958995	6,834512
Limodorum abortivum	39,742104	9,571816
Limodorum abortivum	44,114388	11,019612
Limodorum abortivum	43,900357	16,328815
Limodorum abortivum	42,346065	9,174851
Limodorum abortivum	37,918949	20,699312
Limodorum abortivum	43,580773	16,518849
Limodorum abortivum	45,351486	14,435554
Limodorum abortivum	36,775751	12,01201
Limodorum abortivum	43,093285	12,583983
Limodorum abortivum	44,419569	5,604452
Limodorum abortivum	41,703329	14,226364
Limodorum abortivum	47,74667	16,01167
Limodorum abortivum	42,09817	8,729811
Limodorum abortivum	38,464116	14,955931
Limodorum abortivum	42,624707	18,210478
Limodorum abortivum	44,805973	4,644728
Limodorum abortivum	45,986878	5,606209
Limodorum abortivum	47,988406	7,845702
Limodorum abortivum	37,901278	13,412676
Limodorum abortivum	44,457154	8,995242
Limodorum abortivum	43,374402	16,622142
Limodorum abortivum	44,102226	8,153554
Limodorum abortivum	39,195415	8,909862
Limodorum abortivum	39,389854	8,526957
Limodorum abortivum	39,861221	20,851694
Limodorum abortivum	39,91	20,461092
Limodorum abortivum	41,738976	14,000338
Limodorum abortivum	44,556389	5,165875
Limodorum abortivum	44,895974	14,348727
Limodorum abortivum	47,22611	15,23472
Limodorum abortivum	38,28	16,19
Limodorum abortivum	41,08	9,1
Limodorum abortivum	42,45	18,71

Limodorum abortivum	42,71	17,97
Limodorum abortivum	43,47	17,03
Limodorum abortivum	43,86	6,51
Limodorum abortivum	43,89	20,2
Limodorum abortivum	45,52	14,05
Limodorum abortivum	33,213694	35,755726
Limodorum abortivum	40,462561	32,623318
Limodorum abortivum	44,485475	33,639817
Limodorum abortivum	44,837942	34,360697
Limodorum abortivum	44,928509	35,075223
Limodorum abortivum	36,269287	29,715697
Limodorum abortivum	37,785872	26,7234
Limodorum abortivum	40,562958	29,82473
Limodorum abortivum	42,174783	22,949987
Limodorum abortivum	44,736107	37,417771
Limodorum abortivum	38,10425	23,880959
Limodorum abortivum	39,175414	23,888296
Limodorum abortivum	39,207402	26,053757
Limodorum abortivum	44,718383	37,92268
Limodorum abortivum	38,503157	27,331969
Limodorum abortivum	39,121127	23,724547
Limodorum abortivum	38,104131	27,104797
Limodorum abortivum	38,718944	27,166298
Limodorum abortivum	35,081658	33,014403
Limodorum abortivum	36,784632	28,206596
Limodorum abortivum	42,562192	22,981267
Limodorum abortivum	38,316112	27,105249
Limodorum abortivum	35,538162	36,123105
Limodorum abortivum	35,285252	33,534084
Limodorum abortivum	36,332001	33,855091
Limodorum abortivum	37,097668	25,440891
Limodorum abortivum	37,782314	28,589386
Limodorum abortivum	38,298294	26,065258
Limodorum abortivum	39,283615	26,206141
Limodorum abortivum	42,093128	23,118668
Limodorum abortivum	42,206574	22,347461
Limodorum abortivum	40,551226	21,381454
Limodorum abortivum	33,084254	35,182779
Limodorum abortivum	34,86	32,62
Limodorum abortivum	34,93	33
Limodorum abortivum	35,2	24,51
Limodorum abortivum	36,15	27,77
Limodorum abortivum	40,47	31,72
Limodorum abortivum	41,62	25,83
Limodorum abortivum	42,59	23,45
Limodorum abortivum	44,688057	38,544809

Limodorum abortivum	41,864796	46,095371
Limodorum abortivum	41,606602	44,540001
Limodorum abortivum	44,125112	43,027033
Limodorum abortivum	42,010935	47,966028
Limodorum abortivum	38,64	48,78
Limodorum abortivum	48,750703	19,095213
Limodorum abortivum	42,869802	-3,149452
Limodorum abortivum	42,973087	2,513844
Limodorum abortivum	44,3653	3,02853
Limodorum abortivum	40,508376	-0,240607
Limodorum abortivum	41,458244	1,661478
Limodorum abortivum	41,415329	1,914095
Limodorum abortivum	42,143713	0,224716
Limodorum abortivum	42,834175	2,762895
Limodorum abortivum	42,850844	-4,378403
Limodorum abortivum	43,903186	3,026988
Limodorum abortivum	40,891217	-0,071855
Limodorum abortivum	42,99077	1,544484
Limodorum abortivum	43,090115	1,34625
Limodorum abortivum	43,907736	3,746256
Limodorum abortivum	38,345695	-1,970913
Limodorum abortivum	38,835495	-0,832603
Limodorum abortivum	40,338654	-3,122857
Limodorum abortivum	40,518416	0,170416
Limodorum abortivum	41,443862	1,039972
Limodorum abortivum	42,219667	-0,759862
Limodorum abortivum	42,428295	0,109837
Limodorum abortivum	42,945045	2,326322
Limodorum abortivum	42,999025	-3,551292
Limodorum abortivum	37,906368	-6,72741
Limodorum abortivum	37,95002	-5,622608
Limodorum abortivum	41,168878	1,047092
Limodorum abortivum	41,702431	2,497617
Limodorum abortivum	39,560942	-1,801915
Limodorum abortivum	40,812122	-0,304857
Limodorum abortivum	41,95379	1,466722
Limodorum abortivum	41,986647	1,912582
Limodorum abortivum	44,057887	1,751361
Limodorum abortivum	37,926563	-5,809613
Limodorum abortivum	38,922792	-7,660976
Limodorum abortivum	39,82832	-6,030589
Limodorum abortivum	42,510513	3,112825
Limodorum abortivum	42,756519	-2,913978
Limodorum abortivum	38,686897	-0,617757
Limodorum abortivum	39,85081	-1,12101
Limodorum abortivum	39,901611	-6,714997

Limodorum abortivum	40,533736	-2,698928
Limodorum abortivum	41,016899	-2,995004
Limodorum abortivum	41,938986	0,214203
Limodorum abortivum	42,15013	-0,418382
Limodorum abortivum	42,706848	-2,068318
Limodorum abortivum	42,747605	-2,441326
Limodorum abortivum	44,26134	4,14808
Limodorum abortivum	44,496801	3,470911
Limodorum abortivum	44,628755	2,213477
Limodorum abortivum	40,290692	-4,377194
Limodorum abortivum	41,919382	1,196566
Limodorum abortivum	39,896616	-0,526518
Limodorum abortivum	41,334722	1,320252
Limodorum abortivum	44,216306	3,292782
Limodorum abortivum	45,727125	3,190699
Limodorum abortivum	41,72777	1,77647
Limodorum abortivum	41,987878	0,431362
Limodorum abortivum	42,864816	-1,730474
Limodorum abortivum	45,110261	2,873715
Limodorum abortivum	44,851707	1,813757
Limodorum abortivum	41,031667	0,608911
Limodorum abortivum	38,824904	-0,05706
Limodorum abortivum	41,79765	2,968403
Limodorum abortivum	38,628417	-7,06981
Limodorum abortivum	40,931367	0,252611
Limodorum abortivum	36,341657	4,112767
Limodorum abortivum	40,619606	-2,275542
Limodorum abortivum	36,271402	-5,396383
Limodorum abortivum	40,19959	-0,191937
Limodorum abortivum	37,252388	-8,116168
Limodorum abortivum	39,463825	-8,902589
Limodorum abortivum	43,875026	2,651025
Limodorum abortivum	42,383538	-1,23879
Limodorum abortivum	39,729798	3,432806
Limodorum abortivum	44,6515	2,016519
Limodorum abortivum	47,422963	3,738509
Limodorum abortivum	37,888842	-6,546251
Limodorum abortivum	41,835514	-1,761052
Limodorum abortivum	40,03	-1,96
Limodorum abortivum	40	-1,66
Limodorum abortivum	38,530443	-8,018122
Limodorum abortivum	41,018222	-3,247929
Limodorum abortivum	36,498173	-4,774802
Limodorum abortivum	38,011491	-6,096302
Limodorum abortivum	33,89	-4,42
Limodorum abortivum	37,94879852	-0,911112431

Limodorum abortivum	38,167	-5,163
Limodorum abortivum	38,44877915	-3,721174348
Limodorum abortivum	38,45	-3,22
Limodorum abortivum	38,70879559	-2,591157243
Limodorum abortivum	38,74	0,17
Limodorum abortivum	38,95	-0,37
Limodorum abortivum	39,04	-0,81
Limodorum abortivum	39,15	-1,14
Limodorum abortivum	39,36	-1,12
Limodorum abortivum	39,44	-0,92
Limodorum abortivum	39,48	-1,39
Limodorum abortivum	39,59	-1,13
Limodorum abortivum	39,81	-0,33
Limodorum abortivum	39,93882023	-2,511176736
Limodorum abortivum	40,12	-1,29
Limodorum abortivum	40,30884536	-0,801149531
Limodorum abortivum	40,4	-1,62
Limodorum abortivum	40,57881835	-3,92121539
Limodorum abortivum	40,57882671	-3,131200176
Limodorum abortivum	40,61885986	-0,011139061
Limodorum abortivum	40,62883468	-2,471188229
Limodorum abortivum	41,13882878	-4,03122788
Limodorum abortivum	41,17	-1,08
Limodorum abortivum	41,18880218	-6,571276708
Limodorum abortivum	41,52884188	-3,581226544
Limodorum abortivum	41,752	2,189
Limodorum abortivum	41,76885317	-3,011219965
Limodorum abortivum	41,786	1,996
Limodorum abortivum	41,827	0,96
Limodorum abortivum	41,886	0,765
Limodorum abortivum	41,89885064	-3,51123226
Limodorum abortivum	41,899	-4,392
Limodorum abortivum	42,36888841	-0,941189938
Limodorum abortivum	42,43887474	-2,381220382
Limodorum abortivum	42,473	-1,425
Limodorum abortivum	42,72886889	-3,531248954
Limodorum abortivum	42,92887083	-3,77125771
Limodorum abortivum	42,93887574	-3,341249411
Limodorum abortivum	43,33888913	-2,961249944
Limodorum abortivum	37,53	-6,35
Limodorum abortivum	37,99	-8,7
Limodorum abortivum	39,44	3,17
Limodorum abortivum	40,33	-1,37
Limodorum abortivum	40,82	-2,04
Limodorum abortivum	42,07	-1,34
Limodorum abortivum	43,39	-4,53

Limodorum abortivum	45,14	2,03
Limodorum abortivum	43,43984	5,50947
Limodorum abortivum	43,72309	5,42975
Limodorum abortivum	44,44314	8,040093
Limodorum abortivum	44,82026	5,68901
Limodorum abortivum	42,763259	10,365038
Limodorum abortivum	43,457586	6,89854
Limodorum abortivum	44,262605	19,824058
Limodorum abortivum	44,731934	5,024751
Limodorum abortivum	46,960158	17,864108
Limodorum abortivum	41,690091	9,382889
Limodorum abortivum	43,676613	5,138457
Limodorum abortivum	40,918563	9,309548
Limodorum abortivum	42,782987	17,350415
Limodorum abortivum	43,448534	6,586841
Limodorum abortivum	43,538777	5,996828
Limodorum abortivum	43,603675	16,718583
Limodorum abortivum	43,963382	5,751267
Limodorum abortivum	44,256082	9,404239
Limodorum abortivum	44,627547	8,13313
Limodorum abortivum	44,679298	8,366448
Limodorum abortivum	45,07755	14,595465
Limodorum abortivum	45,54293	11,405465
Limodorum abortivum	45,945154	12,200283
Limodorum abortivum	46,672996	6,483617
Limodorum abortivum	47,445255	8,043687
Limodorum abortivum	47,568306	5,130509
Limodorum abortivum	47,698963	7,515645
Limodorum abortivum	48,313074	5,201097
Limodorum abortivum	39,435897	19,917638
Limodorum abortivum	43,238167	5,39423
Limodorum abortivum	43,25908	12,525108
Limodorum abortivum	43,71091	11,166432
Limodorum abortivum	44,050828	5,341113
Limodorum abortivum	44,159686	5,968878
Limodorum abortivum	44,292833	11,001744
Limodorum abortivum	44,351658	11,290988
Limodorum abortivum	46,778891	17,298437
Limodorum abortivum	47,090557	4,680437
Limodorum abortivum	47,185572	4,920325
Limodorum abortivum	47,524498	18,940806
Limodorum abortivum	41,475141	8,984077
Limodorum abortivum	43,169671	5,700475
Limodorum abortivum	43,391958	11,439998
Limodorum abortivum	43,977313	12,420097
Limodorum abortivum	44,219477	11,780999

Limodorum abortivum	45,805928	11,928251
Limodorum abortivum	42,218825	12,04166
Limodorum abortivum	43,913657	8,059362
Limodorum abortivum	43,31188	11,079795
Limodorum abortivum	43,930202	12,119758
Limodorum abortivum	44,522365	6,044583
Limodorum abortivum	45,78524	5,440771
Limodorum abortivum	42,471108	12,261463
Limodorum abortivum	43,250928	10,778014
Limodorum abortivum	44,038666	6,158437
Limodorum abortivum	44,251803	11,528122
Limodorum abortivum	45,56138	10,195872
Limodorum abortivum	48,008149	16,221955
Limodorum abortivum	42,64725	9,02952
Limodorum abortivum	43,524239	12,897893
Limodorum abortivum	44,578924	4,325258
Limodorum abortivum	45,107722	5,73583
Limodorum abortivum	45,54595	13,753108
Limodorum abortivum	45,667594	13,989498
Limodorum abortivum	45,781589	13,653447
Limodorum abortivum	46,31901	4,81157
Limodorum abortivum	43,313782	6,038968
Limodorum abortivum	43,871788	12,912708
Limodorum abortivum	43,000586	13,831956
Limodorum abortivum	43,633084	12,456209
Limodorum abortivum	44,063417	5,550125
Limodorum abortivum	48,127985	16,927872
Limodorum abortivum	43,374832	17,591378
Limodorum abortivum	44,44605	10,440214
Limodorum abortivum	47,330763	8,473696
Limodorum abortivum	45,874104	18,297269
Limodorum abortivum	43,278164	13,338136
Limodorum abortivum	45,909394	6,035134
Limodorum abortivum	47,760379	8,839057
Limodorum abortivum	44,813728	9,077262
Limodorum abortivum	44,874062	15,59691
Limodorum abortivum	43,448302	11,785073
Limodorum abortivum	44,261531	20,601555
Limodorum abortivum	44,733284	10,021842
Limodorum abortivum	43,042262	9,841026
Limodorum abortivum	45,089265	5,162676
Limodorum abortivum	42,874783	12,478962
Limodorum abortivum	39,702139	8,868323
Limodorum abortivum	40,284709	14,962077
Limodorum abortivum	40,813918	9,6404
Limodorum abortivum	45,272386	5,368959

Limodorum abortivum	47,105	15,38306
Limodorum abortivum	40,512842	9,824564
Limodorum abortivum	43,363561	12,326634
Limodorum abortivum	47,421079	8,693147
Limodorum abortivum	44,199296	8,416534
Limodorum abortivum	40,637215	17,225624
Limodorum abortivum	42,999243	10,851114
Limodorum abortivum	44,486458	10,911149
Limodorum abortivum	37,096752	14,383767
Limodorum abortivum	39,956299	9,44058
Limodorum abortivum	42,679153	21,193314
Limodorum abortivum	47,724113	9,123961
Limodorum abortivum	48,184708	7,82279
Limodorum abortivum	42,748937	11,93241
Limodorum abortivum	41,4397	13,612704
Limodorum abortivum	37,655076	20,82076
Limodorum abortivum	47,0725	15,07917
Limodorum abortivum	40,557954	14,262402
Limodorum abortivum	46,18	5,41
Limodorum abortivum	43,390273	28,061514
Limodorum abortivum	44,691195	33,881057
Limodorum abortivum	31,774276	35,088888
Limodorum abortivum	44,8224	34,0661
Limodorum abortivum	39,197303	26,457627
Limodorum abortivum	40,393257	37,804403
Limodorum abortivum	44,718478	37,555125
Limodorum abortivum	44,788012	34,635902
Limodorum abortivum	33,866525	35,5405
Limodorum abortivum	39,679204	32,935506
Limodorum abortivum	41,02692	26,145486
Limodorum abortivum	44,497975	33,782204
Limodorum abortivum	44,688847	34,427232
Limodorum abortivum	44,81997	34,852826
Limodorum abortivum	38,225386	27,441892
Limodorum abortivum	38,401203	26,7282
Limodorum abortivum	44,473275	38,156253
Limodorum abortivum	36,18	27,92
Limodorum abortivum	42,806937	27,853624
Limodorum abortivum	41,055053	35,591093
Limodorum abortivum	40,16201	23,728103
Limodorum abortivum	37,661205	26,878574
Limodorum abortivum	32,998772	35,393985
Limodorum abortivum	44,760872	38,123613
Limodorum abortivum	44,896748	37,932964
Limodorum abortivum	36,403605	28,147163
Limodorum abortivum	36,273023	29,305462

Limodorum abortivum	35,647246	35,909454
Limodorum abortivum	36,485722	29,31551
Limodorum abortivum	36,527313	30,54682
Limodorum abortivum	36,54826	33,940544
Limodorum abortivum	37,383877	28,2033
Limodorum abortivum	39,018452	26,180592
Limodorum abortivum	41,43119	25,653534
Limodorum abortivum	44,8437	28,697662
Limodorum abortivum	32,00576	35,135412
Limodorum abortivum	32,384447	35,060734
Limodorum abortivum	32,670254	35,026163
Limodorum abortivum	35,98	27,88
Limodorum abortivum	36,3	22,96
Limodorum abortivum	37,11	30,92
Limodorum abortivum	41,53	25,21
Limodorum abortivum	41,62	26,02
Limodorum abortivum	42,03	26,42
Limodorum abortivum	45,17	28,46
Limodorum abortivum	44,454462	38,350013
Limodorum abortivum	44,31974	38,708243
Limodorum abortivum	44,64115	39,05271
Limodorum abortivum	43,415981	39,995547
Limodorum abortivum	44,110869	39,06587
Limodorum abortivum	44,825171	38,389248
Limodorum abortivum	49,289722	3,779167
Limodorum abortivum	48,906155	6,006733
Limodorum abortivum	48,671012	17,897334
Limodorum abortivum	48,459529	5,651753
Limodorum abortivum	49,789951	6,553066
Limodorum abortivum	51,380119	8,574961
Limodorum abortivum	50,095496	4,668513
Limodorum abortivum	48,891357	5,654182
Limodorum abortivum	49,412872	6,707067
Limodorum abortivum	51,01643	10,20371
Limodorum abortivum	48,69	5,74
Limodorum abortivum	39,526789	-0,511401
Limodorum abortivum	43,71108	3,973482
Limodorum abortivum	44,37727	0,99742
Limodorum abortivum	44,6704	1,54503
Limodorum abortivum	44,93526	1,02202
Limodorum abortivum	45,01179	1,48946
Limodorum abortivum	45,2154	1,13273
Limodorum abortivum	45,22081	0,83703
Limodorum abortivum	45,92676	0,01815
Limodorum abortivum	43,921348	2,200088
Limodorum abortivum	45,50882	0,058275

Limodorum abortivum	39,733074	3,226927
Limodorum abortivum	43,251145	2,709798
Limodorum abortivum	46,0191	-0,52263
Limodorum abortivum	39,231853	-8,698583
Limodorum abortivum	42,150619	2,996508
Limodorum abortivum	43,232862	2,937237
Limodorum abortivum	43,474524	3,536143
Limodorum abortivum	44,099393	1,36133
Limodorum abortivum	44,962693	0,630608
Limodorum abortivum	45,467346	0,38263
Limodorum abortivum	47,151988	0,408587
Limodorum abortivum	36,38172	-6,185538
Limodorum abortivum	36,938075	-1,93757
Limodorum abortivum	38,660043	-9,167137
Limodorum abortivum	42,817215	3,034976
Limodorum abortivum	43,499928	-3,585148
Limodorum abortivum	44,425089	1,500104
Limodorum abortivum	44,443583	1,902384
Limodorum abortivum	44,71462	0,59978
Limodorum abortivum	45,711128	-0,859383
Limodorum abortivum	45,830999	0,288144
Limodorum abortivum	46,572886	0,854991
Limodorum abortivum	47,2066	-0,023153
Limodorum abortivum	47,53473	3,46649
Limodorum abortivum	47,677302	-0,232589
Limodorum abortivum	48,280175	2,427758
Limodorum abortivum	43,53483	0,667897
Limodorum abortivum	44,920375	-0,237658
Limodorum abortivum	40,913213	-2,742728
Limodorum abortivum	41,700513	1,429937
Limodorum abortivum	47,775956	3,822673
Limodorum abortivum	46,738161	3,171742
Limodorum abortivum	43,16998	0,854852
Limodorum abortivum	43,443878	3,165595
Limodorum abortivum	45,69547	-0,39472
Limodorum abortivum	46,913525	0,797188
Limodorum abortivum	46,95247	2,283876
Limodorum abortivum	46,971143	1,957218
Limodorum abortivum	48,01966	2,12715
Limodorum abortivum	48,33601	2,102138
Limodorum abortivum	42,583632	-6,681965
Limodorum abortivum	43,176934	2,293304
Limodorum abortivum	39,575625	2,657585
Limodorum abortivum	41,129892	1,338287
Limodorum abortivum	38,813291	-8,805253
Limodorum abortivum	37,142539	-6,541141

Limodorum abortivum	41,644162	-0,905553
Limodorum abortivum	46,610278	0,605278
Limodorum abortivum	38,964892	-8,079005
Limodorum abortivum	48,316615	2,929712
Limodorum abortivum	39,482902	-8,59439
Limodorum abortivum	44,28223	0,704439
Limodorum abortivum	39,982752	4,130784
Limodorum abortivum	47,908743	4,079204
Limodorum abortivum	42,579706	2,812931
Limodorum abortivum	43,62601	2,176166
Limodorum abortivum	37,238905	-7,087697
Limodorum abortivum	45,064574	-0,570281
Limodorum abortivum	39,171069	-7,832709
Limodorum abortivum	44,369242	2,594133
Limodorum abortivum	47,218464	-1,728319
Limodorum abortivum	44,649295	-1,252111
Limodorum abortivum	48,292669	4,044464
Limodorum abortivum	46,42337	0,21059
Limodorum abortivum	42,005356	2,286056
Limodorum abortivum	42,86039	-2,642293
Limodorum abortivum	41,799682	2,731071
Limodorum abortivum	43,913911	1,106195
Limodorum abortivum	38,522082	-8,838784
Limodorum abortivum	47,2694	2,7541
Limodorum abortivum	43,88819	3,31858
Limodorum abortivum	38,02876671	-4,191176161
Limodorum abortivum	39,53	-2,57
Limodorum abortivum	39,79882344	-1,931162995
Limodorum abortivum	39,98883965	-0,721142392
Limodorum abortivum	40,07	-2,22
Limodorum abortivum	40,21	-3,93
Limodorum abortivum	40,33881971	-3,331199703
Limodorum abortivum	40,47886118	0,408871898
Limodorum abortivum	40,72886765	0,528869998
Limodorum abortivum	41,43883501	-4,041233741
Limodorum abortivum	41,50883165	-4,49124373
Limodorum abortivum	41,572	2,203
Limodorum abortivum	41,75884372	-3,871236574
Limodorum abortivum	41,96888713	-0,20116727
Limodorum abortivum	42,33886035	-3,511240827
Limodorum abortivum	42,46887016	-2,871230737
Limodorum abortivum	42,56886256	-3,781250678
Limodorum abortivum	43,1257	1,91774
Limodorum abortivum	37,49	-6,84
Limodorum abortivum	38,4	-8,54
Limodorum abortivum	40,79	-3,62

Limodorum abortivum	42,49	-1,72
Limodorum abortivum	44,63	0,85
Limodorum abortivum	44,69	0,14
Limodorum abortivum	46,63	1,09
Limodorum abortivum	43,87398	4,713
Limodorum abortivum	47,75937	4,82048
Limodorum abortivum	43,775332	15,749982
Limodorum abortivum	43,079048	6,023332
Limodorum abortivum	43,466964	5,004947
Limodorum abortivum	44,386443	4,889593
Limodorum abortivum	43,152384	11,575791
Limodorum abortivum	43,312255	10,535023
Limodorum abortivum	43,491669	5,322352
Limodorum abortivum	43,772852	4,244559
Limodorum abortivum	44,055206	4,192943
Limodorum abortivum	44,857568	12,249179
Limodorum abortivum	45,280072	13,761513
Limodorum abortivum	45,555793	10,571103
Limodorum abortivum	43,855749	10,260876
Limodorum abortivum	44,048073	5,080438
Limodorum abortivum	48,19306	16,480927
Limodorum abortivum	43,376389	6,389167
Limodorum abortivum	44,114168	4,50185
Limodorum abortivum	46,915798	6,267617
Limodorum abortivum	40,123952	18,448599
Limodorum abortivum	45,012983	4,937344
Limodorum abortivum	46,179122	6,024112
Limodorum abortivum	45,9316	4,953189
Limodorum abortivum	43,56502	5,746585
Limodorum abortivum	43,811812	6,135215
Limodorum abortivum	46,134826	13,035297
Limodorum abortivum	47,320258	5,101746
Limodorum abortivum	47,87815	4,40193
Limodorum abortivum	48,13567	4,59947
Limodorum abortivum	45,456669	10,806218
Limodorum abortivum	39,217347	8,462396
Limodorum abortivum	43,599933	10,777948
Limodorum abortivum	45,687628	12,254224
Limodorum abortivum	39,927495	9,696394
Limodorum abortivum	44,385865	7,538183
Limodorum abortivum	39,772076	8,579253
Limodorum abortivum	46,759708	16,913706
Limodorum abortivum	45,725376	13,402657
Limodorum abortivum	42,227813	14,548237
Limodorum abortivum	43,008584	11,948118
Limodorum abortivum	47,96814	7,697787

Limodorum abortivum	42,737292	10,967956
Limodorum abortivum	44,557201	14,442673
Limodorum abortivum	43,097429	6,353653
Limodorum abortivum	42,011223	9,461792
Limodorum abortivum	39,238846	9,112097
Limodorum abortivum	41,7229	12,308141
Limodorum abortivum	45,900849	5,196265
Limodorum abortivum	46,616302	5,011657
Limodorum abortivum	47,819093	16,238508
Limodorum abortivum	40,952435	8,224619
Limodorum abortivum	41,243771	9,144788
Limodorum abortivum	47,514847	8,50194
Limodorum abortivum	42,27	19,14
Limodorum abortivum	41,105516	29,030264
Limodorum abortivum	40,862409	29,124661
Limodorum abortivum	38,15417	21,36917
Limodorum abortivum	42,295903	27,757337
Limodorum abortivum	38,178845	26,771553
Limodorum abortivum	32,095485	34,826488
Limodorum abortivum	36,874786	30,642508
Limodorum abortivum	44,694004	33,615245
Limodorum abortivum	41,99	28,02
Limodorum abortivum	45,35	29,54
Limodorum abortivum	41,874758	48,522253
Limodorum abortivum	49,386093	2,23826
Limodorum abortivum	49,403164	3,277992
Limodorum abortivum	50,733807	3,680668
Limodorum abortivum	48,636138	-4,416463
Limodorum abortivum	49,039977	1,365353
Limodorum abortivum	49,117233	1,840758
Limodorum abortivum	49,151386	2,199955
Limodorum abortivum	49,536571	2,806721
Limodorum abortivum	48,801083	1,770268
Limodorum abortivum	48,833229	2,418347
Limodorum abortivum	48,769778	0,267111
Limodorum abortivum	49,304858	2,755748
Limodorum abortivum	49,518	3,727
Limodorum abortivum	49,435423	3,969269
Limodorum abortivum	48,69985	5,3817
Limodorum abortivum	48,777809	7,84461
Limodorum abortivum	49,204251	7,400176
Limodorum abortivum	49,123974	6,21908
Limodorum abortivum	51,934411	7,622346
Limodorum abortivum	50,606713	4,542614
Limodorum abortivum	48,957409	9,204166
Limodorum abortivum	52,357143	9,8282

Limodorum abortivum	49,163604	5,388447
Limodorum abortivum	49,362413	6,193738
Limodorum abortivum	50,588463	4,316683
Limodorum abortivum	49,074997	7,117233
Limodorum abortivum	49,089211	8,486383
Limodorum abortivum	50,06	4,18
Rhodanthidium_septemdentatum	7,5359	45,7683
Rhodanthidium_septemdentatum	10,030324	45,682518
Rhodanthidium_septemdentatum	11,644236	46,706207
Rhodanthidium_septemdentatum	20,135341	40,074666
Rhodanthidium_septemdentatum	20,8071	41,0089
Rhodanthidium_septemdentatum	10,998852	45,826582
Rhodanthidium_septemdentatum	12,704749	43,02051
Rhodanthidium_septemdentatum	6,62111	44,38074
Rhodanthidium_septemdentatum	9,540328	40,193972
Rhodanthidium_septemdentatum	8,904	42,395
Rhodanthidium_septemdentatum	22,36598	37,07168
Rhodanthidium_septemdentatum	8,98064	45,903728
Rhodanthidium_septemdentatum	7,01706	46,27113
Rhodanthidium_septemdentatum	2,041742	41,494943
Rhodanthidium_septemdentatum	16,353346	48,276245
Rhodanthidium_septemdentatum	14,544664	42,196217
Rhodanthidium_septemdentatum	6,009155	43,194032
Rhodanthidium_septemdentatum	3,67081	43,489361
Rhodanthidium_septemdentatum	14,47588	45,326854
Rhodanthidium_septemdentatum	20,554281	38,138206
Rhodanthidium_septemdentatum	2,785114	39,780392
Rhodanthidium_septemdentatum	4,032351	43,726989
Rhodanthidium_septemdentatum	5,98769	43,939175
Rhodanthidium_septemdentatum	2,879992	42,194443
Rhodanthidium_septemdentatum	6,292287	43,371012
Rhodanthidium_septemdentatum	5,156449	44,18742
Rhodanthidium_septemdentatum	15,521875	46,65487
Rhodanthidium_septemdentatum	23,979789	38,102069
Rhodanthidium_septemdentatum	6,935836	43,551728
Rhodanthidium_septemdentatum	13,859235	44,839535
Rhodanthidium_septemdentatum	16,408293	40,980624
Rhodanthidium_septemdentatum	2,472988	42,177209
Rhodanthidium_septemdentatum	10,421618	42,866441
Rhodanthidium_septemdentatum	10,367505	43,570243
Rhodanthidium_septemdentatum	4,553835	44,305415
Rhodanthidium_septemdentatum	10,984153	45,482766
Rhodanthidium_septemdentatum	3,163459	39,76333
Rhodanthidium_septemdentatum	5,558744	43,88985
Rhodanthidium_septemdentatum	9,177792	41,238085
Rhodanthidium_septemdentatum	9,22865	41,529

Rhodanthidium_septemdentatum	9,39231	41,92377
Rhodanthidium_septemdentatum	3,273461	42,291492
Rhodanthidium_septemdentatum	9,06846	42,66107
Rhodanthidium_septemdentatum	6,610222	43,235936
Rhodanthidium_septemdentatum	15,288067	43,879425
Rhodanthidium_septemdentatum	4,703121	44,003943
Rhodanthidium_septemdentatum	6,474985	44,089302
Rhodanthidium_septemdentatum	4,147182	44,192607
Rhodanthidium_septemdentatum	20,68118	38,82635
Rhodanthidium_septemdentatum	15,701425	48,484875
Rhodanthidium_septemdentatum	26,353662	39,073195
Rhodanthidium_septemdentatum	24,095198	41,13998
Rhodanthidium_septemdentatum	5,665882	43,354184
Rhodanthidium_septemdentatum	13,404939	43,596213
Rhodanthidium_septemdentatum	3,769838	43,826388
Rhodanthidium_septemdentatum	4,856523	43,759822
Rhodanthidium_septemdentatum	9,287232	44,331088
Rhodanthidium_septemdentatum	26,612908	39,019262
Rhodanthidium_septemdentatum	2,297208	41,512061
Rhodanthidium_septemdentatum	17,316273	40,36904
Rhodanthidium_septemdentatum	7,663027	43,878547
Rhodanthidium_septemdentatum	12,123058	44,119715
Rhodanthidium_septemdentatum	11,335786	43,312894
Rhodanthidium_septemdentatum	3,177477	39,446001
Rhodanthidium_septemdentatum	3,087869	43,13303
Rhodanthidium_septemdentatum	2,696563	43,268932
Rhodanthidium_septemdentatum	10,650112	43,299172
Rhodanthidium_septemdentatum	2,159103	43,360297
Rhodanthidium_septemdentatum	3,415546	44,190525
Rhodanthidium_septemdentatum	5,789843	44,503178
Rhodanthidium_septemdentatum	-5,601303	36,228103
Rhodanthidium_septemdentatum	21,375135	40,534322
Rhodanthidium_septemdentatum	16,60665	40,663891
Rhodanthidium_septemdentatum	2,034495	42,048517
Rhodanthidium_septemdentatum	21,90019	37,4297
Rhodanthidium_septemdentatum	11,056505	43,461125
Rhodanthidium_septemdentatum	11,21424	46,103227
Rhodanthidium_septemdentatum	-8,268108	41,069349
Rhodanthidium_septemdentatum	3,22313	43,472605
Rhodanthidium_septemdentatum	22,755674	37,731121
Rhodanthidium_septemdentatum	9,53	42,373
Rhodanthidium_septemdentatum	15,052793	37,504397
Rhodanthidium_septemdentatum	12,246503	44,753273
Rhodanthidium_septemdentatum	21,9197	37,17618
Rhodanthidium_septemdentatum	-9,326655	38,69686
Rhodanthidium_septemdentatum	23,228276	39,174863

Rhodanthidium_septemdentatum	46,032149	45,859627
Rhodanthidium_septemdentatum	35,32889	37,00167
Rhodanthidium_septemdentatum	3,20151	41,91497
Rhodanthidium_septemdentatum	16,45583	43,51389
Rhodanthidium_septemdentatum	14,77417	44,76944
Rhodanthidium_septemdentatum	14,61694	45,07528
Rhodanthidium_septemdentatum	14,23706	50,87411
Rhodanthidium_septemdentatum	7,417	43,872
Rhodanthidium_septemdentatum	-9,404093	39,362395
Rhodanthidium_septemdentatum	16,05	47,83
Rhodanthidium_septemdentatum	15,39	48,36
Rhodanthidium_septemdentatum	8,2873	40,5946
Rhodanthidium_septemdentatum	3,430195	39,742645
Rhodanthidium_septemdentatum	-8,802153	37,922437
Rhodanthidium_septemdentatum	24,899611	41,89715
Rhodanthidium_septemdentatum	10,0336	44,6454
Rhodanthidium_septemdentatum	2,7498	39,4953
Russula chloroides	43,031173	-2,077863
Russula chloroides	43,23099	-4,738159
Russula chloroides	41,813368	-1,809304
Russula chloroides	42,573074	0,511665
Russula chloroides	42,643084	0,569384
Russula chloroides	42,52587	0,381495
Russula chloroides	42,46	1,23
Russula chloroides	42,59	1,06
Russula chloroides	42,62	1,34
Russula chloroides	42,82	0,74
Russula chloroides	44,1958	7,49363
Russula chloroides	45,08743	5,85015
Russula chloroides	47,27	12,47
Russula chloroides	47,02	13,2
Russula chloroides	46,183548	9,677075
Russula chloroides	47,23	12,27
Russula chloroides	62,285855	7,206375
Russula chloroides	62,461352	8,188288
Russula chloroides	62,545401	8,186626
Russula chloroides	60,5659	6,9705
Russula chloroides	61,4462	7,4706
Russula chloroides	62,224505	7,433676
Russula chloroides	62,296368	7,309394
Russula chloroides	61,211497	7,086391
Russula chloroides	61,158191	8,526162
Russula chloroides	42,30971	-2,245268
Russula chloroides	40,84388	-3,770229
Russula chloroides	41,204438	-3,468855
Russula chloroides	42,518685	-0,15731

Russula chloroides	42,704363	-0,328238
Russula chloroides	42,836313	-0,708453
Russula chloroides	42,662216	-1,142008
Russula chloroides	42,79695	-0,555436
Russula chloroides	42,31	0,66
Russula chloroides	42,41	1,29
Russula chloroides	42,68	0,88
Russula chloroides	42,978	-2,393
Russula chloroides	43,158	-3,597
Russula chloroides	42,433371	9,35915
Russula chloroides	47,35	12,59
Russula chloroides	44,37347	9,623809
Russula chloroides	47	13,12
Russula chloroides	62,253979	8,20841
Russula chloroides	60,258062	8,625785
Russula chloroides	62,303496	7,108108
Russula chloroides	62,889421	7,351139
Russula chloroides	62,566616	9,119158
Russula chloroides	62,410605	6,094899
Russula chloroides	61,200615	6,840431
Russula chloroides	61,306268	7,255222
Russula chloroides	62,60565	9,704204
Russula chloroides	60,508737	6,90577
Russula chloroides	61,5404	8,9385
Russula chloroides	40,638966	-3,906994
Russula chloroides	42,908894	-2,146565
Russula chloroides	42,989167	-1,457778
Russula chloroides	39,4571	-5,49157
Russula chloroides	36,75	-5,33
Russula chloroides	38,65	-0,55
Russula chloroides	38,87	-0,37
Russula chloroides	39,89	-0,33
Russula chloroides	40,04	0,005
Russula chloroides	40,64	0,04
Russula chloroides	41,1	-3,22
Russula chloroides	41,33	1,01
Russula chloroides	42,05	1,06
Russula chloroides	42,662	-2,359
Russula chloroides	42,974	-1,939
Russula chloroides	43,033	-2,748
Russula chloroides	43,033	-2,883
Russula chloroides	43,118	-1,9
Russula chloroides	43,249	-3,253
Russula chloroides	43,255	-2,156
Russula chloroides	43,889158	19,533868
Russula chloroides	46,122669	11,196132

Russula chloroides	40,850269	9,171227
Russula chloroides	47,81	13,34
Russula chloroides	47,63	13,22
Russula chloroides	47,77	13,05
Russula chloroides	39,07596	26,347418
Russula chloroides	51,805206	-3,134848
Russula chloroides	56,997534	-3,425469
Russula chloroides	57,154266	-3,811734
Russula chloroides	57,44973	-4,409682
Russula chloroides	58,996063	6,098393
Russula chloroides	59,572206	9,710761
Russula chloroides	59,894217	9,942284
Russula chloroides	59,913663	10,297379
Russula chloroides	61,016814	9,107201
Russula chloroides	61,732242	9,079964
Russula chloroides	62,948308	7,202467
Russula chloroides	63,016019	8,183769
Russula chloroides	63,455965	11,162556
Russula chloroides	63,596122	10,455545
Russula chloroides	58,885671	8,287668
Russula chloroides	59,705434	9,925333
Russula chloroides	61,51848	11,04696
Russula chloroides	62,932304	8,452527
Russula chloroides	62,759594	7,852884
Russula chloroides	62,825597	8,206101
Russula chloroides	63,0671	8,02404
Russula chloroides	62,478949	6,449311
Russula chloroides	62,746762	7,144095
Russula chloroides	63,1626	8,3855
Russula chloroides	63,3763	10,1751
Russula chloroides	61,571131	9,541878
Russula chloroides	60,5781	7,4844
Russula chloroides	48,780819	8,426428
Russula chloroides	58,924225	6,329026
Russula chloroides	60,84748894	10,09347561
Russula chloroides	42,14	2,53
Russula chloroides	40,36002	-7,044403
Russula chloroides	40,772747	-3,63625
Russula chloroides	45,68855	2,99338
Russula chloroides	42,98378	-1,629782
Russula chloroides	43,331	-2,944
Russula chloroides	42,217354	-0,473278
Russula chloroides	45,157985	19,782267
Russula chloroides	43,05111	11,75972
Russula chloroides	45,175861	19,499379
Russula chloroides	48,223262	16,26468

Russula chloroides	43,521731	10,409456
Russula chloroides	47,10833	15,15833
Russula chloroides	57,464321	-4,229636
Russula chloroides	51,595234	-2,363024
Russula chloroides	52,609238	-2,89561
Russula chloroides	54,22775	-4,617092
Russula chloroides	56,744522	-3,546682
Russula chloroides	56,767461	-3,842141
Russula chloroides	57,035713	-3,21271
Russula chloroides	57,074399	-2,884071
Russula chloroides	57,353706	-2,757819
Russula chloroides	57,437463	-4,57551
Russula chloroides	59,267689	9,54707
Russula chloroides	63,83909	9,706306
Russula chloroides	58,107723	7,653206
Russula chloroides	58,882111	8,997984
Russula chloroides	59,040058	5,934891
Russula chloroides	59,670755	11,019247
Russula chloroides	59,837145	10,469335
Russula chloroides	59,933503	10,858036
Russula chloroides	60,156904	10,045635
Russula chloroides	60,294785	10,54756
Russula chloroides	62,596461	10,917459
Russula chloroides	63,35295	10,559975
Russula chloroides	63,603499	10,056496
Russula chloroides	63,848261	11,355226
Russula chloroides	58,595449	7,921425
Russula chloroides	60,794056	9,250851
Russula chloroides	60,945077	10,638128
Russula chloroides	64,105298	11,738843
Russula chloroides	64,045001	11,39
Russula chloroides	59,29363	9,178303
Russula chloroides	60,578494	5,267002
Russula chloroides	63,9665	10,1099
Russula chloroides	57,5111	12,39261
Russula chloroides	58,46067	14,25229
Russula chloroides	62,3596	17,03809
Russula chloroides	62,51251	17,78367
Russula chloroides	62,51343	15,95244
Russula chloroides	62,53955	12,36492
Russula chloroides	62,61142	15,60825
Russula chloroides	62,74986	16,73791
Russula chloroides	62,81389	12,81484
Russula chloroides	63,052905	9,236846
Russula chloroides	63,6808	10,627061
Russula chloroides	48,905	17,46778

Russula chloroides	60,560613	5,042666
Russula chloroides	48,374519	9,861373
Russula chloroides	48,805851	9,781804
Russula chloroides	48,845226	9,406287
Russula chloroides	49,273933	8,040771
Russula chloroides	49,573898	6,707631
Russula chloroides	49,623894	7,3742
Russula chloroides	51,590084	10,59391
Russula chloroides	51,683945	11,126919
Russula chloroides	48,56	14,86
Russula chloroides	58,01727	7,434345
Russula chloroides	64,043663	12,169399
Russula chloroides	60,37842	15,22599
Russula chloroides	60,79447	15,03555
Russula chloroides	62,76577	15,93865
Russula chloroides	63,109454	29,807362
Russula chloroides	47,950941	-1,75473
Russula chloroides	39,368081	-8,564298
Russula chloroides	46,70375	0,52193
Russula chloroides	41,76	-5,06
Russula chloroides	48,05	13,05
Russula chloroides	43,793037	10,73429
Russula chloroides	46,8977	16,0767
Russula chloroides	50,10894	4,08266
Russula chloroides	48,4981	-3,534
Russula chloroides	51,457998	-2,828271
Russula chloroides	51,566963	-4,157671
Russula chloroides	54,623464	-5,753457
Russula chloroides	54,784043	-6,565349
Russula chloroides	58,255446	-3,445805
Russula chloroides	49,4024	2,30664
Russula chloroides	50,82911	2,17871
Russula chloroides	50,33429	2,69584
Russula chloroides	53,273044	-1,094242
Russula chloroides	57,631979	-3,701474
Russula chloroides	57,680006	-3,452163
Russula chloroides	58,150654	8,092886
Russula chloroides	58,444286	8,693599
Russula chloroides	59,060829	10,934815
Russula chloroides	54,942766	8,812513
Russula chloroides	55,073003	12,096498
Russula chloroides	55,121288	14,906285
Russula chloroides	55,21622	11,79165
Russula chloroides	55,486103	9,469461
Russula chloroides	55,594859	9,794579
Russula chloroides	55,665832	12,017823

Russula chloroides	55,669671	12,523666
Russula chloroides	55,754932	11,347373
Russula chloroides	55,898573	12,522111
Russula chloroides	55,938367	10,136034
Russula chloroides	58,316621	8,465993
Russula chloroides	58,879088	9,609081
Russula chloroides	58,958641	5,72623
Russula chloroides	59,031124	10,108481
Russula chloroides	59,105964	10,402491
Russula chloroides	59,44091	10,230652
Russula chloroides	59,697376	10,727636
Russula chloroides	59,74906	17,84678
Russula chloroides	60,087659	10,244423
Russula chloroides	60,1748	16,5401
Russula chloroides	60,749249	11,016013
Russula chloroides	59,427178	5,281813
Russula chloroides	59,328616	11,576338
Russula chloroides	50,925722	4,327123
Russula chloroides	54,674929	11,547592
Russula chloroides	54,745904	11,101699
Russula chloroides	54,763846	12,049778
Russula chloroides	54,972441	12,548117
Russula chloroides	55,34553	11,14552
Russula chloroides	55,36577	10,42642
Russula chloroides	55,47578	10,69474
Russula chloroides	55,54909	13,2018
Russula chloroides	56,15907	9,62282
Russula chloroides	56,47106	9,85357
Russula chloroides	56,64032	16,553
Russula chloroides	56,71482	15,96083
Russula chloroides	56,94168	14,80193
Russula chloroides	56,95688	18,16685
Russula chloroides	57,42007	10,46432
Russula chloroides	58,3479	15,61601
Russula chloroides	58,725579	9,263023
Russula chloroides	59,13451	16,73209
Russula chloroides	59,14948	15,48143
Russula chloroides	59,346	17,0365
Russula chloroides	59,39037	17,95707
Russula chloroides	59,452993	10,873316
Russula chloroides	59,7569	17,54945
Russula chloroides	62,678942	11,801473
Russula chloroides	55,492183	8,438325
Russula chloroides	55,069692	10,533111
Russula chloroides	55,12535	10,300675
Russula chloroides	55,133789	10,779375

Russula chloroides	56,985	10,010278
Russula chloroides	55,184053	8,94281
Russula chloroides	58,79083	11,15573
Russula chloroides	57,71906	12,13291
Russula chloroides	49,182426	7,508587
Russula chloroides	50,65411	9,058783
Russula chloroides	54,72856	10,6877
Russula chloroides	55,321112	12,347779
Russula chloroides	55,43401	10,19748
Russula chloroides	55,64483	13,66175
Russula chloroides	55,90278	13,38666
Russula chloroides	56,20261	12,80664
Russula chloroides	56,496167	14,493338
Russula chloroides	56,56971	9,04334
Russula chloroides	57,18072	16,94465
Russula chloroides	58,13914	13,61195
Russula chloroides	58,36122	11,60905
Russula chloroides	58,49789	13,70415
Russula chloroides	58,55639	13,38488
Russula chloroides	58,73084	14,79679
Russula chloroides	58,90343	17,5739
Russula chloroides	59,05468	17,21321
Russula chloroides	59,1391	17,66381
Russula chloroides	59,24692	12,37309
Russula chloroides	59,26975	14,97585
Russula chloroides	59,38787	15,56186
Russula chloroides	59,4222	16,48183
Russula chloroides	59,43842	16,78875
Russula chloroides	60,313249	5,248353
Russula chloroides	60,637761	10,597291
Russula chloroides	61,3955	16,77083
Russula chloroides	54,97471	10,00515
Russula chloroides	55,16399	9,52472
Russula chloroides	55,39854	11,38209
Russula chloroides	55,65468	14,27219
Russula chloroides	55,76332	11,63379
Russula chloroides	56,29211	10,46349
Russula chloroides	56,834115	8,969252
Russula chloroides	57,07413	9,5872
Russula chloroides	57,32429	18,32369
Russula chloroides	57,40347	15,05092
Russula chloroides	57,58341	9,99056
Russula chloroides	58,80974	12,46216
Russula chloroides	59,15972	17,89398
Russula chloroides	59,18323	14,64845
Russula chloroides	59,64344	15,07626

Russula chloroides	59,98187	18,19459
Russula chloroides	58,35703	11,9689
Russula chloroides	58,599432	9,007742
Russula chloroides	54,863617	9,494692
Russula chloroides	55,65551	11,029736
Russula chloroides	55,77159	10,59803
Russula chloroides	56,08939	13,95519
Russula chloroides	56,13544	14,40175
Russula chloroides	56,28255	13,36523
Russula chloroides	56,70177	12,90915
Russula chloroides	57,3654	18,66768
Russula chloroides	57,61557	12,93189
Russula chloroides	57,6886	18,3875
Russula chloroides	58,00848	15,92787
Russula chloroides	58,26337	14,81272
Russula chloroides	58,26683	15,26283
Russula chloroides	58,28289	12,28187
Russula chloroides	58,32595	12,52218
Russula chloroides	58,47933	11,37665
Russula chloroides	58,55158	15,09136
Russula chloroides	58,61357	14,51896
Russula chloroides	58,63289	13,14059
Russula chloroides	58,81895	13,94771
Russula chloroides	58,90161	16,24359
Russula chloroides	58,94157	14,22192
Russula chloroides	59,70752	16,12579
Russula chloroides	63,02688	14,75472
Russula chloroides	55,84581	13,11597
Russula chloroides	56,82787	14,2504
Russula chloroides	62,05909	17,18159
Russula chloroides	57,95869	12,41172
Russula chloroides	51,749352	11,445909
Russula chloroides	51,963413	10,56078
Russula chloroides	53,738865	11,426811
Russula chloroides	57,38005	16,54566
Russula chloroides	58,38369	14,00332
Russula chloroides	58,60731	16,27557
Russula chloroides	59,94785	16,60317
Russula chloroides	62,82574	15,26113
Russula chloroides	59,39849	17,5322
Russula chloroides	59,2934	18,22305
Russula chloroides	60,168316	12,035052
Russula chloroides	56,62014	15,19588
Russula chloroides	59,41629	15,97523
Russula chloroides	59,97878	15,73927
Russula chloroides	61,69533	14,74522

Russula chloroides	58,06642	11,67816
Russula chloroides	58,308156	22,072821
Russula chloroides	58,538492	24,072016
Russula chloroides	58,581239	22,69946
Russula chloroides	59,300207	24,304801
Russula chloroides	59,314286	24,558319
Russula chloroides	60,183815	24,820589
Russula chloroides	60,198366	23,983264
Russula chloroides	60,227247	25,171504
Russula chloroides	60,366732	22,134999
Russula chloroides	60,37406	25,751847
Russula chloroides	60,389027	22,364922
Russula chloroides	60,661435	23,612576
Russula chloroides	60,736852	26,415549
Russula chloroides	60,847502	23,043305
Russula chloroides	61,189422	25,680601
Russula chloroides	61,510063	23,922756
Russula chloroides	62,03129	26,765617
Russula chloroides	62,553081	26,643752
Russula chloroides	60,024736	30,672041
Russula chloroides	62,911268	27,653094
Russula chloroides	62,12879	25,680238
Russula chloroides	60,777825	23,844576
Russula chloroides	53,881272	36,986176
Russula chloroides	59,175	27,8
Russula chloroides	60,313333	24,522565
Russula chloroides	60,316462	25,432564
Russula chloroides	60,373231	23,191603
Russula chloroides	60,499668	26,224021
Russula chloroides	60,999694	23,437313
Russula chloroides	61,193459	26,048475
Russula chloroides	61,470483	23,376447
Russula chloroides	61,675485	27,325269
Russula chloroides	61,717278	29,69194
Russula chloroides	62,04334	26,144583
Russula chloroides	62,318944	26,349064
Russula chloroides	64,218009	27,751773
Russula delica	42,62	1,34
Russula delica	47,040989	12,824422
Russula delica	47,21	12,69
Russula delica	47,49	13,17
Russula delica	46,40506	11,576588
Russula delica	46,52586	11,526461
Russula delica	46,558743	11,57547
Russula delica	47	13,13
Russula delica	47,369526	11,067352

Russula delica	46,9	13,04
Russula delica	47,01	12,3
Russula delica	43,377742	41,688893
Russula delica	60,488175	6,872121
Russula delica	61,357907	7,864858
Russula delica	60,463522	7,060767
Russula delica	62,455558	8,235063
Russula delica	62,557032	8,179179
Russula delica	61,145357	8,567682
Russula delica	61,159105	8,519191
Russula delica	61,167506	8,459767
Russula delica	61,234471	7,687198
Russula delica	61,271587	7,766052
Russula delica	62,289456	7,360023
Russula delica	62,477259	8,167986
Russula delica	60,374485	6,71576
Russula delica	60,205071	6,559705
Russula delica	60,592981	7,041845
Russula delica	61,446245	7,470612
Russula delica	62,29202	7,215026
Russula delica	60,442622	6,484635
Russula delica	60,548036	6,978912
Russula delica	60,452423	6,730287
Russula delica	61,210384	6,645181
Russula delica	40,895347	-8,238311
Russula delica	36,781862	-3,109454
Russula delica	42,961379	-1,995984
Russula delica	42,294862	2,141648
Russula delica	42,04	0,98
Russula delica	42,31	0,66
Russula delica	42,45	1,25
Russula delica	42,68	1,33
Russula delica	47,38	12,55
Russula delica	47,19	13,68
Russula delica	44,37347	9,623809
Russula delica	47,72083	15,24167
Russula delica	60,477296	6,798953
Russula delica	60,53613	6,906077
Russula delica	60,555871	6,706705
Russula delica	61,14625	8,647479
Russula delica	62,105777	8,580321
Russula delica	59,430491	5,921389
Russula delica	62,011457	6,077193
Russula delica	62,863751	7,221018
Russula delica	62,889233	7,351875
Russula delica	61,071528	5,918356

Russula delica	62,575272	9,167746
Russula delica	61,306268	7,255222
Russula delica	62,411256	6,103025
Russula delica	61,17416	7,294652
Russula delica	61,865641	6,261231
Russula delica	61,948548	5,66245
Russula delica	61,202469	7,051574
Russula delica	60,36484	6,064373
Russula delica	60,460251	6,538751
Russula delica	61,193946	6,529156
Russula delica	62,34064448	9,645294522
Russula delica	42,715552	-1,744802
Russula delica	42,892033	-0,913738
Russula delica	42,914692	-2,087574
Russula delica	43,014996	-1,50444
Russula delica	43,080189	-1,837472
Russula delica	43,216069	-1,541492
Russula delica	43,244709	-1,831821
Russula delica	39,4571	-5,49157
Russula delica	38,76	-0,66
Russula delica	39,87	-0,38
Russula delica	41,32	0,9
Russula delica	42,844	-3,202
Russula delica	43,246	-2,156
Russula delica	42,446325	9,182768
Russula delica	45,178542	19,848766
Russula delica	44,39603	9,525419
Russula delica	48,215538	8,818948
Russula delica	36,752492	28,217553
Russula delica	57,385984	-4,342515
Russula delica	57,862882	-4,585866
Russula delica	51,805206	-3,134848
Russula delica	51,904811	-3,403369
Russula delica	56,849686	-3,747791
Russula delica	57,136782	-3,77783
Russula delica	62,2755	9,8328
Russula delica	58,934235	7,722924
Russula delica	59,150532	6,349876
Russula delica	59,57323	9,720697
Russula delica	61,002037	9,198506
Russula delica	61,030549	9,060992
Russula delica	62,912771	8,183032
Russula delica	63,968533	10,115578
Russula delica	64,202773	12,174903
Russula delica	61,405653	9,590234
Russula delica	59,769552	10,062335

Russula delica	59,963869	10,26786
Russula delica	60,691973	6,944148
Russula delica	60,804886	10,182664
Russula delica	61,147546	10,339348
Russula delica	62,144648	6,076043
Russula delica	62,433911	6,50577
Russula delica	62,673115	9,490162
Russula delica	62,74886	7,237843
Russula delica	62,759545	7,853746
Russula delica	60,099338	6,004368
Russula delica	62,7303	9,7209
Russula delica	64,35137	14,38983
Russula delica	58,659219	7,607892
Russula delica	58,829927	6,062442
Russula delica	51,734257	11,002121
Russula delica	62,3667	16,67226
Russula delica	65,25107	15,49174
Russula delica	59,35080235	7,336747419
Russula delica	60,241286	5,829651
Russula delica	61,10949706	9,861256767
Russula delica	62,01892419	8,931584724
Russula delica	62,22109766	5,660664329
Russula delica	63,00409592	12,12505263
Russula delica	63,599369	9,574471
Russula delica	63,610613	10,502372
Russula delica	64,82538151	13,13380252
Russula delica	61,28039747	9,223327875
Russula delica	61,5153	8,8333
Russula delica	42,14	2,52
Russula delica	40,774082	-3,642497
Russula delica	42,831604	-1,554858
Russula delica	42,939514	-1,797185
Russula delica	40,359327	-7,042931
Russula delica	42,998716	-1,345067
Russula delica	42,558684	-0,78716
Russula delica	38,52	-5,65
Russula delica	39,63	-0,32
Russula delica	41,97	3,17
Russula delica	42,15	0,51
Russula delica	42,483	-3,481
Russula delica	42,934	-2,884
Russula delica	43,710614	11,164583
Russula delica	45,060175	7,76443
Russula delica	47,78	13,04
Russula delica	43,523533	10,406422
Russula delica	47,07226	6,10719

Russula delica	45,157716	19,697105
Russula delica	48,135593	7,871511
Russula delica	48,224052	8,290742
Russula delica	44,40399	4,20456
Russula delica	51,649878	-3,40119
Russula delica	54,42818	-0,644514
Russula delica	54,952701	-2,327002
Russula delica	57,492596	-4,137912
Russula delica	56,564144	-3,616754
Russula delica	50,926408	-3,480434
Russula delica	51,158712	-3,221871
Russula delica	51,393112	-2,296745
Russula delica	51,695863	-4,189846
Russula delica	52,417767	-3,004944
Russula delica	52,609238	-2,89561
Russula delica	54,586817	-5,914826
Russula delica	54,876152	-6,686713
Russula delica	55,853412	-3,155652
Russula delica	57,297387	-5,642466
Russula delica	57,886117	-4,255778
Russula delica	52,804161	-3,076876
Russula delica	54,100034	-4,700821
Russula delica	54,689253	-7,434767
Russula delica	54,920892	-1,728502
Russula delica	54,93429	-5,980358
Russula delica	56,744522	-3,546682
Russula delica	56,76546	-3,972938
Russula delica	56,987189	-3,540277
Russula delica	57,025562	-3,327697
Russula delica	57,047568	-2,86695
Russula delica	57,058784	-2,438538
Russula delica	57,532224	-2,928529
Russula delica	59,269447	9,546118
Russula delica	59,849398	10,483133
Russula delica	60,715323	10,769839
Russula delica	64,503052	10,792935
Russula delica	59,702094	9,86935
Russula delica	60,36231	16,10536
Russula delica	60,79624	15,03667
Russula delica	61,63977	16,23836
Russula delica	62,495577	6,719695
Russula delica	62,53183	10,754361
Russula delica	62,572326	11,013884
Russula delica	62,908283	11,893272
Russula delica	63,848281	11,35378
Russula delica	58,493848	8,450274

Russula delica	58,717105	8,536879
Russula delica	59,303619	10,036625
Russula delica	62,682652	11,851375
Russula delica	58,088744	7,284987
Russula delica	59,054769	5,929824
Russula delica	59,061268	6,193974
Russula delica	59,719067	10,208392
Russula delica	59,95227	9,92262
Russula delica	60,13536	15,30798
Russula delica	60,268956	10,372963
Russula delica	61,142643	10,589385
Russula delica	62,433271	6,347999
Russula delica	63,053426	9,237376
Russula delica	63,7069	9,726599
Russula delica	64,40685	16,08208
Russula delica	49,019802	13,008966
Russula delica	50,126766	7,21773
Russula delica	50,33297	7,239363
Russula delica	50,581875	7,991554
Russula delica	59,88102	15,00977
Russula delica	62,93492	18,35861
Russula delica	63,46908	15,50634
Russula delica	62,54135	15,90171
Russula delica	63,247434	10,952511
Russula delica	63,355621	10,562113
Russula delica	65,1852	18,91723
Russula delica	60,212645	11,991521
Russula delica	60,5338	7,8073
Russula delica	61,092364	11,400764
Russula delica	58,30508	14,63031
Russula delica	58,65139	16,12522
Russula delica	61,00553	15,19064
Russula delica	62,5156	17,77873
Russula delica	62,57489	12,43426
Russula delica	62,59624	12,17985
Russula delica	62,61142	15,60825
Russula delica	62,68177	12,92765
Russula delica	62,88301	12,68977
Russula delica	63,21385	13,06852
Russula delica	63,28505	12,33232
Russula delica	63,53059	13,19446
Russula delica	63,563746	9,919161
Russula delica	63,7341	14,26751
Russula delica	65,01419	14,29136
Russula delica	60,2988	10,6971
Russula delica	61,97144	16,92007

Russula delica	48,441441	9,763528
Russula delica	48,745522	8,546462
Russula delica	48,792759	9,287395
Russula delica	48,899155	13,370917
Russula delica	49,961658	9,75071
Russula delica	50,548073	10,181708
Russula delica	51,425331	9,738264
Russula delica	51,590084	10,59391
Russula delica	51,834824	10,379591
Russula delica	62,66892	17,39785
Russula delica	62,71724	15,41849
Russula delica	63,156981	10,04595
Russula delica	64,180054	11,27206
Russula delica	64,86581	14,01666
Russula delica	60,61435	15,67812
Russula delica	62,76771	15,94756
Russula delica	60,56699	15,25955
Russula delica	61,42508	16,59639
Russula delica	62,52494	15,34848
Russula delica	62,7213	7,449
Russula delica	63,04762	15,66712
Russula delica	58,17005445	7,052184406
Russula delica	60,51107981	10,51920363
Russula delica	60,629405	5,206168
Russula delica	60,66317298	9,227254418
Russula delica	60,844993	10,536457
Russula delica	60,94589325	10,13367286
Russula delica	61,228	11,8406
Russula delica	62,155229	9,259064
Russula delica	62,24184044	9,643199037
Russula delica	63,361171	10,309393
Russula delica	63,650338	11,514499
Russula delica	64,279681	12,531672
Russula delica	64,476207	11,527656
Russula delica	64,57366496	12,50369078
Russula delica	60,160301	12,289951
Russula delica	59,0774	9,2625
Russula delica	61,90327	12,87462
Russula delica	63,082715	29,836306
Russula delica	40,251118	-4,285975
Russula delica	41,506722	-4,426811
Russula delica	46,70375	0,52193
Russula delica	43,434535	3,446806
Russula delica	43,308	-1,983
Russula delica	43,419	-1,474
Russula delica	43,716	-1,43

Russula delica	40,847666	17,101959
Russula delica	48,239777	12,752907
Russula delica	46,92083	15,60833
Russula delica	41,195235	28,949051
Russula delica	50	3
Russula delica	53,650735	-1,240413
Russula delica	54,68614	-6,65727
Russula delica	55,638401	-4,634671
Russula delica	55,796019	-6,244537
Russula delica	57,594727	-3,715712
Russula delica	55,873437	-4,28672
Russula delica	48,4981	-3,534
Russula delica	48,82992	-3,07617
Russula delica	51,338189	-2,561966
Russula delica	51,444554	-3,232365
Russula delica	52,793878	-1,457924
Russula delica	53,607856	-1,576027
Russula delica	54,554046	-5,601139
Russula delica	54,727612	-6,300351
Russula delica	50,46299	1,71197
Russula delica	50,072501	-5,280443
Russula delica	50,801704	-1,403289
Russula delica	50,82911	2,17871
Russula delica	51,603961	-2,297396
Russula delica	51,802652	-0,587384
Russula delica	51,820108	-1,936118
Russula delica	51,836907	-2,531146
Russula delica	52,38593	-2,376092
Russula delica	52,628216	-2,496358
Russula delica	52,895855	-2,85617
Russula delica	54,190666	-2,790759
Russula delica	54,334649	-6,840093
Russula delica	55,054149	-6,928914
Russula delica	57,400698	-2,226292
Russula delica	58,305622	-5,13345
Russula delica	50,927638	4,331177
Russula delica	58,423689	8,688694
Russula delica	59,863639	10,827179
Russula delica	55,82135	12,311087
Russula delica	59,65179	17,36858
Russula delica	55,305399	12,00651
Russula delica	55,74983	12,57998
Russula delica	55,95216	13,33748
Russula delica	56,126769	10,209945
Russula delica	56,39085	9,3437
Russula delica	58,15336	7,673733

Russula delica	58,273362	8,416666
Russula delica	58,719712	9,082264
Russula delica	59,010284	9,71307
Russula delica	59,02939	10,111024
Russula delica	59,106093	10,885641
Russula delica	59,23883	18,26407
Russula delica	59,261362	10,260476
Russula delica	59,31984	17,87503
Russula delica	59,77852	17,67116
Russula delica	60,26385	5,254641
Russula delica	61,34899	17,05623
Russula delica	63,89073	20,16288
Russula delica	64,929401	10,886521
Russula delica	58,735772	8,853517
Russula delica	58,851471	9,460751
Russula delica	59,730054	5,368099
Russula delica	54,76282	11,3577
Russula delica	55,077188	12,095493
Russula delica	55,31795	12,345878
Russula delica	55,56054	13,2202
Russula delica	55,62345	11,92385
Russula delica	55,67655	9,852312
Russula delica	55,836139	11,484142
Russula delica	56,029105	9,967128
Russula delica	56,10085	14,22015
Russula delica	56,19438	14,98809
Russula delica	56,47387	9,84084
Russula delica	56,492093	10,757611
Russula delica	56,60547	14,16769
Russula delica	56,64966	16,59291
Russula delica	56,72389	15,95469
Russula delica	56,76833	14,77094
Russula delica	56,8585	9,86167
Russula delica	57,13278	9,40187
Russula delica	57,36811	18,7675
Russula delica	57,5009	18,44822
Russula delica	58,147895	8,106325
Russula delica	58,3587	15,68021
Russula delica	58,44205	13,65765
Russula delica	58,61627	14,50995
Russula delica	58,76376	17,86176
Russula delica	58,955505	5,724065
Russula delica	58,98439	14,10348
Russula delica	59,04917	15,95337
Russula delica	59,06343	17,21126
Russula delica	59,08259	16,84283

Russula delica	59,13003	12,82442
Russula delica	59,14109	15,6861
Russula delica	59,21085	17,62176
Russula delica	59,23888	14,92947
Russula delica	59,34742	15,23034
Russula delica	59,57064	16,84606
Russula delica	59,57131	16,51387
Russula delica	59,578711	10,653247
Russula delica	59,91203	18,89504
Russula delica	60,121682	10,190839
Russula delica	60,220463	11,116885
Russula delica	60,31714	15,39058
Russula delica	60,562	17,44114
Russula delica	60,63873	17,1765
Russula delica	63,14151	14,7573
Russula delica	63,15342	14,43806
Russula delica	63,59534	19,61652
Russula delica	63,97348	17,95129
Russula delica	63,9853	11,5787
Russula delica	56,11013	9,667011
Russula delica	54,722003	11,79885
Russula delica	55,353424	10,438997
Russula delica	57,71906	12,13291
Russula delica	49,185829	7,515645
Russula delica	49,356998	7,780724
Russula delica	54,84592	10,77527
Russula delica	55,50303	13,47375
Russula delica	55,65563	14,26866
Russula delica	55,67433	9,524874
Russula delica	56,01613	13,68316
Russula delica	56,046816	12,554768
Russula delica	56,14377	15,77993
Russula delica	56,33436	16,42096
Russula delica	56,33619	14,13487
Russula delica	56,64616	15,0893
Russula delica	56,68891	9,12352
Russula delica	56,7371	8,80799
Russula delica	57,19554	16,93316
Russula delica	57,40361	15,05
Russula delica	58,01345	12,4271
Russula delica	58,21953	12,0319
Russula delica	58,36122	11,60905
Russula delica	58,72883	17,1065
Russula delica	58,74446	11,22046
Russula delica	58,85633	16,28642
Russula delica	58,9024	17,57418

Russula delica	59,24692	12,37309
Russula delica	59,58527	13,78806
Russula delica	60,13287	17,33317
Russula delica	60,15526	17,82553
Russula delica	60,26197	18,31752
Russula delica	60,713464	11,185849
Russula delica	58,80998	12,48242
Russula delica	59,65831	18,11677
Russula delica	55,09973	10,70826
Russula delica	55,120846	14,945998
Russula delica	55,43347	10,19583
Russula delica	55,65595	11,03334
Russula delica	55,95952	12,88796
Russula delica	57,12447	12,57156
Russula delica	57,13275	18,37542
Russula delica	57,26169	14,46439
Russula delica	57,85405	18,99084
Russula delica	58,29369	12,48029
Russula delica	59,10584	16,58686
Russula delica	59,18258	14,6494
Russula delica	59,80776	12,17483
Russula delica	59,84409	16,81679
Russula delica	60,17033	16,53172
Russula delica	60,27855	16,8492
Russula delica	61,05334	17,14726
Russula delica	63,36917	14,94525
Russula delica	59,104763	11,281277
Russula delica	60,782395	9,010099
Russula delica	60,886284	11,567739
Russula delica	54,97545	12,54742
Russula delica	56,651792	9,577521
Russula delica	56,834	10,24364
Russula delica	58,16973	16,25971
Russula delica	59,31703	13,12778
Russula delica	59,58507	15,17028
Russula delica	61,43802	16,40254
Russula delica	55,56222	13,83719
Russula delica	56,22601	13,17546
Russula delica	56,2508	15,42646
Russula delica	56,3315	14,38419
Russula delica	56,34207	14,69381
Russula delica	56,67404	13,88757
Russula delica	56,70177	12,90915
Russula delica	56,98518	15,7034
Russula delica	57,04161	15,36983
Russula delica	57,36735	16,13842

Russula delica	57,456457	12,314181
Russula delica	57,47216	12,59405
Russula delica	57,51849	16,41428
Russula delica	57,61557	12,93189
Russula delica	57,92264	13,50306
Russula delica	57,94088	16,03971
Russula delica	58,1504	13,76299
Russula delica	58,19449	16,58736
Russula delica	58,26337	14,81272
Russula delica	58,34268	15,11742
Russula delica	58,42104	14,14912
Russula delica	58,42214	16,60984
Russula delica	58,50007	13,15496
Russula delica	58,54152	12,12056
Russula delica	58,5673	13,90533
Russula delica	58,71298	16,79686
Russula delica	58,74956	15,85423
Russula delica	58,82618	15,40079
Russula delica	59,36724	18,50117
Russula delica	59,5853	12,96231
Russula delica	59,68206	18,75394
Russula delica	59,73313	16,11341
Russula delica	59,90147	16,56819
Russula delica	59,98569	15,74494
Russula delica	60,96923	14,53739
Russula delica	62,34898	16,14832
Russula delica	62,46205	17,29429
Russula delica	63,30136	13,96693
Russula delica	63,62252	15,96728
Russula delica	59,75181	18,38922
Russula delica	54,863864	9,495964
Russula delica	55,316	11,17745
Russula delica	55,93743	10,57725
Russula delica	56,4058	15,81404
Russula delica	56,46407	12,93147
Russula delica	56,908	16,2465
Russula delica	57,37928	15,54413
Russula delica	57,5149	10,30501
Russula delica	57,72691	11,76165
Russula delica	48,889977	9,614968
Russula delica	49,323929	8,290735
Russula delica	49,423916	6,874273
Russula delica	50,114414	8,875923
Russula delica	50,654469	9,058828
Russula delica	51,536087	10,793724
Russula delica	51,616657	7,950153

Russula delica	51,963413	10,56078
Russula delica	52,492657	9,583597
Russula delica	53,56356	8,769836
Russula delica	53,784428	11,109452
Russula delica	53,989025	10,827223
Russula delica	54,417732	10,066051
Russula delica	54,421326	9,656982
Russula delica	55,17651	10,0554
Russula delica	55,20409	11,43895
Russula delica	55,43486	10,6882
Russula delica	55,49314	9,077791
Russula delica	55,75009	9,220488
Russula delica	56,28629	10,49559
Russula delica	56,8983	13,33816
Russula delica	58,01978	12,8224
Russula delica	58,19105	14,09981
Russula delica	58,33596	12,89826
Russula delica	58,37603	11,3297
Russula delica	59,13097	16,36178
Russula delica	59,405	14,03035
Russula delica	59,88406	17,34785
Russula delica	63,66714	14,67145
Russula delica	64,4422	19,05394
Russula delica	57,61572	13,54632
Russula delica	57,49131	14,61567
Russula delica	59,314961	11,768985
Russula delica	57,14214	14,172
Russula delica	57,97585	11,94236
Russula delica	58,06642	11,67816
Russula delica	58,98311	15,09181
Russula delica	59,35786	14,3251
Russula delica	59,476001	5,246143
Russula delica	62,46661	16,93095
Russula delica	65,06021	17,16915
Russula delica	59,50946	12,54556
Russula delica	61,33671303	10,55897083
Russula delica	64,24858	21,06661
Russula delica	58,461039	21,998808
Russula delica	59,17027	23,526723
Russula delica	59,269261	24,155513
Russula delica	60,192897	25,053753
Russula delica	60,240721	23,799192
Russula delica	60,367794	25,70485
Russula delica	60,41594	22,911649
Russula delica	60,428152	22,314185
Russula delica	60,444035	24,847439

Russula delica	60,529277	25,08324
Russula delica	60,628614	23,51311
Russula delica	60,84755	23,042884
Russula delica	60,859182	26,699537
Russula delica	61,450058	23,744806
Russula delica	61,468431	23,375328
Russula delica	62,878262	27,646891
Russula delica	63,0795	21,687
Russula delica	52,108414	35,759022
Russula delica	53,967378	28,06865
Russula delica	55,829762	37,70776
Russula delica	56,735254	37,174983
Russula delica	54,28918	37,868092
Russula delica	58,45344	22,56116
Russula delica	55,523989	37,601563
Russula delica	56,763249	36,779622
Russula delica	55,818771	37,283817
Russula delica	52,320509	35,25491
Russula delica	57,767145	34,887545
Russula delica	62,262285	25,701857
Russula delica	62,318591	26,350243
Russula delica	63,214524	28,037375
Russula delica	60,014729	30,355309
Russula delica	53,175043	34,144006
Russula delica	58,28333	27,31667
Russula delica	61,9259	25,6925
Russula delica	59,175	27,8
Russula delica	60,965988	25,732163
Russula delica	62,112437	28,500515
Russula delica	62,879467	28,02848
Russula delica	63,837035	23,124296
Russula delica	58,059233	52,606672
Russula delica	55,500308	47,421275
Russula delica	54,606129	52,442783
Russula delica	56,17087	47,314228
Russula delica	57,213468	41,931112
Russula delica	56,4346	43,89993
Russula delica	53,668262	46,443741
Russula delica	56,174428	44,015484
Russula delica	58,539191	50,056506
Russula delica	55,806159	52,424482
Russula delica	58,563133	50,352086
Russula delica	61,8253	56,8446
Russula delica	57,494132	57,033238
Russula delica	57,848882	55,81601
