# Supplementary material for: Loss of fungal symbionts and changes in pollinator availability caused by climate change will affect the distribution and survival chances of myco-heterotrophic orchid species
Source: Sci Rep. 2023 Apr 26;13:6848. doi: 10.1038/s41598-023-33856-y (PMC10133392; doi:10.1038/s41598-023-33856-y)
Supplement: Supplementary file 3 — Supplementary Information 3. [file 41598_2023_33856_MOESM3_ESM.pdf]

Loss of fungal symbionts and changes in pollinator availability caused by climate change will affect the distribution and survival chances of myco-heterotrophic orchid species

Marta Kolanowska[1]

[1] University of Lodz, Faculty of Biology and Environmental Protection, Department of Geobotany and Plant Ecology, Banacha 12/16, 90-237 Lodz, Poland (martakolanowska@wp.pl, ORCID: 0000-0001-5347-5403)

Supplementary Annex 3. Absolute value of R Pearson's correlation index calculated for bioclimatic variables.

Absolute value of R Pearson's correlation index

	bio1	bio2	bio3	bio4	bio5	bio6	bio7	bio8	bio9	bio10	bio11	bio12	bio13	bio14	bio15	bio16	bio17	bio18	bio19
bio1	x	0,807	0,855	0,463	0,911	0,919	0,078	0,274	0,889	0,941	0,963	0,690	0,624	0,679	0,628	0,631	0,683	0,819	0,389
bio2	x	x	0,762	0,132	0,900	0,581	0,348	0,187	0,733	0,848	0,700	0,785	0,721	0,764	0,567	0,729	0,776	0,816	0,552
bio3	x	x	x	0,716	0,672	0,880	0,321	0,058	0,802	0,679	0,916	0,505	0,459	0,505	0,537	0,462	0,508	0,678	0,219
bio4	x	x	x	x	0,085	0,751	0,874	0,114	0,470	0,138	0,683	0,087	0,091	0,053	0,228	0,099	0,062	0,144	0,258
bio5	x	x	x	x	x	0,690	0,330	0,310	0,812	0,988	0,777	0,816	0,741	0,807	0,617	0,752	0,813	0,879	0,542
bio6	x	x	x	x	x	x	0,456	0,148	0,851	0,744	0,986	0,430	0,379	0,446	0,542	0,381	0,442	0,645	0,125
bio7	x	x	x	x	x	x	x	0,188	0,111	0,246	0,330	0,443	0,417	0,411	0,051	0,428	0,423	0,239	0,503
bio8	x	x	x	x	x	x	x	x	0,037	0,343	0,185	0,358	0,364	0,225	0,092	0,373	0,240	0,079	0,494
bio9	x	x	x	x	x	x	x	x	x	0,821	0,879	0,555	0,475	0,628	0,671	0,483	0,619	0,819	0,187
bio10	x	x	x	x	x	x	x	x	x	x	0,818	0,801	0,728	0,781	0,619	0,739	0,788	0,864	0,525
bio11	x	x	x	x	x	x	x	x	x	x	x	0,538	0,482	0,543	0,586	0,486	0,543	0,721	0,234
bio12	x	x	x	x	x	x	x	x	x	x	x	x	0,957	0,893	0,482	0,967	0,912	0,843	0,864
bio13	x	x	x	x	x	x	x	x	x	x	x	x	x	0,754	0,307	0,996	0,776	0,751	0,893
bio14	x	x	x	x	x	x	x	x	x	x	x	x	x	x	0,638	0,771	0,994	0,897	0,641
bio15	x	x	x	x	x	x	x	x	x	x	x	x	x	x	x	0,328	0,644	0,614	0,204
bio16	x	x	x	x	x	x	x	x	x	x	x	x	x	x	x	x	0,792	0,763	0,899
bio17	x	x	x	x	x	x	x	x	x	x	x	x	x	x	x	x	x	0,895	0,669
bio18	x	x	x	x	x	x	x	x	x	x	x	x	x	x	x	x	x	x	0,484
bio19	x	x	x	x	x	x	x	x	x	x	x	x	x	x	x	x	x	x	x
