# Supplementary material for: Loss of fungal symbionts and changes in pollinator availability caused by climate change will affect the distribution and survival chances of myco-heterotrophic orchid species
Source: Sci Rep. 2023 Apr 26;13:6848. doi: 10.1038/s41598-023-33856-y (PMC10133392; doi:10.1038/s41598-023-33856-y)

Loss of fungal symbionts and changes in pollinator availability caused by climate change will affect the distribution and survival chances of myco-heterotrophic orchid species

Marta Kolanowska[1]

[1] University of Lodz, Faculty of Biology and Environmental Protection, Department of Geobotany and Plant Ecology, Banacha 12/16, 90-237 Lodz, Poland (martakolanowska@wp.pl, ORCID: 0000-0001-5347-5403)

Supplementary Annex 6. Changes in the orchid pollinators distribution. (a) SSP1-2.6 scenario, (b) SSP2-4.5 scenario, (c) SSP3-7.0 scenario, (d) SSP5-8.5 scenario. Legend: -1 – range expansion, 0 – no occurrence, 1 – present, 2 – range contraction. Maps generated by the author in ArcGIS [47,48].

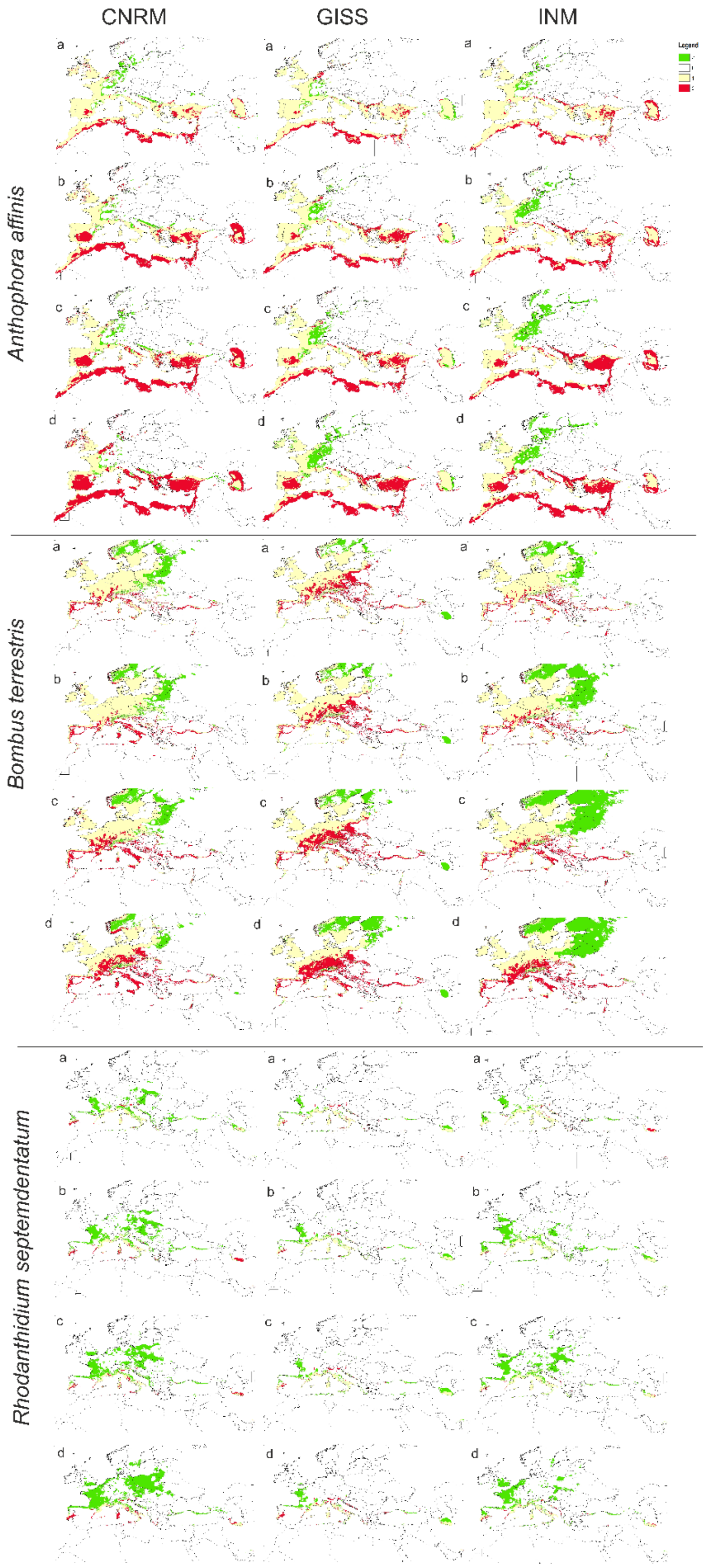

Supplement: Supplementary file 6 — Supplementary Information 6. [file 41598_2023_33856_MOESM6_ESM.pdf]
